# Enhancing Non-small Cell Lung Cancer Susceptibility to Anti-PD-1/PD-L1 Therapy through PD-L1 Ligand–Ir(III) Complex Conjugates

**DOI:** 10.34133/cancomm.0029

**Published:** 2026-05-13

**Authors:** Valentina Pagliara, Giulia Assoni, Giovanna Polcaro, Alberto Bossi, Marta Penconi, Luigi Liguori, Pierfausto Seneci, Francesco Saverio Di Leva, Vincenzo Maria D’Amore, Diego Brancaccio, Federica Santoro, Cristina R. Ferrone, Elena Ciaglia, Annibale Alessandro Puca, Stefano Pepe, Luciana Marinelli, Daniela Arosio, Francesco Sabbatino

**Affiliations:** ^1^ Department of Medicine, Surgery and Dentistry, University of Salerno, Baronissi 84081, Italy.; ^2^ Department of Cellular, Computational and Integrative Biology (CIBIO), University of Trento, I-38123 Trento, Italy.; ^3^ Department of Chemistry, University of Milan, Milan 20133, Italy.; ^4^ Istituto di Scienze e Tecnologie Chimiche “Giulio Natta” (SCITEC), Consiglio Nazionale delle Ricerche (CNR), Milan 20133, Italy.; ^5^ Department of Surgery, Cedars-Sinai Medical Center, Los Angeles 90048, CA, USA.; ^6^ Department of Pharmacy, University of Naples “Federico II”, Naples 80131, Italy.

Immunotherapy targeting programmed cell death protein 1 (PD-1) and programmed death ligand 1 (PD-L1) has transformed the management of several types of cancers, including non-oncogene-addicted non-small cell lung cancer (NSCLC) [1], although its efficacy remains limited by resistance mechanisms and constraints inherent to monoclonal antibodies [1]. To overcome these drawbacks, small-molecule PD-L1 inhibitors have been developed, and we previously contributed by identifying the nanomolar triazine-based ligand Tr-10 [2]. In parallel, combinatorial strategies aimed at improving the efficacy of anti-PD-1/PD-L1 immunotherapy have gained increasing attention. Notably, platinum-based chemotherapy combined with immune checkpoint inhibitors is recommended as a first-line treatment for advanced NSCLC with PD-L1 expression <50% [3]. Here, we investigated a novel combination involving our anti-PD-L1, Tr-10 [2], and a bis(phenyl-pyridine)iridium(III) complex, Ir-2 (Fig. 1A) [4]. Iridium (Ir) complexes, unlike platinum drugs, are chemically inert and induce endoplasmic reticulum (ER) stress and overproduction of reactive oxygen species (ROS) [5,6], both culminating in damage-associated molecular pattern (DAMP) release and immunogenic cell death (ICD). Moreover, their photophysical properties enable PD-L1-targeted bioimaging when coupled with PD-L1 ligands (Fig. S1) [7].

**Fig. 1.**
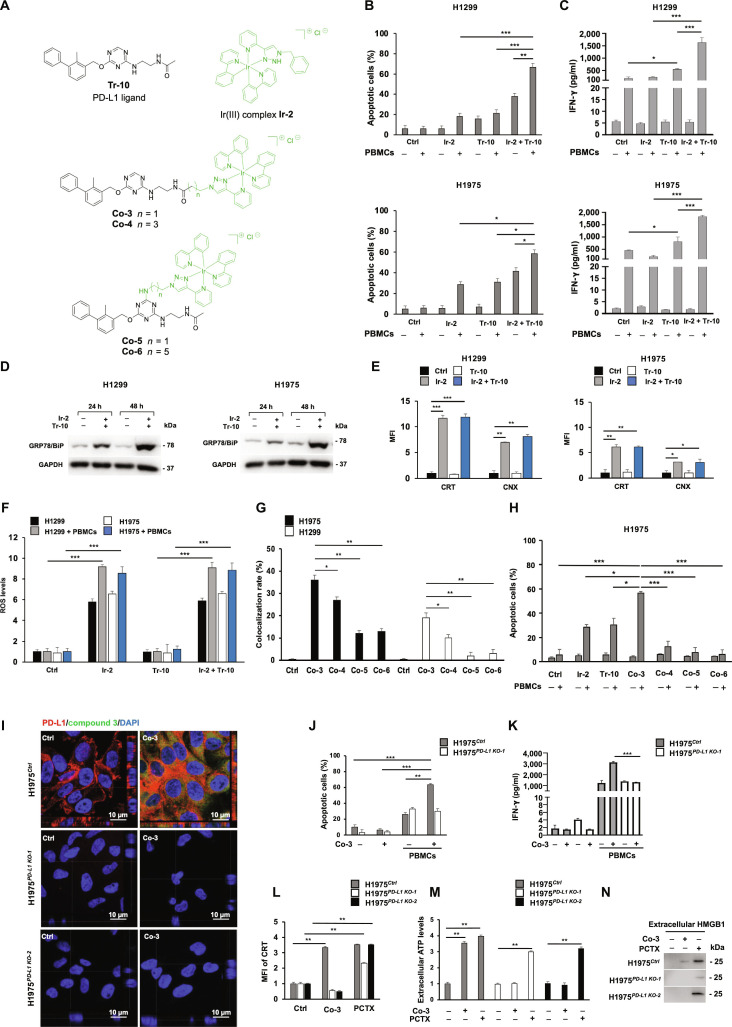
Selectively enhancing non-oncogene-addicted NSCLC susceptibility to anti-PD-1/PD-L1-ligand based immunotherapy through anti-PD-L1 ligand–Ir(III) complex conjugates. (A) Chemical structures of the PD-L1 ligand Tr-10, the Ir-2 Ir(III) complex, and PD-L1 ligand–Ir(III) complex conjugates with different chain lengths Co-3 (*n* = 1), Co-4 (*n* = 3), Co-5 (*n* = 1), and Co-6 (*n* = 5). (B) Apoptosis induction of H1299 and H1975 cells co-cultured with activated PBMCs in the presence of Ir-2 (1 μmol/l) and/or Tr-10 (10 μmol/l). Following 48-h incubation, apoptosis induction was determined by flow cytometry analysis of annexin V and PI staining. The levels of apoptosis are plotted and expressed as a mean fraction of annexin V^+^ cells ± SD of the results. (C) IFN-γ release by activated PBMCs co-cultured with H1299 and H1975 cells in the presence of Ir-2 (1 μmol/l) and/or Tr-10 (10 μmol/l). Following 48-h incubation, IFN-γ levels in the medium harvested from cultures of PBMCs with cancer cells were measured by an ELISA Max Deluxe Set Human IFN-γ kit. Data are expressed as IFN-γ levels ± SD of the results obtained in 3 independent experiments. (D) GRP78/BiP ER stress marker assessed in H1299 and H1975 cells in the presence of Ir-2 (1 μmol/l) and Tr-10 (10 μmol/l). Following a 24- and 48-h incubation, cells were harvested and lysed. GRP78/BiP expression levels were determined by Western blotting. (E) Cell surface exposure of CRT and CNX in H1299 and H1975 cells in the presence of Ir-2 (1 μmol/l) and/or Tr-10 (10 μmol/l). Following 48-h incubation, cells were harvested and the cell surface was stained with CRT- and CNX-specific mAbs. Mouse IgG was used as a specificity control of mouse antibodies. Cell staining was detected by R-PE-conjugated F(ab′)2 fragment goat anti-mouse IgG. Data are expressed as MFI ± SD of the results obtained in 3 independent experiments. (F) ROS production measured in H1299 and H1975 co-cultured with activated PBMCs in the presence of Ir-2 (1 μmol/l) and/or Tr-10 (10 μmol/l). Following 48-h incubation, ROS quantification was carried out using MitoSOX fluorescence by flow cytometry. Data show mean values ± SD of ROS levels calculated on 3 independent experiments and expressed as fold change vs. untreated cells (Ctrl), set as 1. (G) Cellular uptake of Ir(III) complex conjugates Co-3 to Co-6 in H1975 and H1299 cells. Following 3-h incubation with the IC_50_ value of all conjugates, cellular uptake was determined by confocal microscope. After acquisition, the colocalization rate of endogenous PD-L1 vs. Ir(III) complex signals was measured using the Leica SP5 software by quantitative analysis of the number of colocalized pixels in the image. Measurement on 30 cells for each experimental point was utilized to reduce intrinsic variability. Data are shown as mean percentage ± SD of the colocalization pixels. (H) Apoptosis induction of H1975 cells co-cultured with activated PBMCs in the presence of Ir-2 (1 μmol/l), Tr-10 (10 μmol/l), or Co-3 to Co-6 (1 μmol/l). Following 48-h incubation, apoptosis induction was determined by flow cytometry analysis of annexin V and PI staining. The levels of apoptosis are plotted and expressed as a mean fraction of annexin V^+^ cells ± SD of the results. (I) Cellular uptake of Co-3 in H1975*^Ctrl^*, H1975*^PD-L1 KO-1^*, and H1975*^PD-L1 KO-2^* cells. Cells were incubated with the IC_50_ (32.4 μmol/l) of Co-3. Following 3-h incubation, cell uptake was determined by confocal microscopy. Cells were stained with PD-L1 primary specific mAb, followed by a Cy5-linked secondary antibody (red), and counterstained with DAPI (blue). The wavelength emission of Co-3 was read using the FITC channel (green). Representative images from a confocal Z-stack with orthogonal side views of H1975*^Ctrl^*, H1975*^PD-L1 KO-1^*, and H1975*^PD-L1 KO-2^* cells are shown. The orthogonal views (XZ and YZ projections) were reconstructed from confocal Z-stacks, showing the axial (*z*-axis) distribution of the signal. (J) Apoptosis induction of H1975*^Ctrl^* and H1975*^PD-L1 KO-1^* cells cultured in the presence or absence of activated PBMCs with or without Co-3 (1 μmol/l). Following 48-h incubation, apoptosis induction was determined by flow cytometry analysis of annexin V and PI staining. The levels of apoptosis are plotted and expressed as a mean fraction of annexin V^+^ cells ± SD of the results obtained in 3 independent experiments. (K) IFN-γ release by activated PBMCs co-cultured with H1975*^Ctrl^* and H1975*^PD-L1 KO-1^* cells in the presence of Co-3 (1 μmol/l), compared to control conditions without activated PBMCs and without Co-3 treatment. Following 48 h of incubation, the IFN-γ levels in the medium harvested from cultures of PBMCs with cancer cells were measured by an ELISA Max Deluxe Set Human IFN-γ kit. Data are expressed as IFN-γ levels ± SD of the results obtained in 3 independent experiments. (L) Cell surface exposure of CRT in H1975*^Ctrl^*, H1975*^PD-L1 KO-1^*, and H1975*^PD-L1 KO-2^* cells in the presence of Co-3 (1 μmol/l). Untreated cells (Ctrl) and PCTX (50 nmol/l)-treated cells were included as negative and ICD-positive controls, respectively. Following 48-h incubation, cells were surface-stained with CRT-specific mAb. Cell staining was detected by PE-conjugated F(ab′)2 fragment goat anti-mouse IgG. Mouse IgG was used as a specificity control of the mouse antibody. Data are expressed as fold changes of CRT mean fluorescence intensity ± SD vs. untreated cells (Ctrl), set as 1. (M) ATP release in H1975*^Ctrl^*, H1975*^PD-L1 KO-1^*, and H1975*^PD-L1 KO-2^* cells in the presence of Co-3 (1 μmol/l). Untreated cells (Ctrl) and PCTX (50 nmol/l)-treated cells were included as negative and ICD-positive controls, respectively. Following 24 h of incubation, extracellular ATP levels (pg/ml) in the culture media were measured using the ATP Assay Kit. Data are expressed as fold changes of ATP levels ± SD vs. untreated cells set as 1. (N) HMGB1 release in H1975*^Ctrl^*, H1975*^PD-L1 KO-1^*, and H1975*^PD-L1 KO-2^* cells in the presence of Co-3 (1 μmol/l). Untreated cells (Ctrl) and PCTX (50 nmol/l)-treated cells were included as negative and ICD-positive controls, respectively. Following 48-h incubation, extracellular HMGB1 levels from culture media were analyzed by Western blotting. Representative images are shown. *, **, and *** indicate *P* < 0.05, < 0.01, and < 0.001, respectively. NSCLC, non-small cell lung cancer; PD-1, programmed cell death protein 1; PD-L1, programmed death ligand 1; PBMCs, peripheral blood mononuclear cells; ATP, adenosine triphosphate; HMGB1, high mobility group box 1; CRT, calreticulin; CNX, calnexin; IFN-γ, interferon-gamma; FITC, fluorescein isothiocyanate; DAPI, 4′,6-diamidino-2-phenylindole; PE, phycoerythrin; ICD, immunogenic cell death; MFI, mean fluorescence intensity; PI, propidium iodide; ELISA, enzyme-linked immunosorbent assay; GRP78/BiP, glucose-regulated protein 78/binding immunoglobulin protein; SD, standard deviation; PCTX, paclitaxel; mAb, monoclonal antibody; IgG, immunoglobulin G; Ctrl, control; ROS, reactive oxygen species; KO, knockout.

Firstly, we assessed the cytotoxicity of Tr-10 [2] and/or Ir-2 in NSCLC cell lines (H1975 and H1299) expressing different PD-L1 levels, both under basal conditions and following interferon-gamma (IFN-γ) incubation (Figs. S2 and S3). Human leukocyte antigen (HLA)-matched peripheral blood mononuclear cells (PBMCs) and normal immortalized human keratinocytes (HaCaT) were used as noncancer cell models. While Tr-10 was well tolerated up to 10 μmol/l, increased cytotoxicity was observed with doses of Ir-2 higher than 1 and 5 μmol/l in noncancer and cancer cells, respectively, regardless of PD-L1 expression (Figs. S2 and S3). Thus, 10 μmol/l for Tr-10 and 1 μmol/l for Ir-2 were chosen as effective concentrations to study the anticancer effect of the 2 compounds alone and in combination. This was further supported by drug–drug interaction analysis, which showed a moderate synergistic effect of 10 μmol/l Tr-10 and 1 μmol/l Ir-2 in both H1975 and H1299 cells (synergy scores = 5.02 and 7.35, respectively; Fig. S4). Tr-10, through PD-L1 blockade [2], was shown to induce apoptosis in NSCLC cells and to increase the release of IFN-γ from HLA-matched PBMCs co-cultured with NSCLC cells, as compared to untreated cells in the presence of PBMCs, thus enhancing antitumor immune responses (Fig. 1B and C and Figs. S5 and S6). Importantly, Ir-2 synergistically enhanced the immunomodulatory ability of Tr-10 in increasing the recognition and destruction of NSCLC cells (H1299, H1975, HCC827, and H1703) co-cultured with activated HLA-matched PBMCs (Fig. 1B and C and Figs. S5 and S6). Notably, the combination had minimal effects on noncancer cells (HaCaT and Beas-2B), highlighting selective cancer cell susceptibility to Ir-2-mediated effect (Fig. S6). By investigating the mechanisms underlying the observed synergism, we found that treatment with Ir-2, either alone or in combination with Tr-10, induced a time-dependent up-regulation of ER chaperone glucose-regulated protein 78 (GRP78), also known as binding immunoglobulin protein, a hallmark of ER stress [8], in NSCLC cells (Fig. 1D and Fig. S7). Prolonged ER stress promotes DAMP release, including calreticulin (CRT), adenosine triphosphate (ATP), and high mobility group box 1 (HMGB1), as well as cell surface exposure of CRT [9]. Accordingly, incubation with Ir-2 alone or in combination with Tr-10 caused an increase in CRT exposure by NSCLC cells (Fig. 1E). Increased release of ATP, HMGB1, and CRT further validated DAMP release by Ir-2 in NSCLC cells (Fig. S8). An analysis of other relevant immunological effects demonstrated that treatment with Ir-2, alone or in combination with Tr-10, promoted the translocation of the ER chaperone calnexin from the ER to the plasma membrane in a time-dependent manner (Fig. 1E and Fig. S9), while no significant changes were observed in the expression levels of HLA classes I and II, β2-microglobulin (Fig. S10), and PD-L1 (Fig. S11). These findings highlight that enhanced recognition and destruction of cancer cells by Ir-2 is mediated by ER stress more than by modulation of HLA class I antigen or PD-L1 expression. As compared to untreated cells, we also observed a marked alteration of mitochondrial morphology in NSCLC cells treated with Ir-2 and Tr-10, characterized by the loss of the canonical tubular network and increased fragmentation. Consistently, Ir-2, alone or in combination with Tr-10, markedly increased ROS production regardless of PBMC presence, whereas Tr-10 alone had no effect (Fig. 1F and Fig. S12). Notably, Ir-based compounds have been reported to induce ER stress and mitochondrial dysfunction, leading to ROS overproduction and impairment of mitochondrial membrane integrity [10]. In line with this, our findings also suggest that Ir-2 treatment triggers a sequential cascade of biological events in NSCLC cells, such as (a) ER stress induction and CRT cell surface translocation, (b) mitochondrial dysfunction and increased ROS production, and, ultimately, (c) a progressive DAMP release and enhanced immunogenicity. As a result, Ir-2 treatment promotes ICD and increases cancer cell susceptibility to PBMC-mediated killing.

Given the observed synergistic effect, we thought to integrate both molecules (Ir-2 and Tr-10) into a single structural framework, generating conjugates Co-3 to Co-6 (Fig. 1A and Fig. S1). This strategy would allow the preferential delivery of Ir-2 into PD-L1-expressing cancer cells and, by enabling both molecules to reach the same cells simultaneously, achieve maximum synergy, potentially reducing anti-PD-L1 resistance and limiting Ir-2 off-target toxicity.

Incubation of noncancer and cancer cells with increasing concentrations (1 to 100 μmol/l) of the novel conjugates Co-3 to Co-6 was performed to determine their nontoxic concentrations in both NSCLC and noncancer cells (Fig. S13 and Table S1). The green fluorescent emission of Co-3 to Co-6 enabled their detection and visualization within cells. Analysis of confocal microscope merged images and orthogonal projections of NSCLC cells, expressing high (H1975 cells) or low (H1299 cells) levels of PD-L1, showed that Co-3 to Co-6, at their biologically required IC_50_ doses, were internalized after 3 h (Fig. S14). Among all, Co-3 exhibited the greatest intracellular accumulation, especially in H1975 cells (PD-L1 high-expressing), and the highest colocalization with PD-L1 (Fig. 1G and Fig. S14). Flow cytometry analysis further confirmed the microscopy findings, supporting the highest emission of Co-3 in PD-L1-expressing cells (Fig. S15). Remarkably, incubation with a nontoxic dose (1 μmol/l) of Co-3 of NSCLC cells co-cultured with HLA-matched PBMCs led to a PD-L1-dependent increase in apoptotic induction and IFN-γ release by NSCLC cells and PBMCs, respectively (Fig. 1H and Fig. S16), as compared to untreated cells (*P* < 0.001), cells treated with Ir-2 (*P* < 0.05 and *P* < 0.001, respectively) or Tr-10 alone (*P* < 0.05 and *P* < 0.001, respectively) and to conjugates Co-4, Co-5, and Co-6 (*P* < 0.001). Moreover, CEllular Thermal Shift Assay (CETSA), nuclear magnetic resonance titration experiments, and molecular docking demonstrated a direct binding of Co-3 to PD-L1, either in its isolated form or when expressed on the cell membrane (Figs. S17 to S19). Furthermore, in PD-L1 knockout cell clones (H1975^*PD-L1 KO-1*^ and H1975^*PD-L1 KO-2*^), generated via clustered regularly interspaced short palindromic repeats/CRISPR-associated protein 9 (CRISPR/Cas9) genome editing (Fig. S20), Co-3 did not accumulate intracellularly (Fig. 1I).

Evaluation of apoptosis and IFN-γ release in H1975^*PD-L1 KO-1*^ cells treated with Co-3 and co-cultured with stimulated PBMCs confirmed the PD-L1-dependent uptake of Co-3. Specifically, Co-3 did not affect apoptosis induction (Fig. 1J) or IFN-γ release (Fig. 1K) in H1975^*PD-L1 KO-1*^ cells as compared to H1975*^Ctrl^* cells transfected with a nontargeting single guide RNA control. Importantly, Co-3 induced a PD-L1-dependent DAMP response in H1975*^Ctrl^* cells, as evidenced by increased CRT exposure (Fig. 1L and Fig. S21A), ATP release (Fig. 1M), and HMGB1 release (Fig. 1N and Fig. S21B), as compared to untreated cells. In contrast, no DAMP induction was observed in H1975^*PD-L1 KO*^ cells, where all markers remained at baseline levels, confirming the PD-L1-dependent nature of this response.

Of note, Co-3 treatment of noncancer cells (HaCaT), co-cultured with HLA-matched PBMCs, resulted in only a marginal and nonsignificant effect on apoptosis induction as compared to untreated cells (Fig. S22). Altogether, these data strongly support the selective PD-L1-mediated internalization of Co-3 due to its conjugation with the PD-L1 inhibitor Tr-10, as well as the ability of Co-3 in retaining both the functional properties of PD-L1 blocking and Ir-2-mediated enhanced susceptibility of cancer cells to immune cells.

In conclusion, we demonstrated the following: (a) a synergistic interaction between a novel Ir(III) complex and a small anti-PD-L1 molecule in NSCLC, (b) that covalent integration of the 2 active agents into a single chemical entity enhances synergy and allows for an over 10-fold reduction in the required PD-L1 inhibitor dose, and (c) that the enhanced release of DAMPs and ICD induction by the Ir(III) complex could underlie the increased immune-mediated antitumor activity of anti-PD-L1. Collectively, these results support the development of next-generation NSCLC immunotherapies by providing a strategy capable of overcoming resistance to anti-PD-1/PD-L1 and potentially improving clinical benefit. Unlike existing PD-L1-targeting platforms, including anti-PD-L1 antibody–drug conjugates and antibody-based immunoconjugates, Co-3 uniquely integrates PD-L1-directed targeting with the intrinsic immunomodulatory activity of its Ir(III) core, a dual mechanism absent in current systems. Nevertheless, future in vivo studies will be crucial to define the therapeutic potential of iridium complexes, including their ability to prevent resistance to anti-PD-1/PD-L1 therapy. While these findings provide robust evidence of enhanced antitumor immune responses in vitro, further validation in immunocompetent in vivo models will be necessary to fully characterize their impact on T-cell activation, immune infiltration, and response to immune checkpoint blockade.

Although in vitro assays cannot capture pharmacokinetics or microenvironmental complexity, Figs. S23 to S25 show that the selected doses of Co-3 are nontoxic in PD-L1-expressing myeloid populations (monocytes and dendritic cells), supporting the feasibility of this approach. Notably, functional data from co-culture systems further indicate that myeloid cells contribute to Co-3-mediated immune activation, as evidenced by enhanced IFN-γ release in PBMC-based co-cultures. However, the significant increase in IFN-γ also observed in CD14^−^/CD11b^−^ peripheral-blood-lymphocyte-based co-cultures suggests that Co-3 can directly promote T-cell effector function, regardless of monocyte activation (Fig. S26). Therefore, these findings support a model in which Co-3 enhances antitumor immunity through both myeloid-dependent and myeloid-independent mechanisms. Nanotechnology-based delivery strategies may further enhance tumor targeting by improving drug stability, circulation time, and selective accumulation within the tumor microenvironment, thereby increasing therapeutic efficacy while minimizing systemic toxicity.

## Ethical Approval

All experimental protocols were approved by the relevant institutional ethics committee. The PBMCs were isolated from healthy donors after obtaining informed consent, according to the approval of the local ethics committee (prot./SCCE no. 85.275) and in accordance with the Declaration of Helsinki and its amendments.

## Data Availability

No datasets subject to mandatory public deposition were generated or analyzed in this study. All data supporting the conclusions are included in the published article and its Supplementary Materials.

## Supplementary Materials

Supplementary 1 Materials and Methods Tables S1 and S2 Figs. S1 to S44 References [8–10]
