# Supplementary material for: Enhancing Non-small Cell Lung Cancer Susceptibility to Anti-PD-1/PD-L1 Therapy through PD-L1 Ligand–Ir(III) Complex Conjugates
Source: Cancer Commun (Lond). 2026 May 13;46:0029. doi: 10.34133/cancomm.0029 (PMC13168757; doi:10.34133/cancomm.0029)
Supplement: Supplementary 1 — Materials and Methods Tables S1 and S2 Figs. S1 to S44 References [8–10] [file cancomm.0029.f1.docx]

**Supplementary material for**

**Enhancing non-small cell lung cancer susceptibility to anti-PD-1/PD-L1 therapy through PD-L1 ligand-Ir(III) complex conjugates**

Valentina Pagliara^1, †^, Giulia Assoni ^2, 3, †^, Giovanna Polcaro^1^, Alberto Bossi^4^, Marta Penconi^4^, Luigi Liguori^1,5^, Pierfausto Seneci^3^, Francesco Saverio Di Leva^6^, Vincenzo Maria D’Amore^6^, Diego Brancaccio^6^, Federica Santoro^6^, Cristina R. Ferrone^5^, Elena Ciaglia^1^, Annibale Alessandro Puca^1^, Stefano Pepe^1^, Luciana Marinelli^6, *^, Daniela Arosio^4, *^, Francesco Sabbatino^1, *^

^1^Department of Medicine, Surgery and Dentistry, University of Salerno, Baronissi 84081, Italy

^2^Department of Cellular, Computational and Integrative Biology (CIBIO), University of Trento, Povo I-38123 Trento, Italy

^3^Department of Chemistry, University of Milan, Milan 20133, Italy

^4^Istituto di Scienze e Tecnologie Chimiche “Giulio Natta” (SCITEC), Consiglio Nazionale delle Ricerche (CNR), Milan 20133, Italy

^5^Department of Surgery, Cedars-Sinai Medical Center, Los Angeles 90048, California, USA

^6^Department of Pharmacy, University of Naples “Federico II”, Naples 80131, Italy

^*^Correspondence to:

1. Francesco Sabbatino, Department of Medicine, Surgery and Dentistry, University of Salerno, Baronissi 84081, SA, Italy; E-mail: fsabbatino@unisa.it

2. Daniela Arosio, Istituto di Scienze e Tecnologie Chimiche “Giulio Natta” (SCITEC), Consiglio Nazionale delle Ricerche (CNR), Milan 20133, MI, Italy; E-mail: daniela.arosio@cnr.it

3. Luciana Marinelli, Department of Pharmacy, University of Naples “Federico II”, Naples 80131, NA, Italy; E-mail: lmarinel@unina.it

^†^These authors equally contributed to this work.

**Materials and Methods**

***Design and synthesis of Programmed cell Dead receptor Ligand-1 (PD-L1) ligand-Ir(III) complex conjugates***

A small set of conjugates between our anti-PD-L1 antibody, Tr-10 (**Figure 1**) and a bis(phenyl-pyridine)iridium(III) complex (Ir-2) (**Figure 1**) was designed and synthesized. Based on a model for the interaction of Tr-10 within PD-L1 [1], two positions on Tr-10 were selected for introducing a spacer ending with a bidentate 2-pyridyl-triazole ligand for Ir(III) complex formation. Namely, Ir(III)-complexes were conjugated either on the polar chain of a disubstituted triazine (Co-3 and Co-4; **Figure 1**), or as a third substituent in a trisubstituted triazine (Co-5 and Co-6; **Figure 1**), with both “short” (two carbon atoms) and “long” (three to five carbon atoms) linkers introduced on each position to determine the optimal distance between the PD-L1 ligand and the Ir(III) center.

The synthesis of disubstituted triazine Ir(III) complexes Co-3 and Co-4 and trisubstituted triazine Ir(III) complexes Co-5 and Co-6 was accomplished through the preparation of 2-pyridyl-triazole intermediates obtained according to a precedented, slightly modified procedure, followed by complexation with commercial dimeric [Ir(ppy)_2_Cl]_2_ [2]. A complete description of the synthetic procedures, and of the analytical characterizations of such complexes is reported below (see **Synthetic procedure for PD-L1 ligand-Ir(III) complex conjugates Co-3 to Co-6**).

***Photophysical properties***

To assess their suitability as phosphorescent probes for cellular detection, the photophysical properties of Ir(III) conjugate compounds Co-3 to Co-6, such as absorption, emission, and quantum yields (Φ) were examined both at 298 K (in degassed Dichloromethane [DCM]) and at 77 K (in 2-methyl-Tetrahydofurane [2Me-THF]). The unconjugated parent Ir-2 was also characterized. Their collected photophysical properties are summarized in **Supplementary Table S2**.

Overall, absorption and emission spectra indicated that the optical features of the studied complexes are determined by the Ir(ppy)_2_tazpy moiety. Co-3 to Co-6 displayed an absorption spectrum characterized by an intense UV band below 330 nm, due to π-π* transition of the organic frameworks, while transitions of metal-to-ligand-charge-transfer (MLCT) character were observed between 330-430 nm; further small signals between 430 and 480 nm (with ε < 600 cm^-1^M^-1^) were ascribed to the direct population of the triplet state. Bright bluish-green emission is shown by the complexes, with a maximum at 478 nm. Impressively high quantum yield (QY, up to 0.63, with a lifetime of 2.69 μs for Co-5) made these complexes sparkling emissive in comparison with parent Ir-2 (QY = 0.23). Upon cooling at 77 K in a rigid 2-methyl-tetrahydrofuran matrix, their phosphorescence emission showed only a slight blueshift with a 470 nm maximum, while gaining a significant structuration of the spectrum. These characteristics made synthesized Ir(III) complexes suitable as phosphorescent probes for cellular mechanistic studies.

***Cell Culture and Treatment***

Human NSCLC cell lines (H1975, H1299, HCC827, H1703), normal immortalized human keratinocytes (HaCaT) and bronchial epithelium (Beas-2B) cell lines were obtained from Deutsche Sammlung von Mikroorganismen und Zellkulturen (DSMZ, ACC 118) and American Type Culture Collection (ATCC, CRL-9609). Cancer cells and HaCaT were cultured in RPMI 1640 medium (Euroclone ECB9006) supplemented with 10% fetal bovine serum (FBS; ECS0180L, Euroclone) and 1% penicillin-streptomycin (ECB3001D, Euroclone). Beas-2B cells were cultured in DMEM medium (ECM0728L, Euroclone) supplemented with 5% fetal bovine serum (FBS, ECS0180L, Euroclone) and 1% penicillin-streptomycin (ECB3001D, Euroclone). Cells were cultured at 37°C in a humidified incubator containing 5% CO_2_. NSCLC cell lines (H1975 and H1299) exhibit different PD-L1 expression levels under basal conditions and show a marked upregulation of PD-L1 following IFNγ stimulation (Figure S2) [3]. PBMCs were extracted from healthy donors’ whole blood by Ficoll density gradient (Histopaque®, 1077, 10771, Sigma–Aldrich) as reported [4]. After separation, the PBMCs were washed and collected in RPMI 1640 medium (11875093, Gibco^®^, Thermo Fisher Scientific) supplemented with 10% (v/v) fetal bovine serum (FBS, A5670201, Gibco^®^, Thermo Fisher Scientific), 1% (v/v) penicillin–streptomycin (15140122, Gibco^®^, Thermo Fisher Scientific). PBMCs were activated for 1 h before co-culturing with an anti-CD3 (1 μg/mL) and anti-CD28 (1 μg/mL) T Cell Trans Act (T-Act, 130-111-160, Miltenyi Biotec). Treatments were performed using 1 μmol/L of Ir(III) complexes Ir-2 and Co-3 to Co-6 and 10 μmol/L PD-L1 inhibitor Tr-10 [1] for 24 h and 48 h. For uptake and Cellular Thermal Shift Assay (CETSA) experiments, treatments were performed using the IC_50_ of conjugates Co-3 to Co-6 (**Supplementary Figures** **S13-16**). Dimethyl sulfoxide (DMSO) (vehicle of all compounds) concentration was maintained at 0.01% in all wells. Paclitaxel (PCTX; 50 nmol/L) was used as a positive control for the induction of DAMPs emission (ATP and HMGB1 release and CRT exposure on cell membrane) for 24 or 48 h.

***Cytotoxicity Assays***

Cell lines were seeded at a density of 1 × 10^4^ per well in 96-well plates and were exposed to Tr-10 and Ir-2 using a dose-response scheme of 0, 1, 5, 10, and 100 μmol/L. Following 24 h and 48 h of treatments, cells were isolated with phosphate-buffered saline (PBS) washing. Cell viability was determined by cell counting kit-8 (CCK-8) assay (CK04-13, Dojindo Laboratories) according to the manufacturer’s protocol. The absorbance at 450 nm was determined by a Sunrise microplate reader (TECAN). Data are expressed as percentages of cell viability of treated cells as compared with untreated cells, set as 100%. All experiments were performed in three independent experiments, each of which was performed in triplicate.

***Synergy analysis***

H1975 and H1299 cell lines were seeded at a density of 1 × 10⁴ cells/well in 96-well plates and exposed to Tr-10 and Ir-2 either as single agents or in combination, using a dose-response scheme of 0, 1, 5, 10, and 100 μmol/L for 48 h. All concentrations of Tr-10 were combined with all concentrations of Ir-2. After treatment, cancer cells were washed and cell viability was assessed using the Cell Counting Kit-8 (CCK-8) assay, following the manufacturer’s instructions. Absorbance at 450 nm was measured using a Sunrise microplate reader (TECAN). Viability values were expressed as percentages relative to untreated cells (set as 100%). All experiments were conducted in three independent biological replicates, each performed in technical triplicate. Viability values in percentage were used to generate the full dose-response matrix and the heatmap values, which indicate the percentage of cell inhibition used for synergy analysis. Drug-drug interaction analyses were performed using SynergyFinder 3.0 (<https://synergyfinder.fimm.fi/>). Synergy was quantified using the Zero Interaction Potency (ZIP) model, which evaluates the deviation of the observed combination response from the expected non-interactive effect across the dose–response matrix. SynergyFinder generated a summary Synergy Score (SS) for each interaction matrix. In our study, SS values ≤ −5 were considered indicative of antagonism, SS values ≥ +5 were considered indicative of synergy, and scores between −5 and +5 were interpreted as noise or variation consistent with experimental variability and non-interactive (additive or additive-like) effects, in line with previously published practices [5]. To highlight interactions occurring within pharmacologically relevant and non-toxic dose ranges, the “weighted synergy by concentrations” post-hoc module was applied. The resulting 2D and 3D ZIP synergy landscapes were used to identify the most synergistic concentration window for subsequent validation assays.

***Genetic Ablation of the PD-L1 Gene and Isolation of Knockout PD-L1 H1975 cells***

H1975 cells were seeded at a density of 2 × 10^4^ per well in 24-well plates. PD-L1 knock out was performed using short guide RNAs (sgRNAs) against the PD-L1 gene; sgRNA 1 (5’- TGGCTGCACTAATTGTCTAT -3’) and sgRNA 2 (5’- ACCCCAAGGCCGAAGTCATC-3’).

As a control, an sgRNA with no homologies to the human genome (non-target control) was used [6].

Ribonucleoprotein (RNP) complexes composed of two different sgRNAs (12 nmol/L; Integrated DNA Technologies) and Cas9 Nuclease V3 enzyme (6 nmol/L; Integrated DNA Technologies) were delivered into H1975 cells using TransIT-X2^®^ Dynamic Delivery System (1 µL/well, MIR 6004, Mirus-Bio) for 72 h. The transfection efficiency was assessed by measuring TEX 615 Transfection Control siRNA expression (51-01-20-22, Integrated DNA Technologies) using a confocal microscope. Stable clones were generated by collecting transfected H1975 cells and isolating them by limiting dilution. Generated- single clones were analyzed by Western blotting (**Supplementary Figure S20**) to verify gene inactivation. Clones 14 and 22, named H1975 *^PD-L1 KO-1^* and H1975 *^PD-L1 KO-2,^* were chosen for further characterization.

***Co-culture of normal and cancer cell lines with*** ***HLA-matched PBMCs***

H1975, H1299, HCC827, H1703, HaCaT and Beas-2B cell lines were seeded at a density of 2 × 10^4^ per well in 24-well plates. Peripheral blood mononuclear cells (PBMCs) were isolated from the peripheral blood of healthy donors by Ficoll density gradient centrifugation. HLA class I (human leukocyte antigen class I; HLA-A, -B, -C) genotyping of NSCLC cell lines and PBMCs from healthy donors was performed at low resolution at the Transplant Hematology Unit of “San Giovanni di Dio e Ruggi d’Aragona” University Hospital, following European Federation of Immunogenetics (EFI) standards [7]. Genomic DNA was extracted using the QIAamp DNA Mini Kit (51306, Qiagen), quantified, and HLA typing was carried out by PCR using sequence-specific primers (SSP; Protrans), with amplification products analyzed by agarose gel electrophoresis and interpreted using Helmberg SCORE software. Cell line HLA profiles were further validated using the TRON Cell Line Portal. Following a 24 h incubation at 37°C in a 5% CO_2_ atmosphere, HLA-matched PBMCs were activated for 1 h using anti-CD3 (1 μg/mL) and anti-CD28 (1 μg/mL) T Cell Trans Act (T-Act). Activated PBMCs were then added to cancer cells (5:1 ratio) and incubated with compounds for 24 h and 48 h.

***Isolation of PBLs and monocyte cells from PBMCs and generation of monocyte-derived dendritic cells and macrophages***

PBMCs from a healthy donor or NSCLC patient after obtaining informed consent, according to the approval of the local ethics committee (prot./SCCE no. 85.275), and in accordance with the Declaration of Helsinki and its amendments, were used to isolate CD14^+^ monocytes or CD11b^+^ myeloid populations, including monocytes, macrophages, and subsets of myeloid-derived suppressor cells (MDSCs) by an immunomagnetic procedure (CD14 and CD11b micro beads, 130-097-052 and 130-049-601, respectively, Miltenyi Biotec) while the unlabeled flow-through fraction was collected as peripheral blood lymphocytes (PBLs) for downstream assays. The immature dendritic cells were then obtained by culturing 10^6^ monocytes CD14^+^/mL in RPMI 1640 medium supplemented with 50 ng/mL granulocyte macrophage colony-stimulating factor (GM-CSF, 130-095-373, Miltenyi Biotec) and 1,000 U/mL IL-4 (**130-093-921, Miltenyi Biotec)** for seven days [8]. The macrophages were obtained by culturing 10^6^ monocytes CD14^+^/ml in ImmunoCult-SF Macrophage Differentiation Medium (10961, Stemcell Technologies) for seven days. During the third day of culture, 50% of the medium was replaced with fresh medium.

***Cytofluorimetric Analysis***

Cell lines were seeded at a density of 2 × 10^4^ per well in 24-well plates. Following 24 h and/or 48 h incubation at 37°C in a 5% CO_2_ atmosphere, cells were harvested and cell surface stained with mAbs against HLA-A, HLA-AB, HLA-ABC, β2-microglobulin light chain, HLA-DR-DQ-DP, calnexin and calreticulin [9]. Cell staining was detected by R-phycoerythrin (PE)-conjugated F(ab’)2 fragment goat anti-mouse IgG (1:100, 115-116-146, Jackson Immuno Research). Mouse IgG (1:100, sc-2025, Santa Cruz Biotechnology) was used as a specificity control of mouse antibodies. Cell staining of cell surface PD-L1 was detected with an Allophycocyanin (APC) anti-PD-L1 IgG2b mouse mAb (1:100, 558065, BD Pharmingen). Mouse APC-conjugated IgG2b (1:100, 400611, BioLegend) was used as a specificity control. To identify and distinguish myeloid cell populations, cells were stained with purified mouse monoclonal antibodies against CD14 (1:100, 555392, BD Biosciences), CD1a (1:50, 555805, BD Biosciences), and CD68 (1:100, 556059, BD Biosciences), followed by incubation with an APC-Cy7-conjugated goat anti-mouse IgG secondary antibody (1:200, 557873, BD Biosciences).

Stained cells were analyzed using a FACSVerse flow cytometer (BD Biosciences). To perform cell uptake of Ir(III) complex conjugates Co-3 to Co-6, H1299 and H1975 cells were treated with IC_50_ doses for each compound. After 3 h, cells were collected and according to emission spectra (**Supplementary Figure S1**), FITC channel (green) was used for detection of wavelength emission, using a FACSVerse flow cytometer. Data, expressed as mean fluorescence intensity (MFI), are representative of the results obtained in three independent experiments.

***MitoSOX-based flow cytometric assay***

Cell lines were seeded at a density of 2 × 10^4^ per well in 24-well plates. MitoSOX-based flow cytometric assay (M36008, Thermo Fisher) was used to detect mitochondrial ROS [10]. After treatments, cells were harvested, and 1 μmol/L MitoSOX was added to the cells for 30 min at 37°C. MitoSOX-derived MFI was read at 610 nm using a FACSVerse flow cytometer. Data, expressed as MFI fold change with respect to untreated cells, reflecting the levels of mitochondrial total ROS, are representative of the results obtained in three independent experiments.

***IFN-γ ELISA***

Cell lines were seeded at a density of 2 × 10^4^ per well in 24-well plates. Following a 24 h and 48 h co-culture of cancer cells with HLA-matched PBMCs, the medium was harvested, and IFN-γ levels were analyzed using the ELISA Max Deluxe Set Human IFN-γ (430104, BioLegend) assay according to the manufacturer’s protocol. The absorbance at 450 nm was determined by a Sunrise microplate reader (TECAN). All experiments were performed in three independent experiments.

***Annexin V-FITC/PI Assay***

Cell lines were seeded at a density of 2 × 10^4^ per well in 24-well plates. Following a 24 h and 48 h co-culture of cancer cells with HLA-matched PBMCs, cells were washed with Annexin-V Binding Buffer (51-66121E, BD Pharmigen) and were resuspended in 100 μL Annexin-V Binding Buffer containing 2.5 μL of Annexin-V/FITC and 2.5 μL of propidium iodide (PI) (FITC Annexin V detection kit, 556547, BD Pharmigen), for 15 min at room temperature (RT). After incubation, 400 μL of Annexin-V Binding Buffer was added, and samples were analyzed using the FACSVerse Flow Cytometer. Data were expressed as a mean fraction of annexin V-positive cells (early and late apoptosis). All experiments were performed in three independent experiments.

***ATP assay***

Cells were seeded at a density of 2 × 10^4^ per well in 24-well plates. To elicit immunogenic cell death (ICD), cells were treated with 50 nmol/L of Paclitaxel (PCTX, T7402, Sigma-Aldrich), a well-known ICD inducer, for 24 h. Following 24 h of incubation at 37°C in a 5% CO_2_ atmosphere, the medium was harvested, and extracellular ATP levels were assessed using the ATP Assay Kit (ab83355, Abcam), according to the manufacturer’s protocol. The absorbance at 570 nm was determined by a Sunrise microplate reader (TECAN). All experiments were performed in three independent experiments, each of which was performed in triplicate.

***Western Blotting Analysis***

Cells were seeded at a density of 2 × 10^4^ per well in 24-well plates. Following 24 h and 48 h treatments, whole-cell lysates were prepared as previously described [11]. Additionally, the cell supernatant was concentrated approximately 20-fold by centrifugation at 5000 × g using an Ultra-4 PLGC Ultracell-PL Membrane (10 kDa cut-off; UFC 801024, Merck Millipore) at 4 °C [12]. For protein analysis on the conditioned medium, appropriate volumes of concentrated samples corresponding to 100 μg total proteins [13] were loaded onto 10% or 15% SDS–PAGE and detected by Western blotting. After electrophoresis, proteins were transferred to a nitrocellulose membrane and then incubated with a specific primary antibody. The following antibodies were used: rabbit mAb raised against GRP78/Bip (1: 1000, 3177, Cell Signaling), rabbit polyclonal antibodies raised against HMGB1 (1: 1000, 6893, Cell Signaling), and rabbit mAb raised against PD-L1 (1:1000, 13684, Cell Signaling). After incubation with the appropriate peroxidase-linked secondary antibody (1:3000, 31460, Pierce), detection was achieved using the Enhanced Chemiluminescence (ECL) kit (K-12045-D50, Advansta). Densitometry analysis was performed using the free image-processing software ImageJ version 1.47 (<http://rsb.info.nih.gov/ij/>, accessed on 10 November 2022).

***CEllular Thermal Shift Assay (CETSA)***

Cells were seeded at a density of 2 × 10^6^ per well in 100 mm plates and incubated with Co-3 IC_50_ concentration for 2 h. After the treatments, CETSA was performed to identify target engagement between ligands and their protein targets [14]. Briefly, cells were collected, and the samples were divided into 6 aliquots, each of them then subjected to a 5 min incubation at a specific temperature, in the range from 45°C to 60°C. Samples were then lysed and centrifuged to separate the soluble proteins from the aggregated and precipitated ones. The amount of PD-L1 soluble protein target was evaluated by Western blotting. PD-L1 levels, normalized to GAPDH (1:1000, 2118, Cell Signaling) levels, at 45°C were set at 100%.

***Confocal Microscopy Analysis***

Cells were seeded at a density of 2 × 10^5^ per well in 6-well plates on cover glass. To perform confocal analysis of cell uptake of Ir(III) complex conjugates Co-3 to Co-6, cells were treated with IC_50_ doses of compounds. After 3 h, cells were stained with antibodies anti-PD-L1 (1:100, 13684, Cell Signaling) for 1h, Cy5-linked secondary antibody (1:50, 111-175-144, Jackson Immuno-Research) for 1 h and 4’,6-diamidino-2-phenylindole (DAPI) (1:1000, D9542, Sigma-Aldrich) was used to visualize the nuclei. After washing, coverslips were mounted with a Vecta-mount medium (H-5000, Vector Laboratories). For mitochondria staining, cells were incubated for 30 min at 37°C with 200 nmol/L Mitotracker Red CMXRos (M7512, Invitrogen-Molecular Probes) before fixing in cold acetone for 5 min on ice. Images were acquired with a laser scanning confocal microscope TCS SP5 (Leica MicroSystems) equipped with a plan Apo 63X, NA 1.4 oil immersion objective lens. Pictures were processed using LAS-AF Software (Leica MicroSystems) to reconstruct the *x*-axis projection using stack images [15].

***Molecular basis of the interaction of the Ir(III) complex conjugate Co-3 and PD-L1: NMR and molecular docking experiments***

Small molecule PD-L1 inhibitors bearing a biphenyl core, including parent compound Tr-10, bind at the protein dimer interface [16], by inserting their biaryl moiety into a deep hydrophobic channel. However, given the increased steric bulk of conjugate Co-3, resulting from the attachment of a biphenyl-pyridine-Ir(III) complex to Tr-10, it is not immediately evident whether it adopts the same binding modality. To elucidate the molecular basis of its interaction with PD-L1, we employed a combined approach involving Nuclear Magnetic Resonance (NMR) titration experiments and molecular docking. In the ¹⁵N NMR titration experiments, Proton–Nitrogen Heteronuclear Single Quantum Coherence (^1^H-^15^N HSQC) spectra were recorded upon addition of increasing concentrations of Co-3 to a fixed concentration of ^15^N human PD-L1, monitoring chemical shift perturbations and changes in protein signal intensity. As shown in **Supplementary Figure S17**, the most significantly perturbed residues are located near the dimer interface, supporting a binding site analogous to that of small-molecule PD-L1 inhibitors. These experimental findings guided our docking simulations, which were focused on the hydrophobic pocket at the PD-L1 dimer interface. Remarkably, despite its increased molecular size, all predicted binding poses of Co-3 positioned its biaryl core deep within the interfacial channel (**Supplementary Figures S17-S18**), closely mirroring the orientation of Tr-10 [1], and structurally related inhibitors. Notably, the Ir(III)-containing complex fragment is oriented outward from the interfacial pocket, extending toward the solvent-exposed region. In the lowest-energy configuration, this moiety is further stabilized by cation–π interactions with the basic residues K124_A_ and R125_A_. The high degree of docking pose convergence, the favorable docking score (–11.439), and the extensive network of predicted non-covalent interactions collectively provide a compelling mechanistic rationale for the potent biological activity of compound Co-3 observed in both *in vitro* and cellular assays.

***Molecular Docking Methods.***

The 3D structure of conjugate Co-3 was constructed using the Maestro Build Panel as implemented in the Schrödinger package (Schrödinger Release 2025-2). Tautomeric and protomeric states relevant at physiological pH (7.4 ± 1.5) were predicted using the Epik [17, 18]module integrated within the LigPrep tool (Schrödinger Release 2025-2). Default parameters for iridium were assigned by specifying pharmacophore type 1 and force field type 2 in the ptype.def file. The crystal structure of homodimeric PD-L1 in complex with compound BMS-202 (PDB ID: 5J89) [16] was chosen as the receptor model based on the structural similarity between the co-crystallized ligand and conjugate Co-3, and to enable direct comparison with previous studies involving the parent compound Tr-10 [6, 19]. The receptor was prepared using the Protein Preparation Wizard [20] in the Maestro Suite (Schrödinger Release 2025-2), which included the addition of missing hydrogen atoms, optimization of the hydrogen bonding network, assignment of appropriate side-chain ionization and tautomeric states, and removal of water molecules not forming at least two hydrogen bonds with protein or ligand atoms. The docking grid (inner box = 13Å × 13Å × 13Å; outer box = 35 Å × 35Å × 35Å) was centered on the binding site of the co-crystallized ligand using the Grid Generation tool in Glide (version 10.2) [21]. Molecular docking of conjugate Co-3 was performed using Glide in Standard Precision (SP) mode, employing the OPLS4 force field to model protein–ligand interactions with high accuracy.

***Nuclear Magnetic Resonance Experiments***

In the ^15^N NMR titration experiments, 2D ^1^H–^15^N HSQC spectra were recorded at 298 K on a Bruker AVANCE NEO 600 MHz spectrometer equipped with a cryogenically cooled probe. Increasing concentrations of compound Co-3 (12.5 to 100 μM, dissolved in DMSO-d₆) were added to a 50 μmol/L solution of ^15^N-labeled PD-L1 in Tris buffer (10 mmol/L Tris, pH 8, 20 mmol/L NaCl, 0.1% NaN₃, protease inhibitors, 10% ²H₂O). The binding interaction was assessed by monitoring chemical shift perturbations and changes in cross-peak intensity ratios (I/I₀) in the HSQC spectra. All NMR spectra were processed using TopSpin 4.4.0 (Bruker BioSpin GmbH) and analyzed with the Computer Aided Resonance Assignment software (Keller, 2003, the CARA/Lua programmers manual). Protein backbone resonance assignments were based on data deposited in the Biological Magnetic Resonance Data Bank (BMRB accession code: 51169).

***Statistical analysis***

Statistical analyses were conducted using GraphPad Prism v6.0 (GraphPad Software) using One-way analysis of Variance (ANOVA), followed by the Bonferroni test. Each value represents the mean ± SD of at least three independent experiments (* *P* < 0.05, ** *P* < 0.01, *** *P* < 0.001).

**References**

1. Russomanno P, Assoni G, Amato J, D'Amore VM, Scaglia R, Brancaccio D, et al. Interfering with the Tumor-Immune Interface: Making Way for Triazine-Based Small Molecules as Novel PD-L1 Inhibitors. J Med Chem. 2021;64(21):16020–45.

2. Lo KK-W, Li SP-Y, Zhang KY. Development of luminescent iridium(iii) polypyridine complexes as chemical and biological probes. New Journal of Chemistry. 2011;35(2):265–87.

3. Miao J, Hsu PC, Yang YL, Xu Z, Dai Y, Wang Y, et al. YAP regulates PD-L1 expression in human NSCLC cells. Oncotarget. 2017;8(70):114576–87.

4. Puca AA, Lopardo V, Montella F, Di Pietro P, Cesselli D, Rolle IG, et al. The Longevity-Associated Variant of BPIFB4 Reduces Senescence in Glioma Cells and in Patients' Lymphocytes Favoring Chemotherapy Efficacy. Cells. 2022;11(2).

5. Malyutina A, Majumder MM, Wang W, Pessia A, Heckman CA, Tang J. Drug combination sensitivity scoring facilitates the discovery of synergistic and efficacious drug combinations in cancer. PLoS Comput Biol. 2019;15(5):e1006752.

6. Döring M, Brux M, Paszkowski-Rogacz M, Guillem-Gloria PM, Buchholz F, Pisabarro MT, et al. Nucleolar protein TAAP1/C22orf46 confers pro-survival signaling in non-small cell lung cancer. Life Sci Alliance. 2024;7(4).

7. Polcaro G, Liguori L, Manzo V, Chianese A, Donadio G, Caputo A, et al. rs822336 binding to C/EBPβ and NFIC modulates induction of PD-L1 expression and predicts anti-PD-1/PD-L1 therapy in advanced NSCLC. Mol Cancer. 2024;23(1):63.

8. Monfrecola G, Lembo S, Cantelli M, Ciaglia E, Scarpato L, Fabbrocini G, et al. The effect of visible blue light on the differentiation of dendritic cells in vitro. Biochimie. 2014;101:252–5.

9. Sabbatino F, Wang Y, Scognamiglio G, Favoino E, Feldman SA, Villani V, et al. Antitumor Activity of BRAF Inhibitor and IFNα Combination in BRAF-Mutant Melanoma. J Natl Cancer Inst. 2016;108(7).

10. Kauffman ME, Kauffman MK, Traore K, Zhu H, Trush MA, Jia Z, et al. MitoSOX-Based Flow Cytometry for Detecting Mitochondrial ROS. React Oxyg Species (Apex). 2016;2(5):361–70.

11. Pagliara V, Parafati M, Adornetto A, White MC, Masullo M, Grimaldi M, et al. Dibutyryl cAMP- or Interleukin-6-induced astrocytic differentiation enhances mannose binding lectin (MBL)-associated serine protease (MASP)-1/3 expression in C6 glioma cells. Archives of Biochemistry and Biophysics. 2018;653:39–49.

12. Pagliara V, Nasso R, Di Donato P, Finore I, Poli A, Masullo M, et al. Lemon Peel Polyphenol Extract Reduces Interleukin-6-Induced Cell Migration, Invasiveness, and Matrix Metalloproteinase-9/2 Expression in Human Gastric Adenocarcinoma MKN-28 and AGS Cell Lines. Biomolecules. 2019;9(12).

13. Pagliara V, Adornetto A, Mammì M, Masullo M, Sarnataro D, Pietropaolo C, et al. Protease Nexin-1 affects the migration and invasion of C6 glioma cells through the regulation of urokinase Plasminogen Activator and Matrix Metalloproteinase-9/2. Biochimica et Biophysica Acta (BBA) - Molecular Cell Research. 2014;1843(11):2631–44.

14. Pagliara V, Donadio G, De Tommasi N, Amodio G, Remondelli P, Moltedo O, et al. Bioactive Ent-Kaurane Diterpenes Oridonin and Irudonin Prevent Cancer Cells Migration by Interacting with the Actin Cytoskeleton Controller Ezrin. Int J Mol Sci. 2020;21(19).

15. Pagliara V, Amodio G, Vestuto V, Franceschelli S, Russo NA, Cirillo V, et al. Myogenesis in C2C12 Cells Requires Phosphorylation of ATF6α by p38 MAPK. Biomedicines. 2023;11(5).

16. Zak KM, Grudnik P, Guzik K, Zieba BJ, Musielak B, Dömling A, et al. Structural basis for small molecule targeting of the programmed death ligand 1 (PD-L1). Oncotarget. 2016;7(21):30323–35.

17. Shelley JC, Cholleti A, Frye LL, Greenwood JR, Timlin MR, Uchimaya M. Epik: a software program for pK( a ) prediction and protonation state generation for drug-like molecules. J Comput Aided Mol Des. 2007;21(12):681–91.

18. Greenwood JR, Calkins D, Sullivan AP, Shelley JC. Towards the comprehensive, rapid, and accurate prediction of the favorable tautomeric states of drug-like molecules in aqueous solution. J Comput Aided Mol Des. 2010;24(6-7):591–604.

19. Donati G, D’Amore VM, Russomanno P, Cerofolini L, Amato J, Marzano S, et al. Theoretical and experimental studies on the interaction of biphenyl ligands with human and murine PD-L1: Up-to-date clues for drug design. Computational and Structural Biotechnology Journal. 2023;21:3355–68.

20. Sastry GM, Adzhigirey M, Day T, Annabhimoju R, Sherman W. Protein and ligand preparation: parameters, protocols, and influence on virtual screening enrichments. J Comput Aided Mol Des. 2013;27(3):221–34.

21. Friesner RA, Murphy RB, Repasky MP, Frye LL, Greenwood JR, Halgren TA, et al. Extra precision glide: docking and scoring incorporating a model of hydrophobic enclosure for protein-ligand complexes. J Med Chem. 2006;49(21):6177–96.

**Supplementary Tables**

| **Conjugates** | **IC_50_ (µmol/L)** | |
| --- | --- | --- |
|  | **H1975** | **H1299** |
| **Co-3** | 32.4 ± 0.68 | 27.7 ± 0.93 |
| **Co-4** | 37.5 ± 0.45 | 29.5 ± 0.57 |
| **Co-5** | 36.9 ± 4.21 | 21.4 ± 0.66 |
| **Co-6** | 31.7 ± 0.36 | 21.5 ± 0.62 |

**Supplementary Table S1.** **IC_50_ of Co-3-to Co-6 in H1299 and H1975 cells.**

H1975 and H1299 were seeded at a density of 1 × 10^4^ per well in 96-well plates and exposed to increasing concentrations of Co-3 to Co-6 (1-100 μmol/L). DMSO (vehicle) concentration was maintained at 0.01% in all wells. Following 48 h incubation, cell viability was determined by CCK-8 assay. IC50 values of Co-3 to Co-6 in H1299 and H1975 cells were calculated based on the cell viability data and are reported in the table. Abbreviations: DMSO: Dimethyl Sulfoxide, CCK-8: Cell Counting Kit-8, IC_50_: half maximal Inhibitory Concentration, Co-3: Conjugate-3, Co-4: Conjugate-4, Co-4: Conjugate-5, Co-6: Conjugate-6.

**Supplementary Table S2. Photophysical characterization of Ir(III) complexes Ir-2 and Co-3 to Co-6.**

| Compounds | λ _max. em_ (nm) | QY in N_2_ atm. | τ (µs) in N_2_ atm. | k_r_ [×10^5^ (s^-1^)]^a^ | k_nr_ [×10^5^ (s^-1^)]^b^ |
| --- | --- | --- | --- | --- | --- |
| Ir-2 | 477 (298 K) | 0.23 | 0.78 (298 K) | 2.95 | 9.87 |
| Co-3 | 478 (298 K)  470 (77 K) | 0.53 | 2.09 (298 K)  4.62 (77 K) | 2.54 | 2.25 |
| Co-4 | 478 (298 K)  470 (77 K) | 0.55 | 2.36 (298 K)  4.55 (77 K) | 2.33 | 1.91 |
| Co-5 | 478 (298 K)  470 (77 K) | 0.63 | 2.69 (298 K)  4.50 (77 K) | 2.34 | 1.38 |
| Co-6 | 478 (298 K)  470 (77 K) | 0.49 | 2.10 (298 K)  4.51 (77 K) | 2.33 | 2.43 |

^a^k_r_ = QY/τ; QY = Quantum Yield, τ: average lifetime of the excited state;

^b^k_nr_ = (1-QY) /τ; QY = Quantum Yield τ: average lifetime of the excited state

Abbreviations: em: emission; atm: atmosphere

**Supplementary Figures**


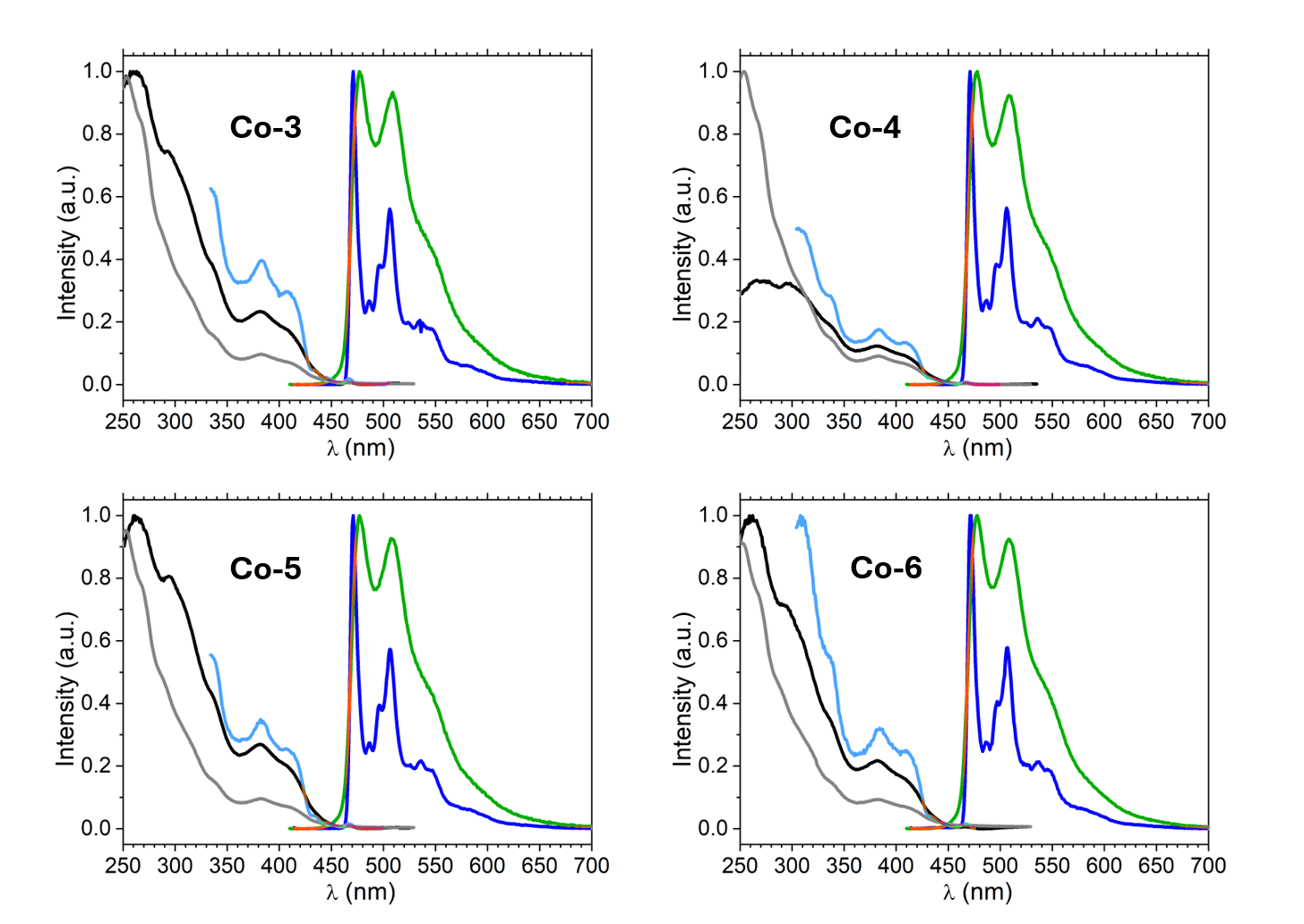


**Supplementary Figure S1. Emission and excitation spectra at room temperature (298 K) and at 77 K of conjugates Co-3 to Co-6.** Normalized spectra: *green line*, emission in dichloromethane at room temperature; *blue line*, emission in 2-methyltetrahydrofuran at 77 K; *black line*, excitation in dichloromethane at room temperature; *light-blue line*, excitation in 2-methyltetrahydrofuran at 77 K; *grey line*, absorption in dichloromethane at room temperature. Abbreviations: a.u.: absorbance units

**Supplementary Figure S2. IFN-γ–induced PD-L1 expression in NSCLC cells. (A)** Cells were seeded into 24-well plates at a density of 2 × 10^5^ cells per well and incubated with IFN-γ (100 ng/mL). Untreated cells were used as a control. Following a 24 h incubation at 37°C in a 5% CO_2_ atmosphere, expression levels of PD-L1 mRNA were evaluated by Real-Time-PCR. The levels of PD-L1, normalized to GAPDH and relative to H1975 and H1299 cells, are plotted and expressed as mean ± SD of the results obtained in three independent experiments (*** *P* < 0.001). For protein analysis cells were seeded into 6-well plates at a density of 2×10^6^ cells per well and incubated with IFN-γ (100 ng/mL). Untreated cells served as the control. Following a 48 h incubation at 37°C in a 5% CO_2_ atmosphere, cells were harvested. **(B)** Cell lysates were analyzed by Western Blotting with a PD-L1-specific mAb and normalized to GAPDH. Representative results are shown. **(C)** Representative flow cytometry analysis using an APC-conjugated anti-PD-L1 IgG2b mouse mAb. An APC-conjugated mouse IgG2b was used as an isotype control, demonstrating PD-L1 expression in H1975 and H1299 cells in basal and under IFN-γ-treated conditions. Overlays display isotype control (black) and PD-L1 staining (red for Ctrl and green for IFN-γ-treated samples). **(D)** Quantification of PD-L1 expression expressed as MFI relative to control cells. Data represent mean ± SD of three independent experiments (** *P* < 0.01; *** *P* < 0.001). Abbreviations: MFI, mean fluorescence intensity; IFN-γ, interferon gamma, SD: standard deviation, PD-L1: Programmed Death-Ligand, GAPDH: Glyceraldehyde-3-Phosphate Dehydrogenase; IgG2b: Immunoglobulin G subclass 2b; mAb: monoclonal antibody; RT-PCR: Real Time- Polymerase Chain Reaction; APC: Allophycocyanin.

**Supplementary Figure S3. Effects of exposure to Ir-2 and Tr-10 on cell viability of non-cancer and NSCLC cells.** **(A)** PBMCs, **(B)** HaCaT, **(C)** H1299 and **(D)** H1975 cells were seeded at a density of 1 × 10^4^ per well in 96-well plates and exposed to increasing concentrations of Tr-10 and Ir-2 (1–100 μmol/L). DMSO (vehicle) concentration was maintained at 0.01% in all wells. Following 24 h and 48 h incubation, cell viability was determined by CCK-8 assay. Data are expressed as percentages of cell viability ± SD for treated cells relative to untreated cells, set at 100%, obtained in three independent experiments; each experiment was performed in triplicate. Abbreviations: NSCLC: Non-Small Cell Lung Cancer; PBMC: Peripheral Blood Mononuclear Cells; DMSO: Dimethyl Sulfoxide; CCK-8: Cell Counting Kit-8; SD: Standard Deviation.

**Supplementary Figure S4. Synergy analysis of Tr-10 and Ir-2 in H1975 and H1299 cell lines.** H1975 and H1299 cells were seeded at a density of 1 × 10^4^ per well in 96-well plates and exposed to Tr-10 and Ir-2 either as single agents or in combination, using a dose-response scheme of 0, 1, 5, 10, and 100 μmol/L for 48 h. DMSO (vehicle) concentration was maintained at 0.01% in all wells. All concentrations of Tr-10 were combined with all concentrations of Ir-2, and the percentage of cell viability was used to generate the full dose-response matrix and the heatmap values (left panels), which indicate the percentage of cell inhibition used for synergy analysis. The two-dimensional (2D) ZIP synergy contour plot (center panels) and three-dimensional (3D) ZIP synergy surface (right panels) models were used to quantify the interaction between Ir-2 and Tr-10 across the full concentration matrix. Synergistic regions appear in red, whereas antagonistic areas are shown in green. The overall ZIP synergy score (δ) is indicated in the 3D surface plot. The green spot and guidelines highlight the synergy value for the combination of 10 μmol/L Tr-10 and 1 μmol/L Ir-2. Abbreviations: DMSO: Dimethyl Sulfoxide; ZIP: Zero Interaction Potency.

**Supplementary Figure S5. Enhancement of apoptosis induction and IFN-γ release by activated PBMCs co-cultured with NSCLC cells in the presence of Ir-2 and/or Tr-10.** **(A-D)** H1299 and H1975 cells were seeded at a density of 1 × 10^4^ per well in 96-well plates and 2 × 10^4^ per well in 24-well plates, co-cultured with activated PBMCs and treated with Tr-10 (10 μmol/L) and/or Ir-2 (1 μmol/L). PBMCs were stimulated with an anti-CD3 (1 μg/mL) and an anti-CD28 (1 μg/mL) T Cell TransAct (T Act). DMSO (vehicle of Ir-2 and Tr-10) concentration was maintained at 0.01% in all wells. **(A, B)** Following 24 h incubation, apoptosis induction of cancer cells was determined by flow cytometry analysis of annexin V and PI staining. The levels of apoptosis are plotted and expressed as a mean fraction of annexin V^+^ cells ± SD of the results obtained in three independent experiments, respectively. **(C, D)** Following 24 h incubation, IFN-γ levels in the medium harvested from cultures of PBMCs with H1299 and H1975 cells were measured by an ELISA Max Deluxe Set Human IFN-γ kit. Data are expressed as IFN-γ levels ± SD of the results obtained in three independent experiments. * Indicates *P* <0.05. Abbreviations: NSCLC: Non-Small Cell Lung Cancer; PBMC: Peripheral Blood Mononuclear Cells; DMSO: Dimethyl Sulfoxide; ELISA: Enzyme-Linked Immunosorbent Assay; IFN-γ: Interferon gamma, SD: Standard Deviation, PI: propidium iodide

**Supplementary Figure S6. Apoptosis induction by Ir-2 and/or Tr-10 in cancer and non-cancer cells co-cultured with or without activated HLA-matched PBMCs.** **(A)** HCC827, **(B)** H1703, **(C)** HaCaT, and **(D)** Beas-2B cells were seeded at a density of 2 × 10^4^ per well in 24-well plates and co-cultured with or without activated HLA-matched PBMCs and treated with Tr-10 (10 μmol/L) and/or Ir-2 (1 μmol/L). PBMCs were stimulated with an anti-CD3 (1 μg/mL) and an anti-CD28 (1 μg/mL) T Cell TransAct (T Act). DMSO (vehicle) concentration was maintained at 0.01% in all wells. Following 48 h incubation, apoptosis induction was determined by flow cytometry analysis of annexin V and PI staining. The levels of apoptosis are plotted and expressed as a mean fraction of annexin V^+^ cells ± SD of the results obtained in three independent experiments. * Indicates *P* < 0.05 for Ir-2 + Tr-10 treatment in the presence of PBMCs compared with Ir-2 + Tr-10 in the absence of PBMCs and with single treatments of Ir-2 or Tr-10 in the presence of PBMCs. Abbreviations: HLA: Human Leukocyte Antigen, NSCLC: Non-Small Cell Lung Cancer; PBMC: Peripheral Blood Mononuclear Cells; DMSO: Dimethyl Sulfoxide; SD: Standard Deviation. PI: propidium iodide. Ctrl: control

**Supplementary Figure S7. Impairment of ER in NSCLC cells in the presence of Ir-2 and/or Tr-10.** H1299 and H1975 cancer cells were seeded at a density of 2 × 10^4^ per well in 24-well plates and incubated with Tr-10 (10 μmol/L) and/or Ir-2 (1 μmol/L) for 24 h. DMSO (vehicle) concentration was maintained at 0.01% in all wells. GRP78/Bip expression levels were determined by Western blotting. Data were normalized to GAPDH protein levels and expressed as the fold change *vs.* untreated cells, set as 1. **(A, B)** Densitometric analysis of GRP78/Bip expression levels in cancer cell lines. **(C, D)** Representative results of Western blotting and densitometric analysis of GRP78/Bip expression levels are shown. The results were obtained in three independent experiments. * and *** indicate *P* <0.05 and *P* <0.001, respectively. Abbreviations: NSCLC: Non-Small Cell Lung Cancer; PBMC: Peripheral Blood Mononuclear Cells; DMSO: Dimethyl Sulfoxide; GAPDH: Glyceraldehyde-3-Phosphate Dehydrogenase; GRP78/BiP: Glucose-Regulated Protein 78 / Binding Immunoglobulin Protein; Ctrl: control; ns: not significant; SD: Standard Deviation.

**Supplementary Figure S8. Modulation of DAMP emission after Ir-2 treatment.** H1975 cells were seeded into 6-well plates at a density of 2 × 10^5^ cells per well and incubated with Ir-2 (1 μmol/L). DMSO (vehicle) concentration was maintained at 0.01% in all wells. **(A)** Following 24h incubation at 37°C in a 5% CO_2_ atmosphere, intra- and extracellular ATP levels (pg/mL) were evaluated in the culture media with an ATP assay Kit. **(B)** Following 24 h and 48 h incubation at 37°C in a 5% CO_2_ atmosphere, HMGB1 and CRT levels in culture media were analyzed by Western blotting with specific mAbs. Representative results are shown. ** *P* <0.01 and *** *P* <0.001, respectively. Abbreviations: DAMPs: Damage-Associated Molecular Patterns; DMSO: Dimethyl Sulfoxide; ATP: Adenosine Triphosphate; HMGB1: High Mobility Group Box 1; CRT: Calreticulin

**Supplementary Figure S9. Impairment of ER in NSCLC cells in the presence of Ir-2 and/or Tr-10.** H1299 and H1975 cells were seeded at a density of 2 × 10^4^ per well in 24-well plates and incubated with Tr-10 (10 μmol/L) and/or Ir-2 (1 μmol/L). DMSO (vehicle of Ir-2 and Tr-10) concentration was maintained at 0.01% in all wells. Following a 24 h incubation at 37°C in a 5% CO_2_ atmosphere, cells were harvested and cell surface stained with CRT- and CNX-specific mAbs. Mouse IgG was used as a specificity control of mouse antibodies. Cell staining was detected by R- PE-conjugated F(ab’)2 fragment goat anti-mouse IgG. Data are expressed as MFI ± SD of the results obtained in three independent experiments. * *P* <0.05 and *** *P* <0.001, respectively. Abbreviations: ER: Endoplasmic Reticulum; NSCLCs: Non-Small Cell Lung Cancers; DMSO: Dimethyl Sulfoxide; CRT: Calreticulin; CNX: Calnexin; PE: Phycoerythrin; MFI: Mean Fluorescence Intensity; SD: Standard Deviation. Ctrl: control.

**Supplementary Figure S10. Detection of HLA class I, II and β2 microglobulin in cancer cells in the presence of Ir-2 and/or Tr-10. (A, B)** H1299 and **(C, D)** H1975 cancer cells were seeded at a density of 2 × 10^4^ per well in 24-well plates and incubated with Tr-10 (10 μmol/L) and/or Ir-2 (1 μmol/L). DMSO (vehicle) concentration was maintained at 0.01% in all wells. Following 24 h and 48 h incubation, cells were harvested and cell surface stained with mAbs against HLA-A, HLA-AB, HLA-ABC, β2-microglobulin light chain, HLA-DR-DQ-DP antigen-specific. Mouse IgG was used as a specificity control of mouse antibodies. Cell staining was detected by R- PE-conjugated F(ab’)2 fragment goat anti-mouse IgG. Data are expressed as MFI ± SD of the results obtained in three independent experiments and expressed as the fold change *vs.* untreated cells, set as 1. No statistically significant differences were observed in the expression of HLA-A, HLA-AB, HLA-ABC, β2-microglobulin light chain, HLA-DR-DQ-DP between treated and untreated Ctrl. Abbreviations: HLA-A: Human Leukocyte Antigen A; HLA-AB: Human Leukocyte Antigen A and B; HLA-ABC: Human Leukocyte Antigen A, B, and C; HLA-DR-DQ-DP: Human Leukocyte Antigen DR, DQ, and DP; DMSO: Dimethyl Sulfoxide; PE: Phycoerythrin; MFI: mAbs: monoclonal antibodies; Mean Fluorescence Intensity; SD: Standard Deviation; IgG: Immunoglobulin G; Ctrl: control.

**Supplementary Figure S11. Detection of PD-L1 in cancer cells in the presence of Ir-2 and/or Tr-10.** H1299 and H1975 cancer cells were seeded at a density of 2 × 10^4^ per well in 24-well plates and incubated with Tr-10 (10 μmol/L) and/or Ir-2 (1 μmol/L). DMSO (vehicle) concentration was maintained at 0.01% in all wells. Following 48 h incubation, cells were harvested and their surface stained with the indicated PD-L1 antigen-specific mAbs. Cell surface was stained with an APC anti-PD-L1 IgG2b mouse mAb. Mouse APC-conjugated IgG2b was used as a specificity control. Data are expressed as PD-L1 levels ± SD of the results obtained in three independent experiments and expressed as the fold change *vs.* Ctrl. No statistically significant differences were observed in the expression of PD-L1 between treated and untreated Ctrl. Abbreviations: PD-L1: Programmed Death-Ligand 1, DMSO: Dimethyl Sulfoxide, APC: Allophycocyanin; IgG2b: Immunoglobulin G subclass 2b; mAb: monoclonal antibody; Ctrl: control.

**Supplementary Figure S12. Impairment of mitochondrial network and increase of oxidative stress in NSCLC cells in the presence of Ir-2 and/or Tr-10. (A)** H1299 and **(B)** H1975 cells were seeded at a density of 2 × 10^5^ per well in 6-well plates on glass coverslips. Following 24 h incubation with Tr-10 (10 μmol/L) and/or Ir-2 (1 μmol/L), cells were stained with the mitoTracker red CMXRos probe for 20 min, at 37°C, prior to being fixed and processed for immunofluorescence. DMSO (vehicle) concentration was maintained at 0.01% in all wells. Representative images of single confocal sections are shown. A 4× cropped region is presented in the lower panels to highlight the mitochondrial network fragmentation. **(C)** H1299 and H1975 cells were seeded at a density of 2 × 10^4^ per well in 24-well plates and incubated for 24 h with Tr-10 (10 μmol/L) and/or Ir-2 (1 μmol/L) in the presence or absence of stimulated HLA-matched PBMCs. DMSO (vehicle) concentration was maintained at 0.01% in all wells. ROS quantification was carried out using MitoSOX fluorescence by flow cytometry. Data show mean values ± SD of ROS levels calculated on three independent experiments and expressed as fold change *vs*. untreated cells, set as 1. *** *P* <0.001. Abbreviations: NSCLCs: Non-Small Cell Lung Cancers; DMSO: Dimethyl Sulfoxide; HLA: Human Leukocyte Antigen; PBMCs: Peripheral Blood Mononuclear Cells; ROS: Reactive oxygen species; Ctrl: control.

**Supplementary Figure S13. Effects of exposure to Ir-2 and Co-3 to Co-6 on cell viability of NSCLC and non-cancer cells.** **(A)** H1975, **(B)** H1299, **(C)** HaCaT and **(D)** PBMCs were seeded at a density of 1 × 10^4^ per well in 96-well plates and exposed to increasing concentrations of Ir-2 and Co-3 to Co-6 (1-100 μmol/L). DMSO (vehicle) concentration was maintained at 0.01% in all wells. Following 48 h incubation, cell viability was determined by CCK-8 assay. Data are expressed as percentages of cell viability ± SD, of treated cells as compared with untreated cells, set as 100%, obtained in three independent experiments. Abbreviations: NSCLC: Non-Small Cell Lung Cancer; PBMC: Peripheral Blood Mononuclear Cells; DMSO: Dimethyl Sulfoxide; CCK-8: Cell Counting Kit-8; SD: Standard Deviation.

**Supplementary Figure S14. Effects of Co-3 to Co-6 on cell uptake in NSCLC cells. (A)** H1975 and **(B)** H1299 cells were seeded at a density of 2 × 10^5^ per well in 6-well plates on glass coverslips and incubated with the IC_50_ of conjugates Co-3 to Co-6. DMSO (vehicle) concentration was maintained at 0.01% in all wells. Following 3 h incubation, at 37 °C in a 5% CO_2_ atmosphere, cellular uptake was determined by confocal microscope. Cells were stained with PD-L1-specific mAbs. Cy5-linked secondary antibody (red) and DAPI (blue) were used. The wavelength emission of Ir(III) complex conjugates Co-3 to Co-6 was detected using the FITC channel (green). Representative images from a confocal z-stack with orthogonal side-views of **(A)** H1975 and **(B)** H1299 cells are shown. Abbreviations: NSCLC: Non-Small Cell Lung Cancer; DMSO: Dimethyl Sulfoxide; DAPI: 4′,6-diamidino-2-phenylindole; FITC: Fluorescein Isothiocyanate; Ctrl: control.

**Supplementary Figure S15. Uptake of Co-3-to Co-6 determined by flow cytometry. (A)** H1299 and **(B)** H1975 cells were seeded at a density of 2 × 10^4^ per well and incubated with IC_50_ doses of Co-3-6 for 2 h. DMSO (vehicle) concentration was maintained at 0.01% in all wells. Their wavelength emission was read at 516 nm, using a flow cytometer. **(C)** Data were expressed as MFI fold change compared to untreated cells. Abbreviations: DMSO: Dimethyl Sulfoxide; MFI, mean fluorescence intensity.

**Supplementary Figure S16. Effects of Co-3 to Co-6 on IFN-γ release in NSCLC cells co-cultured with activated PBMCs.** Cells were seeded at a density of 2 × 10^4^ per well in 24-well plates and co-cultured with activated PBMCs and treated with Tr-10 (10 μmol/L) and/or Ir-2 or conjugates Co-3 to Co-6 (1 μmol/L). PBMCs were stimulated with an anti-CD3 (1 μg/mL) and an anti-CD28 (1 μg/mL) T Cell TransAct (T Act). DMSO (vehicle) concentration was maintained at 0.01% in all wells. Following 48 h incubation, IFN-γ levels in the medium harvested from cultures of PBMCs with cancer cells were measured by an ELISA Max Deluxe Set Human IFN-γ kit. Data are expressed as IFN-γ levels ± SD of the results obtained in three independent experiments. ** *P* <0.01 and *** *P* <0.001 of Co-3 treatment *vs.* Untreated cells or other treatments. Abbreviations: NSCLC: Non-Small Cell Lung Cancer; PBMCs: Peripheral Blood Mononuclear Cells; DMSO: Dimethyl Sulfoxide; FN-γ: Interferon gamma; ELISA: Enzyme-Linked Immunosorbent Assay; SD: Standard Deviation; Ctrl: control.

**Supplementary Figure S17. CETSA-based determination of binding between PD-L1 and conjugate Co-3 or Tr-10 in NSCLC cells.** H1975 cells were seeded at a density of 2 × 10^6^ per plate and incubated with Co-3 or Tr-10 at the IC_50_ value (32.4 μmol/L and 50 μmol/L, respectively) for 2 h, subjected to 5 min incubation at the indicated temperatures, and then harvested and lysed. DMSO (vehicle) concentration was maintained at 0.01% in all plates. **(A)** PD-L1 expression levels were determined by Western blotting and normalized to GAPDH levels. PD-L1 level at 45°C was set at 100%. **(B)** Data are expressed as PD-L1 levels ± SD of the results obtained in three independent experiments. Ctrl consisted of untreated cells subjected to the same temperature gradient, serving as a baseline for protein thermal stability. Abbreviations: CETSA: Cellular Thermal Shift Assay; DMSO: Dimethyl Sulfoxide; PD-L1: Programmed Death-Ligand 1; GAPDH: Glyceraldehyde-3-Phosphate Dehydrogenase; Ctrl: control.


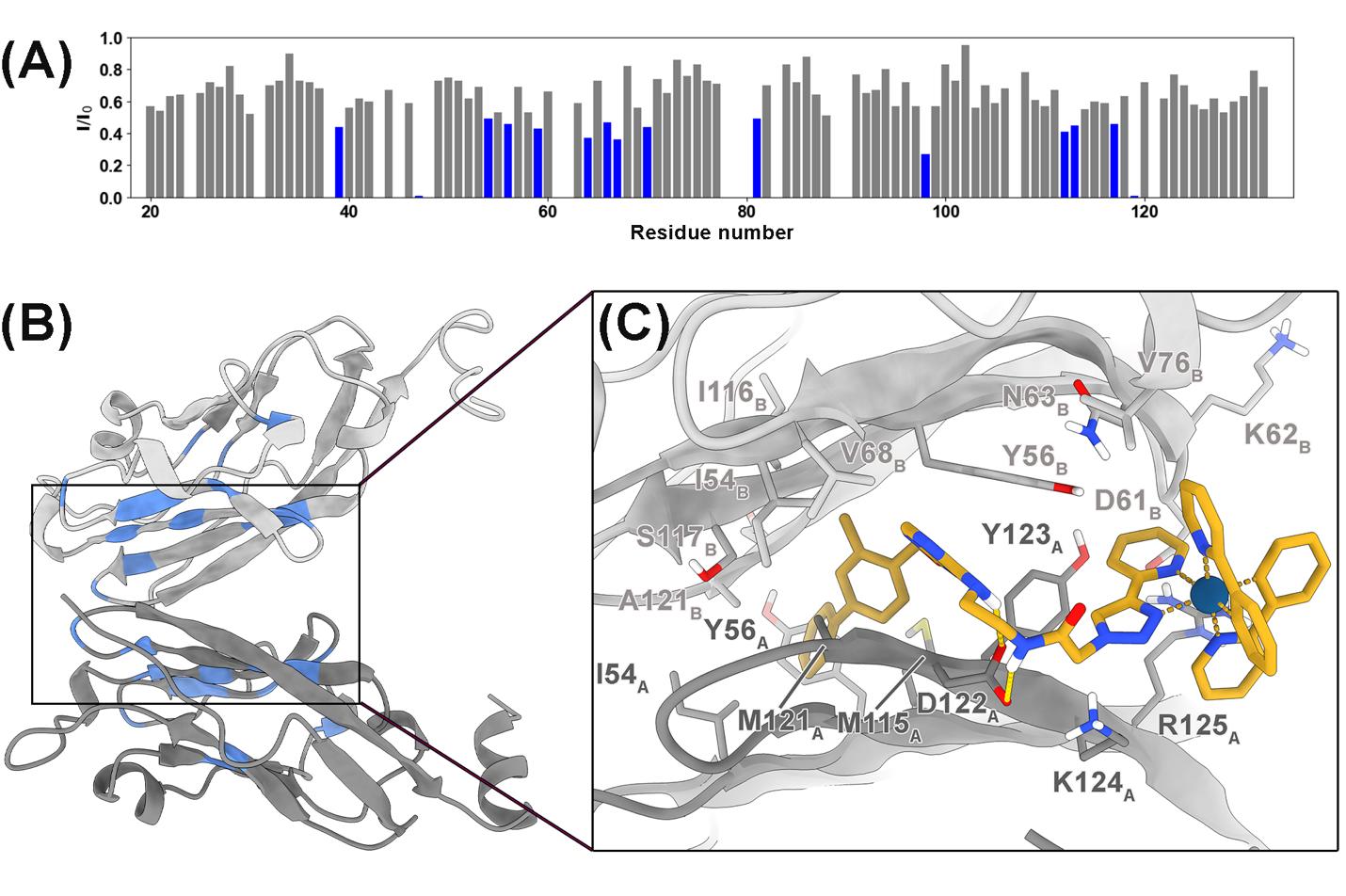


**Supplementary Figure S18. Interaction model of Co-3 with PD-L1. (A)** Per-residue intensity changes in hPD-L1 (50 μmol/L) in the presence of 50 μmol/L of Co-3, mapped onto the superimposed 3D structures of PD-L1 used in docking calculations. Residues showing the largest changes are highlighted in blue. **(B)** Mapping of residues with the highest intensity changes (highlighted in blue in (A)), as observed in NMR experiments, onto the 3D structure of the PD-L1 dimer (PDB ID: 5J89). **(C)** Lowest energy docking pose of Co-3 at the homodimeric PD-L1 binding site. Protein monomers A and B are depicted as dark and light gray cartoons, respectively, while residues important for ligand binding are shown as sticks. Co-3 is shown as yellow sticks; the iridium atom is depicted as a dark blue sphere. Hydrogen bonds are represented as yellow dashed lines, and non-polar hydrogens are omitted for clarity. Abbreviations: I/I₀, ratio of NMR signal intensity in the presence (I) and absence (I₀) of ligand hPD-L1: human Programmed Death-Ligand 1; NMR: Nuclear Magnetic Resonance; PDB ID: Protein Data Bank Identifier.


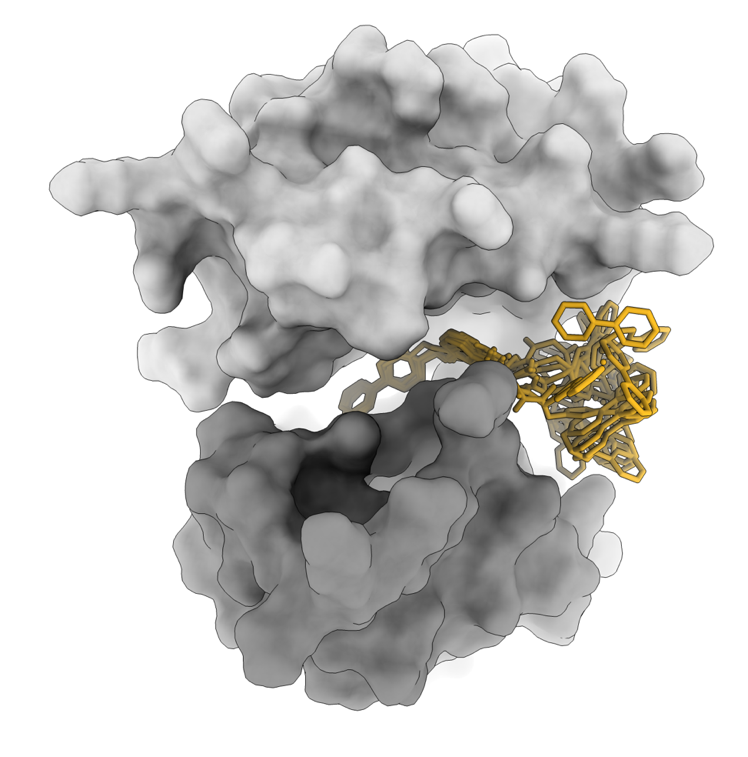


**Supplementary Figure S19. Superposition of all the docking poses of Co-3 at the homodimeric PD-L1 dimer binding site** (PDB ID: 5J89 [[10.18632/oncotarget.8730](https://doi.org/10.18632/oncotarget.8730)]. The ligand is shown as yellow sticks, while the two protein monomers are depicted in light and dark gray surfaces. Abbreviations: PD-L1: Programmed Death-Ligand 1; PDB ID: Protein Data Bank Identifier.

**Supplementary Figure S20. Transfection and detection of PD-L1 expression levels in generated H1975 PD-L1 KO clones.** H1975 cells were seeded at a density of 2 × 10^4^ per well in 24-well plates. PD-L1 knock out was performed using short guide RNAs (sgRNAs) against the PD-L1 gene; sgCD274 #1 (5’- TGGCTGCACTAATTGTCTAT -3’) and sgCD274 #2 (5’- ACCCCAAGGCCGAAGTCATC-3’). As a control, an sgRNA with no homology to the human genome (non-target control, Ctrl) was used, and ribonucleoprotein (RNP) complexes composed of 12 nmol/L of two different sgRNAs and 6 nmol/L Cas9 Nuclease V3 enzyme were delivered into H1975 cells using TransIT-X2^®^ Dynamic Delivery System for 72 h. The transfection efficiency was assessed by measuring TEX 615 Transfection Control siRNA expression using a confocal microscope. **(A)** Representative images from a confocal microscope of transfection efficiency in H1975 cells are shown. **(B)** Stable clones were generated by collecting transfected H1975 cells and isolating them by limiting dilution. Generated single clones were analyzed by Western blotting to verify gene inactivation. Representative results are shown. Clones 14 and 22, named H1975 *^PD-L1 KO-1^* and H1975 *^PD-L1 KO-2,^* were chosen for further characterization. **(C)** H1975 cells, transfected with sgRNA with no homologies to the human genome (non-target control), and H1975 PD-L1 KO cells (H1975 *^PD-L1 KO-1^* and H1975 *^PD-L1 KO-2^*), transfected with PD-L1-sgRNAs, were seeded at a density of 2 × 10^5^ per 6-well plate. PD-L1 expression was evaluated by flow cytometry. Cells were stained with Allophycocyanin (APC)-conjugated PD-L1 IgG2b mAb. APC-conjugated IgG2b isotype was used as a control for PD-L1-specific mAb. Data are plotted as MFI of PD-L1 expression and side scatter (SSC-A) of cancer cells. Percentages of PD-L1-positive cells are indicated. Representative results from three independent experiments are shown. Abbreviations: sgRNA: single-guide RNA; CD274: Cluster of Differentiation 274; MFI: Mean Fluorescence Intensity; PD-L1: Programmed Death-Ligand 1; SSC-A: Side Scatter Area; KO: Knockout; IgG2b: Immunoglobulin G subclass 2b; mAb: monoclonal antibody; RNP: ribonucleoprotein; APC: Allophycocyanin; Ctrl: control.

**Supplementary Figure S21. Effects of Co-3 on CRT exposure in H1975 and generated H1975 PD-L1 KO clones.** H1975 *^Ctrl^*, H1975 *^PD-L1 KO-1^* and H1975 *^PD-L1 KO- 2^* cells were seeded at a density of 2 × 10^4^ per well in 24-well plates. Following 48 h incubation at 37°C in a 5% CO_2_ atmosphere, cells were harvested and cell surface stained with CRT-specific mAbs. Mouse IgG (MK2-23) was used as a specificity control of the mouse antibody. Cell staining was detected by R- PE-conjugated F(ab’)2 fragment goat anti-mouse IgG. PCTX (50 nmol/L) was used as a positive control. **(A)** Data are plotted as MFI of CRT expression and side scatter (SSC-A) of cancer cells. Percentages of CRT-positive cells are indicated. Representative results from three independent experiments are shown. **(B)** The result of the densitometric analysis of HMGB1 release levels is shown. The results were obtained in three independent experiments. * Indicates *P* <0.05. Abbreviations: CRT: Calreticulin; PD-L1: Programmed Death-Ligand 1; SSC-A: Side Scatter Area; IgG MK2-23: Immunoglobulin G isotype control (clone MK2-23); PCTX: Paclitaxel; MFI: Mean Fluorescence Intensity; HMGB1: High Mobility Group Box 1; KO: Knockout; Ctrl: control

**Supplementary Figure S22. Effects of Co-3-6 on apoptosis induction in HaCaT cells co-cultured with activated PBMCs.** HaCaT were seeded at a density of 2 × 10^4^ per well in 24-well plates and co-cultured with activated PBMCs and treated with Tr-10 (10 μmol/L) or Ir-2 or conjugates Co-3 to Co-6 (1 μmol/L). DMSO concentration was maintained at 0.01% in all wells. Following 48 h incubation, apoptosis induction was determined by flow cytometry analysis of annexin V and PI staining. The levels of apoptosis are plotted and expressed as a mean fraction of annexin V^+^ cells ± SD of the results obtained in three independent experiments. Abbreviations: PBMCs: Peripheral Blood Mononuclear Cells; DMSO: Dimethyl Sulfoxide; PI: Propidium Iodide; SD: Standard Deviation; Ctrl: control.

**Supplementary Figure S23. Detection of PD-L1 in myeloid cells from a healthy donor and an NSCLC patient.** PBMCs from a healthy donor and an NSCLC patient were used to isolate monocytes by magnetic separation. The immature dendritic cells were then obtained by culturing monocytes CD14^+^ in RPMI supplemented with 50 ng/mL granulocyte macrophage colony-stimulating factor (GM-CSF), and 1,000 U/mL IL-4. The macrophages were obtained by culturing monocytes CD14^+^ in ImmunoCult-SF Macrophage Differentiation Medium. After differentiation, monocytes, dendritic cells and macrophages were harvested and stained with APC-Cy7-conjugated anti-CD14, APC-Cy7-conjugated anti-CD1a or APC-Cy7-conjugated anti-CD68 monoclonal antibodies, respectively. Representative flow cytometry plots for the **(A)** healthy donor and the **(B)** NSCLC patient are shown. Cell surface was stained with an APC anti-PD-L1 IgG2b mAb. APC-conjugated IgG2b was used as a specificity control. Data are expressed as MFI ± SD of the results obtained in three independent experiments. Abbreviations: NSCLC: Non-Small Cell Lung Cancer; PBMCs: Peripheral Blood Mononuclear Cells; GM-CSF: Granulocyte-Macrophage Colony-Stimulating Factor; APC: Allophycocyanin; PD-L1: Programmed Death-Ligand 1; IgG2b: Immunoglobulin G subclass 2b; CD1a: Cluster of differentiation 1a; CD14: Cluster of differentiation 14; CD68: Cluster of differentiation 68; IL-4: Interleukin 4; mAb: monoclonal antibody; APC-Cy7: Allophycocyanin–Cyanine 7; MFI: Mean Fluorescence Intensity; SD: Standard Deviation.

**Supplementary Figure S24. Effects of exposure to Co-3 on PD-L1⁺CD14⁺ monocytes from a healthy donor and an NSCLC patient.** PBMCs from a healthy donor and an NSCLC patient were used to isolate monocytes by magnetic separation. Purified monocytes were treated with Co-3 (1 μmol/L) for 24 h. After incubation, cells were harvested and stained with APC-conjugated anti-PD-L1 and APC-Cy7-conjugated anti-CD14 monoclonal antibodies. PI was added immediately before acquisition to assess the cell viability. Representative flow cytometry plots for the **(A)** healthy donor and the **(B)** NSCLC patient are shown. Monocytes were first identified on SSC-A versus FSC-A plots, and PD-L1⁺CD14⁺ monocytes were subsequently gated. PI uptake was then evaluated in PD-L1⁺ and CD14⁺ monocyte subsets. Numbers in red boxes indicate the percentage of PI⁺ cells within the indicated gates. Abbreviations: NSCLC: Non-Small Cell Lung Cancer; PBMCs: Peripheral Blood Mononuclear Cells; APC: Allophycocyanin; CD14 Cluster of differentiation 14; APC-Cy7: Allophycocyanin–Cyanine 7; PI: Propidium Iodide; SSC-A: Side Scatter Area; FSC-A: Forward Scatter Area; Ctrl: control.

**Supplementary Figure S25. Effects of exposure to Co-3 on PD-L1⁺CD1a⁺ monocyte-derived dendritic cells from a healthy donor and an NSCLC patient.** Peripheral blood mononuclear cells (PBMCs) from a healthy donor and an NSCLC patient were used to isolate monocytes by magnetic separation. The immature dendritic cells were then obtained by culturing monocytes CD14^+^ in RPMI supplemented with 50 ng/mL granulocyte macrophage colony-stimulating factor (GM-CSF), and 1,000 U/mL IL-4. Monocyte-derived dendritic cells were treated with Co-3 (1 μmol/L) for 24 h. After incubation, cells were harvested and stained with APC-conjugated anti-PD-L1 and APC-Cy7-conjugated anti-CD1a monoclonal antibodies. Propidium iodide (PI) was added immediately before acquisition to assess the cell viability. Representative flow cytometry plots for the **(A)** healthy donor and the **(B)** NSCLC patient are shown. Monocyte-derived dendritic cells were first identified on SSC-A versus FSC-A plots, and PD-L1⁺CD1a⁺ dendritic cells were subsequently gated. PI uptake was then evaluated in PD-L1⁺ and CD1a⁺ monocyte subsets. Numbers in red boxes indicate the percentage of PI⁺ cells within the indicated gates. Abbreviations: NSCLC: Non-Small Cell Lung Cancer; PBMCs: Peripheral Blood Mononuclear Cells; APC: Allophycocyanin; APC-Cy7: Allophycocyanin–Cyanine 7; PI: Propidium Iodide; PD-L1: Programmed death-ligand 1; CD1a: Cluster of differentiation 1a; CD14: Cluster of differentiation 14; IL-4: Interleukin 4; SSC-A: Side Scatter Area; FSC-A: Forward Scatter Area; Ctrl: control.

**Supplementary Figure S26. Evaluation of IFN-γ release by activated PBMCs and/or PBLs CD14^–^ and CD11b ^–^ co-cultured with NSCLC cells in the presence of Co-3.** Peripheral blood lymphocytes (PBLs) were generated by depleting CD14⁺ or CD11b⁺ cells from peripheral blood mononuclear cells (PBMCs) from a healthy donor, removing the major myeloid populations, including monocytes, macrophages, dendritic cells, and granulocytic cells. H1975 cells were seeded at a density of 2 × 10^4^ per well in 24-well plates, co-cultured with activated PBMCs or PBLs and treated with Co-3 (1 μmol/L). PBMCs or PBLs were stimulated with an anti-CD3 (1 μg/mL) and an anti-CD28 (1 μg/mL) T Cell TransAct (T Act). DMSO (vehicle) concentration was maintained at 0.01% in all wells. Following 48h incubation, IFN-γ levels in the medium harvested from cultures of PBMCs or PBLs with cancer cells were measured by an ELISA Max Deluxe Set Human IFN-γ kit. Data are expressed as IFN-γ levels ± SD of the results obtained in three independent experiments. ** *P* < 0.01 *and* *** *P* < 0.001, respectively. Abbreviations: PBLs: Peripheral Blood Lymphocytes, PBMCs: Peripheral Blood Mononuclear Cells; DMSO: Dimethyl Sulfoxide, CD11b: Cluster of differentiation 11b; CD14: Cluster of differentiation 14; FN-γ: Interferon gamma; ELISA: Enzyme-Linked Immunosorbent Assay; SD: Standard Deviation; Ctrl: control.

**Synthetic procedure for PD-L1 ligand-Ir(III) complex conjugates Co-3 to Co-6.**

The synthesis of disubstituted PD-L1 ligand-Ir(III) conjugates Co-3 and Co-4 started with a nucleophilic substitution of chloro-biphenyl ether 7 with azido-amines 8 and 9 (***step c***, Scheme S21), previously obtained in good yields by coupling commercial mono-Boc-ethylendiamine with either 3-azidopropanoic acid or 4-azidobutanoic acid, followed by Boc deprotection under standard conditions (***steps a,b***). Intermediate azides 10 and 11 were obtained in good yields and submitted to a copper-catalyzed Huisgen 1,3-dipolar cycloaddition with commercial 2-ethynylpyridine, yielding advanced precursors 12 and 13 in good yields (***step d***). Finally, targeted Ir(III) conjugates Co-3 and Co-4 were synthesized in good yields from commercial dimeric [Ir(ppy)_2_Cl]_2_ through an optimized, precedented procedure (***step e***, **Supplementary Figure S27**) involving a ligand metathesis reaction.

**Supplementary Figure S27. Synthetic procedure for PD-L1 ligand-Ir(III) conjugates Co-3 and Co-4.** a) EDC·HCl, HOBt, DIPEA, dry DCM, N_2_, r.t., 24 h; b) 1:1 TFA/DCM, r.t., 1h, 71% over two steps (8), 67% over two steps (9); c) DIPEA, dry CH_3_CN, N_2_, 70°C, 6 h, 74% (10), 84% (11); d) 2-ethynylpyridine, sodium ascorbate, CuSO_4_∙5H_2_O, 1:1 THF/H_2_O, r.t., 6 h, 73% (12), 61% (13); e) [Ir(ppy)_2_Cl]_2_, dry and degassed 3:2 DCM/MeOH, Ar, r.t., 24 h, 81% (Co-3), 77% (Co-4). Abbreviations: Boc: tert-butyloxycarbonyl; DCM: dichloromethane; DIPEA: N,N-diisopropylethylamine; EDC∙HCl: (3-Dimethylamino-propyl)-ethyl-carbodiimide hydrochloride; HOBt: hydroxybenzotriazole; THF: tetrahydrofuran; TFA: trifluoroacetic acid; Ppy: phenylpyridine[

The synthesis of trisubstituted PD-L1 ligand-Ir(III) conjugates Co-5 and Co-6 started from dichloro biphenyl ether triazine 14, reacted in mild nucleophilic conditions (-20 °C to r.t.), with either commercial 2-azidoethan-1-amine or 6-azidohexan-1-amine (step a, S**upplementary Figure S28**), providing intermediate azide chlorotriazines 15 and 16 in good yields. Intermediates 15 and 16 were then reacted with N-(2-aminoethyl)-acetamide (step b) at higher temperature (70°C), affording acetamido azides 17 and 18 in good yields. A copper-catalyzed Huisgen 1,3-dipolar cycloaddition between intermediates 17 and 18 and 2-ethynylpyridine in standard conditions (step c) yielded advanced precursors 19 and 20 in excellent yields. Finally, target Ir(III) complexes Co-5 and Co-6 were obtained as previously described (step d, Scheme S21) in good yields.

**Supplementary Figure S28. Synthetic procedure for Ir(III) conjugates Co-5 and Co-6**. a) DIPEA, dry DCM, N_2_, -20 °C to r.t., 6 h, 91% (15), 86% (16); b) N-(2-aminoethyl)-acetamide, DIPEA, dry CH_3_CN, N_2_, 70°C, 7 h, 58% (17), 88% (18); c) 2-ethynylpyridine, Na ascorbate, CuSO_4_∙5H_2_O, 1:1 THF/H_2_O, r.t., 6 h, 85% (19), 95% (20); d) [Ir(ppy)_2_Cl]_2_, dry and degassed 3:2 DCM/MeOH, Ar, r.t., 24 h, 68% (Co-5), 72% (Co-6). Abbreviations: Boc: tert-butyloxycarbonyl; DCM: dichloromethane; DIPEA: N,N-diisopropylethylamine; EDC∙HCl: (3-Dimethylamino-propyl)-ethyl-carbodiimide hydrochloride; HOBt: hydroxybenzotriazole; THF: tetrahydrofuran; TFA: trifluoroacetic acid; Ppy: phenylpyridine[

*Chemistry*

Reagent-grade chemicals and solvents were purchased from Sigma-Aldrich or FluoroChem, and were used without any further purification. Dry solvents were purchased from Sigma-Aldrich, and reactions in anhydrous conditions were performed under a nitrogen atmosphere, using a dry nitrogen flux (passage through Drierite (Ca_2_SO_4_) traps as a drying agent) or, in alternative, under argon. Reactions were monitored by analytical thin-layer chromatography (TLC), using silica gel 60 F254 pre-coated glass plates (0.25 mm thickness). Visualization was accomplished by irradiation with a UV lamp and/or staining with alcoholic ninhydrin solution or cerium/molybdate reagent. Purifications were carried out either by direct phase flash chromatography on Macherey-Nagel silica gel (particle size 60 µm, 230–400 mesh), or through an automated Biotage^TM^ chromatography system. NMR spectra were recorded on a Bruker Advance 400 instrument in deuterated solvents either at 400 MHz (^1^H-NMR) or at 101 MHz (^13^C NMR). Chemical shifts are expressed in δ (ppm) with tetramethylsilane (TMS) employed as an internal standard. Coupling constants are given in Hertz and rounded to the nearest 0.1 Hz. The following abbreviations are used to describe spin multiplicity: s = singlet, d = doublet, t = triplet, q = quartet, m = multiplet, bs = broad signal, dd = doublet of doublets. LC–MS data were collected with a Waters Acquity Ultra performance LC (UPLC)-MS System equipped with a TUV detector, a single quadrupole SQD mass spectrometer and ACQUITY UPLC BEH SHIELD RP_18_ columns (2.1x100mm, id=1.7 µm). HPLC compounds’ purity was determined by monitoring at 220 nm using H_2_O (HiPerSolv Chromanorm Water VWR for HPLC-MS) + 0.05% trifluoroacetic acid (spectroscopic grade) (phase A) and acetonitrile (HiPerSolv Chromanorm Acetonitrile SuperGradient VWR) + 0.05% trifluoroacetic acid (spectroscopic grade) (phase B), and a gradient from 5% B to 100% B in 5 min, followed by 100% B for 1 min; a 0.5 mL/min flow was used, with 2 min equilibration time. UV/V is absorption spectra were obtained on a Shimadzu UV-Vis-NIR 3600 spectrophotometer in 1 cm path length quartz cell at a concentration of ca. 1-2 x 10^-5^ mol/L. Photoluminescence quantum yields were measured with a C11347 Quantaurus – QY Absolute Photoluminescence Quantum Yield Spectrometer (Hamamatsu Photonics U.K), equipped with a 150 W Xenon lamp, an integrating sphere and a multi-channel detector. Steady state emission and excitation spectra and photoluminescence lifetimes were obtained with a FLS 980 spectrofluorimeter (Edinburgh Instrument Ltd.). Continuous excitation for the steady state measurements was provided by a 450 W Xenon arc lamp. Photoluminescence lifetime measurements were determined by TCSPC (time-correlated single-photon counting) method, using an Edinburgh Pulsed Diode PLED-405 (Edinburgh Instrument Ltd.), with a central wavelength of 405 nm, output power 0.09-0.11 mW and pulse rates of 90-60ps or greater. Photoluminescence experiments at room temperature were carried out in nitrogen-degassed dichloromethane solution at 1-2x10^-5^ mol/L. Measurements at 77 K were performed in anhydrous 2-methyl-tetrahydrofuran frozen matrix.

*Synthesis of tert-butyl (2-(3-azidopropanamido)ethyl) carbamate* 8. (3-Dimethylamino-propyl)-ethyl-carbodiimide hydrochloride (EDC∙HCl) (248.6 mg, 1.30 mmol, 1.5 eq) and hydroxybenzotriazole (HOBt) (176.1 mg, 1.30 mmol, 1.5 eq) were sequentially added under nitrogen atmosphere to a solution of 3-azidopropanoic acid (100.0 mg, 0.869 mmol, 1 eq) in dry dichloromethane (DCM) (2.3 mL). The mixture was vigorously stirred at room temperature for 30 minutes until the solution became clear, then tert-butyl(2-aminoethyl)carbamate (153.1 mg, 0.956 mmol, 1.1 eq) in dry DCM (2.0 mL) and N,N-diisopropylethylamine (DIPEA) (0.453 mL, 2.61 mmol, 3 eq) were sequentially added. The mixture was stirred under a nitrogen atmosphere, monitored by TLC (eluent mixture: 95:5 DCM/MeOH, developed in ninhydrin). After 24hours, the mixture was washed with 1M HCl (5 mL), a saturated solution of NaHCO_3_ (5 mL) and brine (5 mL). The organic phase was dried over sodium sulfate and filtered. The solvent was removed under reduced pressure, and the crude was purified by flash chromatography (eluent mixture: 95:5 DCM/MeOH), to afford 158.1 mg (0.614 mmol) of tert-butyloxycarbonyl (Boc)-protected linker. The latter was dissolved in dry DCM (0.80 mL), and trifluoroacetic acid (TFA) (0.424 mL, 5.526 mmol, 9 eq) was added dropwise. The reaction mixture was stirred at room temperature for 1 hour, monitored by TLC (eluent mixture: 95:5 DCM/MeOH, developed in molybdic reagent or ninhydrin). After reaction completion, the solvent was removed under reduced pressure, the residue was dissolved in toluene (1 mL), and the solvent was evaporated under reduced pressure to remove any trace of TFA; toluene addition and stripping were repeated three times. As a result, 166.5 mg of pure target azide 8 were obtained as a TFA salt (0.614 mmol, 71% yield over two steps) and used in the following reaction step without further purification. MS (ESI^+^): *m/z* 158.16 [M+H]^+^, calculated for C_5_H_11_N_5_O 157.10. ^1^H NMR (400 MHz, CD_3_OD) δ: 3.58 (t, *J* = 6.4 Hz, 2H, COCH_2_), 3.47 (t, *J* = 6.0 Hz, 2H, NHCH_2_), 3.06 (t, *J* = 6.0 Hz, 2H, CH_2_NH_2_), 2.50 (t, *J* = 6.4 Hz, 2H, N_3_CH_2_). ^13^C NMR (101 MHz, CD_3_OD) δ: 174.3, 48.3, 40.8, 38.3, 36.1 (**Supplementary Figure S29**).

*Synthesis of tert-butyl (2-(5-azidopentanamido)ethyl) carbamate* *9*. EDC∙HCl (200.6 mg, 1.049 mmol, 1.5 eq) and HOBt (141.7 mg, 1.049 mmol, 1.5 eq) were sequentially added under a nitrogen atmosphere to a solution of 5-azidopentynoic acid (100.0 mg, 0.699 mmol, 1 eq) in dry DCM (2 mL). The mixture was vigorously stirred at room temperature for 30 minutes, until the solution became clear. Then, tert-butyl(2-aminoethyl) carbamate (123.1 mg, 0.728 mmol, 1.1 eq) in dry DCM (1.5 mL) and DIPEA (0.365 mL, 2.097 mmol, 3 eq) were sequentially added. The mixture was stirred under a nitrogen atmosphere, monitored by TLC (eluent mixture: 95:5 DCM/MeOH, developed in ninhydrin). After 24hours and reaction completion, the mixture was washed with 1M HCl (5 mL), a saturated solution of NaHCO_3_ (5 mL) and brine (5 mL). The organic phase was dried over sodium sulfate and filtered. The solvent was removed under reduced pressure, and the crude was purified by flash chromatography (eluent mixture: 95:5 DCM/MeOH), to afford 133.0 mg (0.466 mmol) of Boc-protected linker. The latter was dissolved in dry DCM (0.60 mL), and TFA (0.321 mL, 4.194 mmol, 9 eq) was added dropwise. The reaction mixture was stirred at room temperature for 1 hour, monitored by TLC (eluent mixture: 95:5 DCM/MeOH, developed in molybdic reagent and ninhydrin). After reaction completion, the solvent was removed under reduced pressure. The residue was dissolved in toluene (1 mL), and the solvent was evaporated under reduced pressure, to remove any trace of TFA; toluene addition and stripping were repeated three times. As a result, 139.5 mg of pure target azide 9 were obtained as a TFA salt (0.466 mmol, 67% yield over two steps) and used in the following reaction step without further purification. MS (ESI^+^): *m/z* 186.14 [M+H]^+^, calculated for C_7_H_15_N_5_O 185.13. ^1^H NMR (400 MHz, CD_3_OD) δ: 3.44 (t, *J* = 6.0 Hz, 2H, NHCH_2_), 3.34-3.31 (m, 2H, COCH_2_), 3.05 (t, *J* = 6.0 Hz, 2H, CH_2_NH_2_), 2.28 (t, *J* = 7.4 Hz, 2H, N_3_CH_2_), 1.74-1.57 (m, 4H, N_3_CH_2_CH_2_, COCH_2_CH_2_). ^13^C NMR (101 MHz, CD_3_OD) δ: 170.5, 52.1, 40.9, 38.2, 36.6, 29.4, 23.8 (**Supplementary Figure S30**).

*Synthesis of 3-azido-N-(2-((4-((2-methyl-[1,1'-biphenyl]-3-yl)methoxy)-1,3,5-triazin-2-yl)amino)ethyl) propanamide 10.* A solution of azidoamine 8 (156.5 mg, 0.577 mmol, 1.2 eq) and DIPEA (0.251 mL, 1.443 mmol, 3 eq) in dry CH_3_CN (1.2 mL) was added to a solution of 2-chloro-4-((2-methyl-[1,1'-biphenyl]-3-yl)methoxy)-1,3,5-triazine 7 (150.0 mg, 0.481 mmol, 1 eq) in dry CH_3_CN (1.2 mL) under nitrogen atmosphere. The reaction mixture was heated at 70°C and stirred for 8 hours, monitored by TLC (eluent mixture: 95:5 DCM/MeOH, developed in molybdic reagent). After reaction completion, it was cooled to room temperature and the solvent was evaporated under reduced pressure. DCM (5 mL) was added to the residue, and a saturated aqueous solution of NH_4_Cl (5 mL) was slowly added until neutral pH. Then, the mixture was extracted with DCM (5 mL), and the collected organic phases were washed with brine (20 mL), dried over sodium sulfate, filtered, and evaporated under reduced pressure. The crude was purified by Biotage^TM^ direct phase chromatography (eluent mixture: DCM/MeOH, gradient from 0% to 20% of MeOH) to afford 154.4 mg of pure target triazine azide 10 as a white solid (0.357 mmol, 74% yield). MS (ESI^+^): *m/z* 433.41 [M+H]^+^, calculated for C_22_H_24_N_8_O_2_ 432.20. ^1^H NMR (400 MHz, CDCl_3_) some signals marked as * in the text are split due to the presence of two atropoisomers in an undetermined ratio. δ: 8.41*, 8.32* (2s, 1H, H triazine), 7.43-7.33 (m, 4H, H biphenyl), 7.30-7.23 (m, 4H, H biphenyl), 6.34*, 6.21*, 6.16*, 6.08*, (4m, 2H, NH), 5.49*, 5.44* (2s, 2H, OCH_2_), 3.63-3.55 (m, 4H, NHCH_2_), 3.50-3.48 (m, 2H, COCH_2_), 2.40-2.33 (m, 2H, N_3_CH_2_), 2.28*, 2.26* (2s, 3H, CH_3_). ^13^C NMR (101 MHz, CDCl_3_) some signals are split due to the presence of two atropoisomers in an undetermined ratio. δ: 170.8, 170.3, 143.2, 143.1, 141.9, 134.8, 134.4, 134.2, 130.8, 130.6, 130.5, 129.5, 128.8, 128.5, 128.3, 128.2, 127.1, 125.9, 125.7, 68.7, 68.1, 47.5, 47.4, 45.7, 41.1, 40.9, 40.2, 39.6, 36.0, 35.9, 16.5, 16.2 (**Supplementary Figure S31**).

*Synthesis of 5-azido-N-(2-((4-((2-methyl-[1,1'-biphenyl]-3-yl)methoxy)-1,3,5-triazin-2-yl)amino)ethyl) pentanamide 11*. Target compound 11 was synthesized according to the procedure reported for 10, starting from chloroether 7 (95.5 mg, 0.306 mmol, 1 eq), azidoamine 9 (110.0 mg, 0.368 mmol, 1.2 eq) and DIPEA (0.160 mL, 0.918 mmol, 3 eq) in dry CH_3_CN (1.5 mL) at 70°C for 8 hours, monitoring by TLC (eluent mixture: 95:5 DCM/MeOH, developed in molybdic reagent). The crude was purified by Biotage^TM^ direct phase chromatography (eluent mixture: DCM/MeOH, gradient from 0% to 20% of MeOH) to afford 117.7 mg of pure target triazine amide 11 as a white solid (0.256 mmol, 84% yield). MS (ESI^+^): *m/z* 461.44 [M+H]^+^, calculated for C_24_H_28_N_8_O_2_ 460.23. ^1^H NMR (400 MHz, CDCl_3_) some signals marked as * in the text are split due to the presence of two atropoisomers in an undetermined ratio. δ: 8.41*, 8.32* (2s, 1H, H triazine), 7.43-7.30 (m, 4H, H biphenyl), 7.29-7.21 (m, 4H, H biphenyl), 6.16-6.03 (m, 2H, NH), 5.48*, 5.44* (2s, 2H, OCH_2_), 3.61-3.57 (m, 2H, NHCH_2_), 3.48-3.47 (m, 2H, NHCH_2_), 3.29-3.23 (m, 2H, COCH_2_), 2.28*, 2.26* (2s, 3H, CH_3_), 2.22-2.13 (m, 2H, N_3_CH_2_), 1.70-1.53 (m, 4H, COCH_2_CH_2_CH_2_). ^13^C NMR (101 MHz, CDCl_3_) some signals are split due to the presence of two atropoisomers in an undetermined ratio. δ: 173.4, 170.4, 170.0, 167.1, 143.1, 142.0, 141.9, 136.2, 134.7, 134.4, 130.8, 130.5, 129.5, 129.4, 129.2, 128.7, 128.5, 128.3, 128.2, 127.2, 127.1, 125.9, 125.6, 68.3, 68.1, 51.2, 45.7, 41.3, 41.1, 40.0, 39.5, 36.0, 35.9, 28.4, 22.8, 16.2 (**Supplementary Figure S32**).

*Synthesis of N-(2-((4-((2-methyl-[1,1'-biphenyl]-3-yl)methoxy)-1,3,5-triazin-2-yl)amino)ethyl)-3-(4-phenyl-1H-1,2,3-triazol-1-yl) propenamide* *12*. Freshly prepared 0.15 mol/L aqueous CuSO_4_·5H_2_O (0.217 mL, 0.033 mmol, 0.1 eq) and 0.5 mol/L aqueous sodium ascorbate (0.163 mL, 0.082 mmol, 0.25 eq) solutions were added to a stirred solution of azido-intermediate 10 (141.2 mg, 0.326 mmol, 1 eq) and 2-ethynylpyridine (33.7 mg, 0.326 mmol, 1 eq) in 1:1 tetrahydrofuran (THF)/H_2_O. The resulting mixture was stirred at room temperature for 6 hours, monitored by TLC (eluent mixture: 95:5 DCM/MeOH, developed in molybdic reagent). After reaction completion the solvent was evaporated under reduced pressure and the crude was purified by Biotage^TM^ direct phase chromatography (eluent mixture: DCM/MeOH, gradient from 0% to 20% MeOH) to afford 127.4 mg of pure target pyridotriazolyl triazine 12 as a white solid (0.237 mmol, 73% yield). MS (ESI^+^): *m/z* 536.22 [M+H]^+^, calculated for C_30_H_30_N_8_O_2_ 535.24. ^1^H NMR (400 MHz, CDCl_3_) some signals marked as * in the text are split due to the presence of two atropoisomers in an undetermined ratio. δ: 8.56 (s, 1H, H pyridine), 8.35*, 8.23* (2s, 1H, H triazine), 8.17-8.09 (m, 2H, H pyridine, H triazole), 7.76-7.70 (m, 1H, H pyridine), 7.42-7.32 (m, 4H, H biphenyl), 7.28-7.21 (m, 5H, H biphenyl, H pyridine), 6.47-6.28 (3m, 2H, NH), 5.44*, 5.36* (2s, 2H, OCH_2_), 4.76-4.73 (m, 2H, CH_2_-triazole), 3.55-4.45 (m, 4H, NHCH_2_), 2.85-2.79 (m, 2H, COCH_2_), 2.26*, 2.22* (2s, 3H, CH_3_). ^13^C NMR (101 MHz, CDCl_3_) some signals are split due to the presence of two atropoisomers in an undetermined ratio. δ: 170.3, 170.2, 169.9, 167.2, 167.1, 143.0, 142.0, 134.7, 134.6, 134.5, 130.4, 129.5, 128.6, 128.5, 128.2, 127.0, 125.6, 68.1, 67.9, 46.7, 41.2, 41.0, 39.8, 39.4, 36.8, 36.7, 16.4 (**Supplementary Figure S33**).

*Synthesis of N-(2-((4-((2-methyl-[1,1'-biphenyl]-3-yl)methoxy) -1,3,5-triazin-2-yl)amino)ethyl)-5-(4-(pyridin-2-yl)-1H-1,2,3-triazol-1-yl)pentanamide 13.* Target compound 13 was synthesized according to the procedure reported for 12, starting from azido intermediate 11 (102.0 mg, 0.221 mmol, 1 eq), 2-ethynylpyridine (22.8 mg, 0.221 mmol, 1 eq), 0.15 mol/L CuSO_4_∙5H_2_O (0.147 mL) and 0.5 mol/L Na ascorbate (0.111 mL) in 1:1 THF/H_2_O (2.2 mL), monitoring by TLC (eluent mixture: 95:5 DCM/MeOH, developed in molybdic reagent). The crude was purified by Biotage^TM^ direct phase chromatography (eluent mixture: DCM/MeOH, gradient from 0% to 20% MeOH) to afford 76.1 mg of pure pyridotriazolyl triazine target 13 as a white solid (0.135 mmol, 61% yield). MS (ESI^+^): *m/z* 564.34 [M+H]^+^, calculated for C_31_H_33_N_9_O_2_ 563.28. ^1^H NMR (400 MHz, CDCl_3_) some signals marked as * in the text are split due to the presence of two atropoisomers in an undetermined ratio. δ: 8.59-8.54 (m, 1H, H pyridine), 8.40*, 8.30* (2s, 1H, H triazine), 8.17-8.14 (m, 2H, H pyridine, H triazole), 7.78-7.73 (m, 1H, H pyridine), 7.42-7.34 (m, 5H, H biphenyl), 7.28-7.21 (m, 4H, H biphenyl, H pyridine), 6.32-6.11 (m, 2H, NH), 5.47*, 5.42* (2s, 2H, OCH_2_), 4.44-4.38 (m, 2H, CH_2_- triazole), 3.60-3.57 (m, 2H, NHCH_2_), 3.48-3.47 (m, 2H, NHCH_2_), 2.27*, 2.24* (2s, 3H, CH_3_), 2.21-2.14 (m, 2H, COCH_2_), 2.01-1.93 (m, 2H, COCH_2_CH_2_), 1.67-1.64 (m, 2H, COCH_2_CH_2_CH_2_). ^13^C NMR (101 MHz, CDCl_3_) some signals are split due to the presence of two atropoisomers in an undetermined ratio. δ: 173.1, 170.4, 170.0, 168.0, 167.5, 167.3, 150.3, 149.5, 148.6, 143.1, 142.0, 136.8, 134.7, 134.6, 134.5, 130.4, 129.5, 128.7, 128.5, 128.2, 127.0, 125.6, 123.1, 122.3, 120.4, 68.2, 67.9, 50.1, 41.3, 41.1, 39.9, 39.4, 35.5, 35.4, 29.6, 29.5, 22.4, 16.4 (**Supplementary Figure S34**).

*Synthesis of disubstituted, “short” Ir(III) conjugate Co-3.* Pyridotriazolyl triazine 12 (30.0 mg, 0.056 mmol, 2 eq) and [Ir- phenylpyridine (ppy)_2_Cl]_2_ (30.0 mg, 0.028 mmol, 1 eq) were dissolved in dry, degassed 3:2 DCM/MeOH (10 mL). The mixture was stirred under an argon atmosphere at room temperature, monitored by TLC (eluent mixture: 9:1 DCM/MeOH, developed in molybdic reagent) for 24 hours. After reaction completion, the solvent was removed under reduced pressure and the crude was purified by flash chromatography (eluent mixture: 9:1 DCM/MeOH), affording 48.5 mg of pure target “short” conjugate Co-3 as a yellow solid (0.0453 mmol, 81% yield). HPLC Purity >93%. MS (ESI^+^): *m/z* 1036.35 [M]^+^, calculated for C_51_H_45_IrN_11_O_2_ 1036.34. ^1^H NMR (400 MHz, CDCl_3_) some signals marked as * in the text are split due to the presence of two atropoisomers in an undetermined ratio. δ: 10.41*, 10.27* (2s, 1H, H triazole), 9.08*, 8.99* (2s, 1H, H triazine), 8.67-8.28 (m, 2H, H phenyl-pyridine), 7.92-6.25 (m, 26H, 8H biphenyl, 18H phenyl-pyridine), 5.39*, 5.38* (2s, 2H, OCH_2_), 5.29-5.19 (m, 2H, NH), 4.80-4.74 (CH_2_- triazole), 3.65-3.63 (m, 2H, NHCH_2_), 3.48-3.43 (m, 2H, NHCH_2_), 3.20-3.18 (COCH_2_), 2.25*, 2.17* (2s, 3H, CH_3_). ^13^C NMR (101 MHz, CDCl_3_) some signals are split due to the presence of two atropoisomers in an undetermined ratio. δ: 169.5, 168.6, 167.6, 150.1, 149.9, 149.7, 149.6, 148.3, 146.2, 143.9, 143.6, 143.0, 142.8, 142.1, 139.5, 138.0, 134.8, 134.7, 132.0, 131.8, 131.7, 130.8, 130.4, 130.3, 129.5, 129.4, 129.3, 128.9, 128.2, 127.0, 126.0, 125.9, 125.6, 124.7, 124.5, 123.5, 123.4, 122.9, 122.8, 122.4, 119.8, 119.4, 67.8, 48.7, 40.0, 39.8, 39.5, 34.5, 34.2, 31.7, 22.8, 16.5, 16.4, 14.3 (**Supplementary Figure S35**). UV-vis (DCM, r.t.) λ_max_: 254 nm (ε = 5.32∙10^5^), 382 nm (ε = 5.29∙10^3^). Fluorescence Emission (DCM nitrogen degassed, r.t.) λ_em_: 478 nm φ_em_: 0.53, τ: 2.09 μs, Fluorescence Emission (2-MeTHF, 77K) λ_em_: 470 nm, τ: 4.62 μs (**Supplementary Table S2**).

*Synthesis of disubstituted, “long” Ir(III) conjugate Co-4.* Pyridotriazolyl triazine 13 (31.0 mg, 0.056 mmol, 2 eq) and the complex precursor [Ir(ppy)_2_Cl]_2_ (30.0 mg, 0.028 mmol, 1 eq) were dissolved in dry, degassed 3:2 DCM/MeOH (10 mL). The mixture was stirred under an argon atmosphere at room temperature, monitored by TLC (eluent mixture: 9:1 DCM/MeOH, developed in molybdic reagent) for 24 hours. After reaction completion, the solvent was removed under reduced pressure and the crude was purified by flash chromatography (eluent mixture: 9:1 DCM/MeOH) affording 47.2 mg of pure target “long” conjugate Co-4 as a yellow solid (0.0430 mmol, 77% yield). HPLC Purity >93%. MS (ESI^+^): *m/z* 1064.42 [M]^+^, calculated for C_53_H_49_IrN_11_O_2_ 1064.37. ^1^H NMR (400 MHz, CDCl_3_) some signals marked as * in the text are split due to the presence of two atropoisomers in an undetermined ratio. δ: 10.57 (s, 1H, H triazole), 8.78-8.59 (m, 2H, H triazine, H phenyl-pyridine), 7.98-6.27 (m, 27H, 8H biphenyl, 19H phenyl-pyridine), 5.42*, 5.36* (2s, 2H, OCH_2_), 4.60-4.56 (m, 2H, CH_2_-triazole), 3.71-3.68 (m, 2H, NHCH_2_), 3.52-3.50 (m, 2H, NHCH_2_), 2.46-2.42 (m, 2H, COCH_2_), 2.25*, 2.19* (2s, 3H, CH_3_), 1.97-1.94 (m, 2H, CH_2_), 1.71-1.68 (m, 2H, CH_2_). ^13^C NMR (101 MHz, CDCl_3_) some signals are split due to the presence of two atropoisomers in an undetermined ratio. δ: 173.7, 168.5, 167.8, 150.1, 150.0, 148.6, 148.2, 146.3, 143.8, 143.7, 138.1, 132.0, 131.9, 130.9, 130.8, 130.3, 129.5, 129.2, 128.9, 128.2, 127.1, 127.0, 125.9, 125.6, 124.8, 124.5, 123.4, 122.8, 122.4, 119.6, 68.5, 51.3, 45.7, 40.7, 38.7, 35.1, 28.5, 22.5, 16.5, 16.2 (**Supplementary Figure S36**). UV-vis (DCM, r.t.) λ_max_: 253 nm (ε = 5.41∙10^4^), 384 nm (ε = 5.19∙10^3^). Fluorescence Emission (DCM nitrogen degassed, r.t.), λ_em_: 478 nm, φ_em_: 0.55, τ: 2.36 μs, Fluorescence Emission (2-MeTHF, 77K), λ_em_: 470 nm, τ: 4.55 μs (**Supplementary Table S2**).

*Synthesis of N-(2-azidoethyl)-4-chloro-6-((2-methyl-[1,1'-biphenyl]-3-yl)methoxy)-1,3,5-triazin-2-amine 15.* A solution of 2-azidoethan-1-amine hydrochloride (53.7 mg, 0.433 mmol, 1 eq) and DIPEA (0.226 mL, 1.30 mmol, 3 eq) in dry DCM (2.3 mL) was added to a solution of dichloro triazine 14 (150.0 mg, 0.433 mmol, 1 eq) in dry DCM (2.0 mL) at -20 °C, under a nitrogen atmosphere. The reaction mixture was slowly warmed to room temperature, monitored by TLC (eluent mixture: 6:4 DCM/*n*-hexane, developed in molybdic reagent) and stirred for 6 hours. After reaction completion, a saturated aqueous solution of NH_4_Cl (5 mL) was slowly added until neutral pH. The mixture was extracted with DCM (10 mL) and the collected organic phases were washed with brine (20 mL), dried over sodium sulfate, filtered, and evaporated under reduced pressure. The crude was purified by flash chromatography over silica gel (eluent mixture: 7:3 *n*-hexane/EtOAc) to afford 155.2 mg of pure target chloroalkynyl triazine amine 15 as a white solid (0.392 mmol, 91% yield). MS (ESI^+^): *m/z* 396.38 [M+H]^+^, calculated for C_19_H_18_ClN_7_O 395.13. ^1^H NMR (400 MHz, CDCl_3_) some signals marked as * in the text are split due to the presence of two atropoisomers in an undetermined ratio. δ: 7.44-7.33 (m, 4H, H biphenyl), 7.30-7.23 (m, 4H, H biphenyl), 6.48*, 6.27* (2m, 1H, NH), 5.50*, 5.46* (2s, 2H, OCH_2_), 3.67-3.62 (m, 2H, NHCH_2_), 3.55-3.48 (m, 2H, N_3_CH_2_), 2.28*, 2.26* (2s, 3H, CH_3_). ^13^C NMR (101 MHz, CDCl_3_) some signals are split due to the presence of two atropoisomers in an undetermined ratio. δ: 170.9, 170.8, 167.3, 143.2, 141.9, 135.0, 134.8, 133.8, 130.8, 130.7, 129.5, 129.0, 128.8, 128.3, 127.1, 125.7, 69.3, 69.0, 50.7, 50.4, 40.6, 40.5, 16.5, 16.4 (**Supplementary Figure S37**).

*Synthesis of N-(6-azidohexyl)-4-chloro-6-((2-methyl-[1,1'-biphenyl]-3-yl)methoxy)-1,3,5-triazin-2-amine 16.* Target compound 16 was synthesized according to the procedure reported for 15, starting from dichloro triazine 14 (150.0 mg, 0.433 mmol, 1 eq), 6-azidohexyl-1-amine (61.6 mg, 0.433 mmol, 1 eq) and DIPEA (0.098 mL, 0.563 mmol, 1.3 eq) in dry DCM (4.3 mL), from -20°C to room temperature for 6 hours, monitoring by TLC (eluent mixture: 6:4 DCM/*n*-hexane, developed in molybdic reagent). The crude was purified by flash chromatography over silica gel (eluent mixture: 8:2 *n*-hexane/EtOAc) to afford 168.5 mg of pure target chloroalkynyl triazine amine 16 as a white solid (0.373 mmol, 86% yield). MS (ESI^+^): *m/z* 452.40 [M+H]^+^, calculated for C_23_H_26_ClN_7_O 451.19. ^1^H NMR (400 MHz, CDCl_3_) some signals marked as * in the text are split due to the presence of two atropoisomers in an undetermined ratio. δ: 7.44-7.33 (m, 4H, H biphenyl), 7.30-7.23 (m, 4H, H biphenyl), 5.92-5.87 (m, 1H, NH), 5.49*, 5.45* (2s, 2H, OCH_2_), 3.50-3.44 (m, 2H, NHCH_2_), 3.29-3.24 (m, 2H, N_3_CH_2_), 2.28*, 2.26* (2s, 3H, CH_3_), 1.64-1.56 (m, 4H, 2 CH_2_), 1.45-1.37 (m, 4H, 2 CH_2_). ^13^C NMR (101 MHz, CDCl_3_) some signals are split due to the presence of two atropoisomers in an undetermined ratio. δ: 170.9, 167.1, 143.1, 142.0, 141.9, 135.0, 134.8, 134.0, 133.8, 127.1, 127.0, 125.7, 125.6, 69.1, 69.0, 51.5, 51.4, 41.3, 29.4, 29.2, 28.9, 26.5, 26.4, 16.5, 16.4 (**Supplementary Figure S38**).

*Synthesis of N-(2-((4-((2-azidoethyl)amino)-6-((2-methyl-[1,1'-biphenyl]-3-yl)methoxy)-1,3,5-triazin-2-yl)amino)ethyl) acetamide 17.* A solution of *N*-(2-aminoethyl)-acetamide (39.7 mg, 0.388 mmol, 1.1 eq) and DIPEA (0.080 mL, 0.459 mmol, 1.3 eq) in dry CH_3_CN (2.0 mL) was added to a solution of chloroalkynyl triazine amine 15 (139.7 mg, 0.353 mmol, 1 eq) in dry CH_3_CN (1.5 mL) under nitrogen atmosphere. The reaction mixture was heated at 70°C and stirred for 7 hours monitoring by TLC (eluent mixture: 95:5 DCM/MeOH, developed in molybdic reagent). After reaction completion, it was cooled to room temperature and the solvent was evaporated under reduced pressure. DCM (5 mL) was added to the residue and a saturated aqueous solution of NH_4_Cl (5 mL) was slowly added until neutral pH. Then, the mixture was extracted with DCM (5 mL) and the collected organic phases were washed with brine (20 mL), dried over sodium sulfate, filtered, and evaporated under reduced pressure.The crude was purified by flash chromatography over silica gel (eluent mixture: 95:5 DCM/MeOH) to afford 94.0 mg of pure target azidoaniline 17 as a white solid (0.204 mmol, 58% yield). MS (ESI^+^): *m/z* 462.42 [M+H]^+^ , calculated for C_23_H_27_N_9_O_2_ 461.23. ^1^H NMR (400 MHz, CDCl_3_) some signals marked as * in the text are split due to the presence of two atropoisomers in an undetermined ratio. δ: 7.43-7.32 (m, 4H, H biphenyl), 7.29-7.20 (m, 4H, H biphenyl), 6.27-5.70 (m, 2H, NH), 5.40* (m, 2H, OCH_2_), 3.61-3.42 (m, 8H, 4 CH_2_), 2.26 (bs, 3H, CH_3_ biphenyl), 1.93 (bs, 3H, COCH_3_). ^13^C NMR (101 MHz, CDCl_3_) some signals are split due to the presence of two atropoisomers in an undetermined ratio. δ: 171.1, 143.1, 141.9, 134.7, 134.3, 130.5, 129.5, 129.4, 128.8, 128.2, 127.0, 125.6, 50.8, 40.9, 40.4, 39.8, 23.3, 16.4 (**Supplementary Figure S39**).

*Synthesis of N-(2-((4-((6-azidohexyl)amino)-6-((2-methyl-[1,1'-biphenyl]-3-yl)methoxy)-1,3,5-triazin-2-yl)amino)ethyl) acetamide 18.* Target compound 18 was synthesized according to the procedure reported for 17, starting from chloroalkynyl triazine amine 16 (156.4 mg, 0.346 mmol, 1 eq), *N*-(2-aminoethyl)-acetamide (38.9 mg, 0.381 mmol, 1.1 eq) and DIPEA (0.080 mL, 0.450 mmol, 1.3 eq) in dry CH_3_CN (1.70 mL) at 70°C for 7 hours, monitoring by TLC (eluent mixture: 95:5 DCM/MeOH, developed in molybdic reagent). The crude was purified by flash chromatography over silica gel (eluent mixture: 95:5 DCM/MeOH) to afford 158.5 mg of pure target azidoaniline 18 as a white solid (0.306 mmol, 88% yield). MS (ESI^+^): *m/z* 518.50 [M+H]^+^, calculated for C_27_H_35_N_9_O_2_ 517.29. ^1^H NMR (400 MHz, CDCl_3_) some signals marked as * in the text are split due to the presence of two atropoisomers in an undetermined ratio. δ: 7.43-32 (m, 4H, H biphenyl), 7.29-7.22 (m, 4H, H biphenyl), 7.08-5.69 (m, 3H, NH), 5.48*, 5.39* (2bs, 2H, OCH_2_), 3.60-3.55 (m, 2H, NHCH_2_), 3.45-3.42 (m, 4H, 2 NHCH_2_), 3.27-3.24 (m, 2H, N_3_CH_2_), 2.26 (bs, 3H, CH_3_ biphenyl), 1.95 (bs, 3H, OCH_3_), 1.60 (m, 4H, 2 CH_2_), 1.39 (m, 4H, 2 CH_2_). ^13^C NMR (101 MHz, CDCl_3_) some signals are split due to the presence of two atropoisomers in an undetermined ratio. δ: 170.9, 170.4, 165.1, 164.4, 143.3, 141.8, 134.3, 130.4, 129.3, 128.8, 128.1, 126.9, 125.5, 125.4, 51.3, 41.0, 40.6, 39.5, 38.8, 29.3, 28.7, 26.4, 22.9, 16.3. (**Supplementary Figure S40**)

*Synthesis of N-(2-((4-((2-methyl-[1,1'-biphenyl]-3-yl) methoxy)-6-((2-(4-phenyl-1H-1,2,3-triazol-1-yl)ethyl)amino)-1,3,5-triazin-2-yl)amino)ethyl)acetamide 19.* Target compound 19 was synthesized according to the procedure reported for 12, starting from azidoaniline 17 (86.3 mg, 0.187 mmol, 1 eq), 2-ethynylpyridine (19.3 mg, 0.187 mmol, 1 eq), 0.15 mol/L CuSO_4_∙5H_2_O (0.125 mL) and 0.5 mol/L Na ascorbate (0.094 mL) in 1:1 THF/H_2_O (1.9 mL), monitoring by TLC (eluent mixture: 95:5 DCM/MeOH, developed in molybdic reagent). The crude was purified by Biotage^TM^ direct phase chromatography (eluent mixture: DCM/MeOH, gradient from 0% to 10% MeOH) to afford 89.6 mg of pure target pyridotriazolyl triazine 19 as a white solid (0.159 mmol, 85% yield). MS (ESI^+^): *m/z* 564.27 [M+H]^+^, calculated for C_31_H_33_N_9_O_2_ 563.28. ^1^H NMR (400 MHz, CDCl_3_) some signals marked as * in the text are split due to the presence of two atropoisomers in an undetermined ratio. δ: 8.53-8.52 (m, 1H, H pyridine), 8.16-8.09 (m, 2H, H triazole, H pyridine), 7.77-7.73 (m, 1H, H pyridine), 7.42-7.33 (m, 4H, H biphenyl), 7.27-7.17 (m, 5H, H biphenyl, H pyridine), 7.12*, 6.95*, 6.61*, 6.44*, 6.27*, 6.18* (6m, 3H, NH), 5.37*, 5.29* (2s, 2H, OCH_2_), 4.57-4.52 (m, 2H, CH_2_-triazle), 3.89-3.84 (m. 2H, NHCH_2_), 3.52-3.49 (m, 2H, NHCH_2_), 3.41-3.39 (m, 2H, NHCH_2_), 2.25*, 2.22* (2s, 3H, CH_3_ biphenyl), 1.96*, 1.92* (2s, 3H, OCH_3_). ^13^C NMR (101 MHz, CDCl_3_) some signals are split due to the presence of two atropoisomers in an undetermined ratio. δ: 171.1, 167.3, 150.2, 149.7, 143.0, 141.7, 138.0, 135.1, 134.5, 130.3, 129.5, 128.5, 128.2, 127.0, 125.6, 123.1, 120.3, 67.3, 50.0, 40.9, 40.6, 39.9, 22.8, 16.0 (**Supplementary Figure S41**).

*Synthesis of N-(2-((4-((2-methyl-[1,1'-biphenyl]-3-yl) methoxy)-6-((6-(4-phenyl-1H-1,2,3-triazol-1-yl)hexyl)amino)-1,3,5-triazin-2-yl)amino)ethyl)acetamide 20.* Target compound 20 was synthesized according to the procedure reported for compound 12, starting from azidoaniline 18 (121.6 mg, 0.0.235 mmol, 1 eq), 2-ethynylpyridine (24.2 mg, 0.235 mmol, 1 eq), 0.15 mol/L CuSO_4_∙5H_2_O (0.157 mL) and 0.5 mol/L Na ascorbate solution (0.118 mL) in 1:1 THF/H_2_O (2.4 mL), monitoring by TLC (eluent mixture: 95:5 DCM/MeOH, developed in molybdic reagent). The crude was purified by Biotage^TM^ direct phase chromatography (eluent mixture: DCM/MeOH, gradient from 0% to 20% MeOH) to afford 139.2 mg of pure target pyridotriazolyl triazine 20 as a white solid (0.224 mmol, 95% yield). MS (ESI^+^): *m/z* 620.35 [M+H]^+^, calculated for C_35_H_41_N_9_O_2_ 619.34. ^1^H NMR (400 MHz, CDCl_3_) some signals marked as * in the text are split due to the presence of two atropoisomers in an undetermined ratio. δ: 8.58-8.57 (m, 1H, H pyridine), 8.23-8.09 (m, 2H, H triazole, H pyridine), 7.80-7.76 (m, 1H, H pyridine), 7.42-7.31 (m, 4H, H biphenyl), 7.28-7.19 (m, 5H, H biphenyl, H pyridine), 6.68*, 6.45*, 6.32*, 5.79*, 5.69*, 5.62* (6m, 3H, NH), 5.44-5.34 (m, 2H, OCH_2_), 4.43-4.41 (m, 2H, CH_2_- triazole), 3.56-3.40 (m, 6H, 3 NHCH_2_), 2.25 (s, 3H, CH_3_ biphenyl), 1.97-1.91 (m, 5H, COCH_3_, CH_2_) 1.55 (m, 2H, CH_2_), 1.41-1.36 (m, 4H, 2 CH_2_). ^13^C NMR (101 MHz, CDCl_3_) some signals are split due to the presence of two atropoisomers in an undetermined ratio. δ: 171.2, 170.1, 150.2, 149.4, 143.0, 141.9, 137.2, 134.5, 130.6, 129.4, 128.8, 128.5, 128.2, 127.0, 125.5, 123.6, 123.0, 120.4, 119.9, 67.7, 50.4, 40.8, 39.8, 30.1, 29.3, 26.1, 23.3, 16.4 (**Supplementary Figure S42**).

*Synthesis of trisubstituted, “short” Ir(III) conjugate Co-5.* Pyridyltriazolyl aniline 19 (31.0 mg, 0.056 mmol, 2 eq) and [Ir(ppy)_2_Cl]_2_ (30.0 mg, 0.028 mmol, 1 eq) were dissolved in dry, degassed 3:2 DCM/MeOH (10 mL). The mixture was stirred at room temperature under an argon atmosphere, monitored by TLC (eluent mixture: 9:1 DCM/MeOH, developed in molybdic reagent) for 24 hours. After reaction completion, the solvent was removed under reduced pressure and the crude was purified by flash chromatography (eluent mixture: 9:1 DCM/MeOH) affording 41.7 mg of pure target conjugate Co-5 as a yellow solid (0.0380 mmol, 68% yield). HPLC Purity >95%. MS (ESI^+^): *m/z* 1065.77 [M]^+^, calculated for C_52_H_48_IrN_12_O_2_ 1065.37. ^1^H NMR (400 MHz, CDCl_3_) some signals marked as * in the text are split due to the presence of two atropoisomers in an undetermined ratio. δ: 10.92-10.58 (m, 1H, H triazole), 9.25-8.10 (m, 3H, NH), 8.01-6.26 (m, 28H, 8 H biphenyl, 20 H phenyl-pyridine), 5.35*, 5.29* (2s, 2H, OCH_2_), 4.69-4.65 (m, 2H, CH_2_- triazole), 3.94-3.69 (m, 2H, NHCH_2_), 3.56-3.45 (m, 4H, 2 NHCH_2_), 2.21 (s, 3H, CH_3_ biphenyl), 2.11 (s, 3H, COCH_3_). ^13^C NMR (101 MHz, CDCl_3_) some signals are split due to the presence of two atropoisomers in an undetermined ratio. δ: 171.3, 170.6, 168.5, 167.8, 167.2, 167.0, 150.3, 150.0, 149.8, 148.7, 146.5, 143.8, 143.7, 143.6, 142.8, 142.2, 139.7, 138.1, 130.0, 135.3, 134.6, 132.0, 131.8, 130.8, 130.2, 130.1, 129.5, 128.8, 128.2, 126.9, 126.1, 125.5, 125.4, 124.8, 124.5, 123.3, 122.8, 122.4, 119.6, 67.2, 50.6, 41.2, 39.3, 23.5, 16.4 (**Supplementary Figure S43**). UV-vis (DCM, r.t.) λ_max_: 252 nm (ε = 5.25∙10^5^), 383 nm (ε = 5.39∙10^3^). Emission (DCM nitrogen degassed, r.t.), λ_em_: 478 nm, φ_em_: 0.63, τ: 2.69 μs, Emission (2-MeTHF, 77K) λ_em_: 470 nm, τ: 4.50 μs (**Supplementary Table S2**).

*Synthesis of trisubstituted, “long” Ir(III) conjugate Co-6.* Pyridyltriazolyl aniline 20 (34.8 mg, 0.056 mmol, 2 eq) and [Ir(ppy)_2_Cl]_2_ (30.0 mg, 0.028 mmol, 1 eq) were dissolved in dry, degassed 3:2 DCM/MeOH (10 mL). The mixture was stirred under an argon atmosphere, monitored by TLC (eluent mixture: 9:1 DCM/MeOH, developed in molybdic reagent) for 24 hours. After reaction completion, the solvent was removed under reduced pressure and the crude was purified by flash chromatography (eluent mixture: 9:1 DCM/MeOH), affording 46.4 mg of pure target conjugate Co-6 as a yellow solid (0.040 mmol, 72% yield). HPLC Purity >93%. MS (ESI^+^): *m/z* 1122.00 [M]^+^, calculated for C_56_H_56_IrN_12_O_2_ 1121.43. ^1^H NMR (400 MHz, CDCl_3_) some signals marked as *in the text are split due to the presence of two atropoisomers in an undetermined ratio. δ: 10.74 (s, 1H, H triazole), 9.02-9.00 (m, 1H, pyridine), 7.98-6.29 (m, 27H, 8 H biphenyl, 19 H phenyl-pyridine), 5.38*, 5.33* (2s, 2H, OCH_2_), 4.52-4.45 (m, 2H, CH_2_- triazole), 3.64-3.20 (m, 6H, 3 NHCH_2_), 2.25 (s, 3H, CH_3_ biphenyl), 2.03-1.96 (m, 5H, COCH_3_, CH_2_) 1.83-1.76 (m, 4H, 2 CH_2_), 1.54-1.47 (m, 2H, CH_2_). ^13^C NMR (101 MHz, CDCl_3_) some signals are split due to the presence of two atropoisomers in an undetermined ratio. δ: 169.6, 168.5, 167.8, 149.8, 149.3, 148.4, 146.5, 143.9, 143.8, 143.7, 142.8, 142.2, 138.1, 137.9, 135.3, 131.9, 130.8, 130.2, 130.0, 129.5, 128.8, 128.2, 128.1, 126.9, 126.8, 126.0, 125.5, 125.4, 124.8, 124.4, 123.3, 122.9, 122.8, 122.3, 119.6, 119.5, 70.7, 70.4, 70.2, 70.1, 67.8, 67.1, 52.2, 40.5, 29.7, 29.4,, 25.8, 23.3, 16.4 (**Supplementary Figure S44**). UV-vis (DCM, r.t.) λ_max_: 253 nm (ε = 4.79∙10^5^), 386 nm (ε = 5.00∙10^3^). Emission (DCM nitrogen degassed, r.t.), λ_em_: 478 nm, φ_em_: 0.49, τ: 2.10 μs. Emission (2-MeTHF, 77K), λ_em_: 470 nm, τ: 4.51 μs (**Supplementary Table S2**).

^
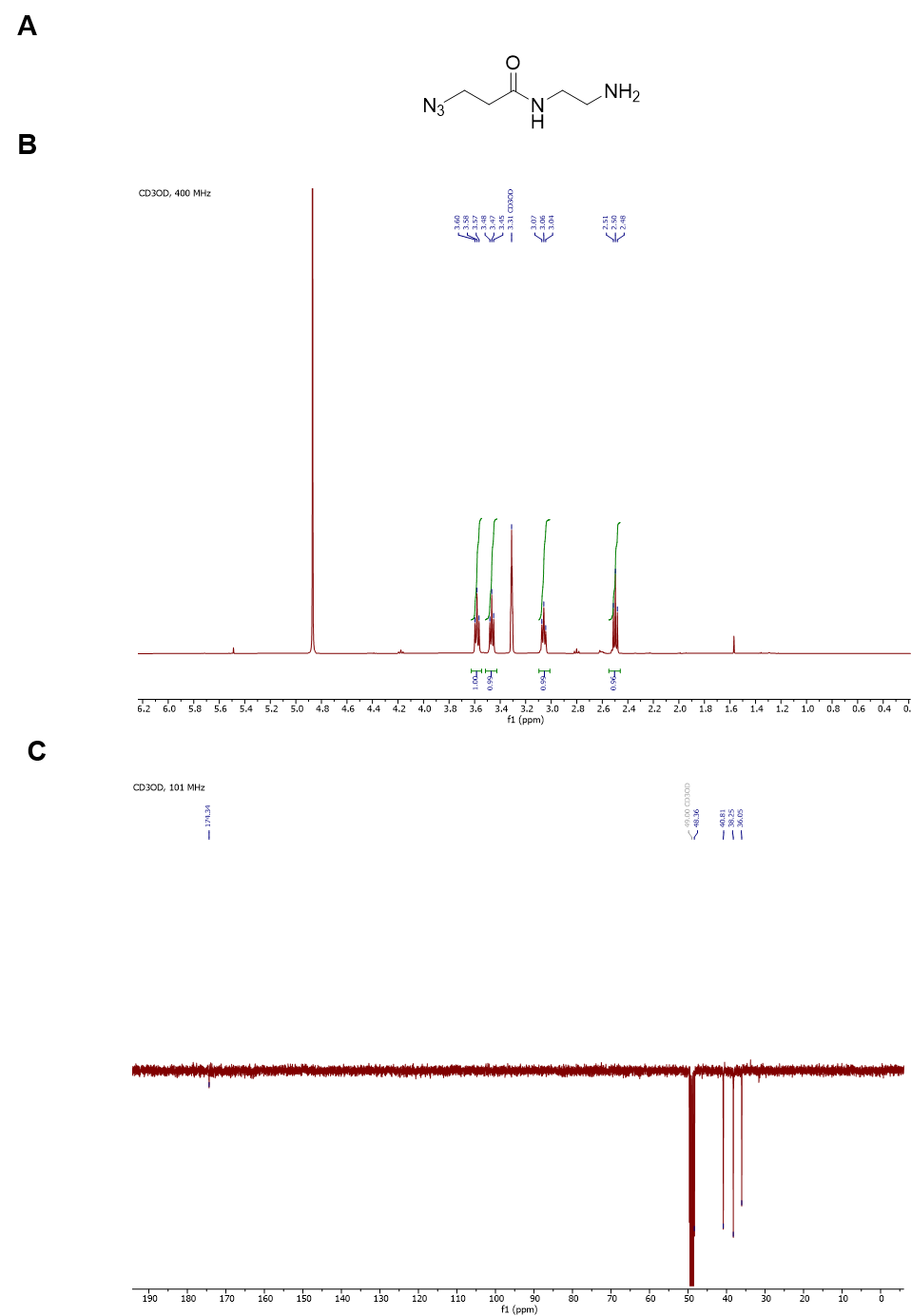
^

**Supplementary Figure S29. Spectroscopic characterization of the compound of tert-butyl (2-(3-azidopropanamido)ethyl) carbamate 8**. **(A)** Chemical structure. **(B)** ^1^H NMR (400 MHz, methanol-d_4_). **(C)** ^13^C NMR (101 MHz, methanol-d_4_).


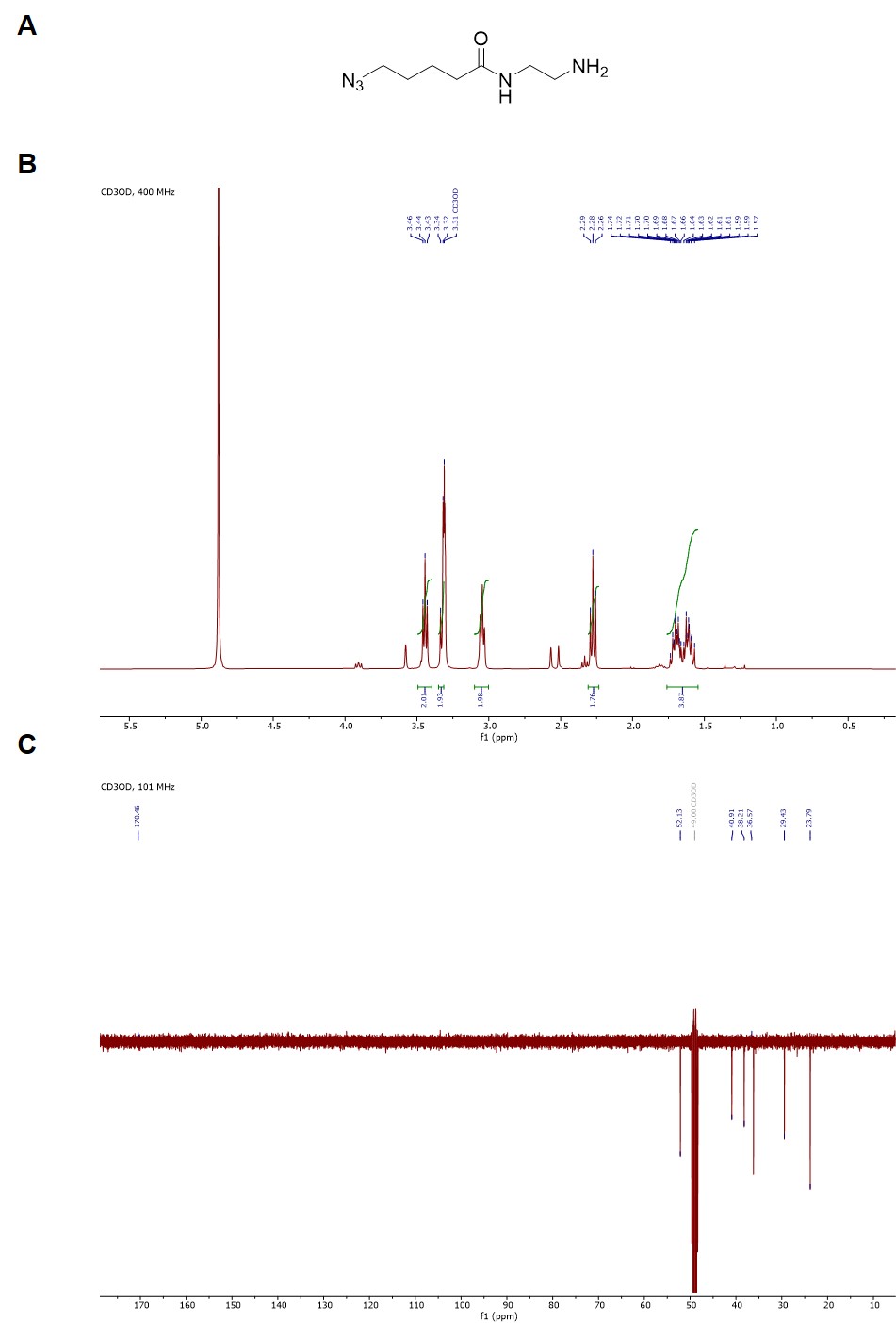


**Supplementary Figure S30. Spectroscopic characterization of tert-butyl (2-(5-azidopentanamido)ethyl) carbamate 9**. **(A)** Chemical structure. **(B)** ^1^H NMR (400 MHz, methanol-d_4_). (C) ^13^C NMR (101 MHz, methanol-d_4_).


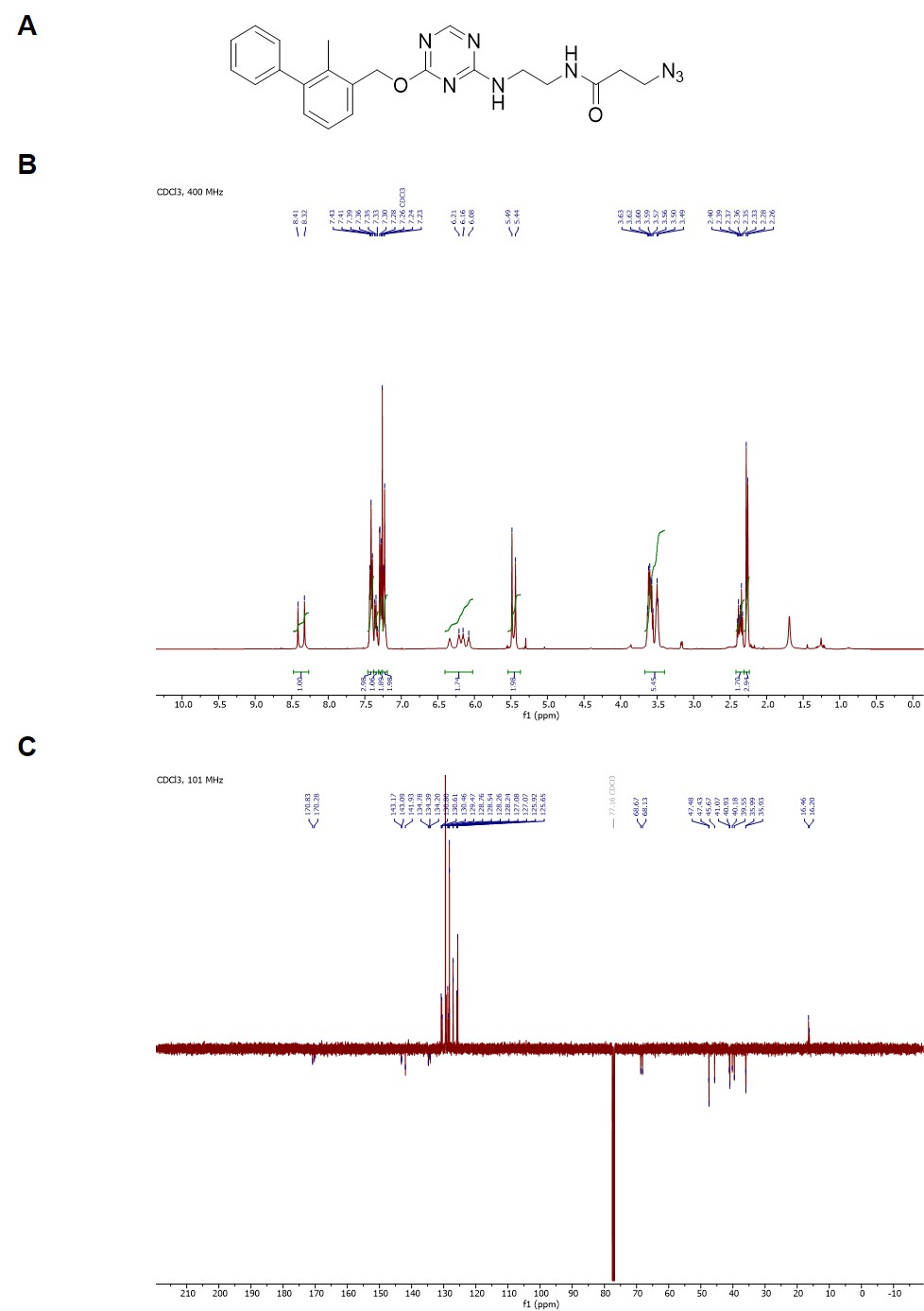


**Supplementary Figure S31. Spectroscopic characterization of 3-azido-N-(2-((4-((2-methyl-[1,1'-biphenyl]-3-yl)methoxy)-1,3,5-triazin-2-yl)amino)ethyl) propanamide 10**. **(A)** Chemical structure. **(B)** ^1^H NMR (400 MHz, CDCl_3_). (C) ^13^C NMR (101 MHz, CDCl_3_).


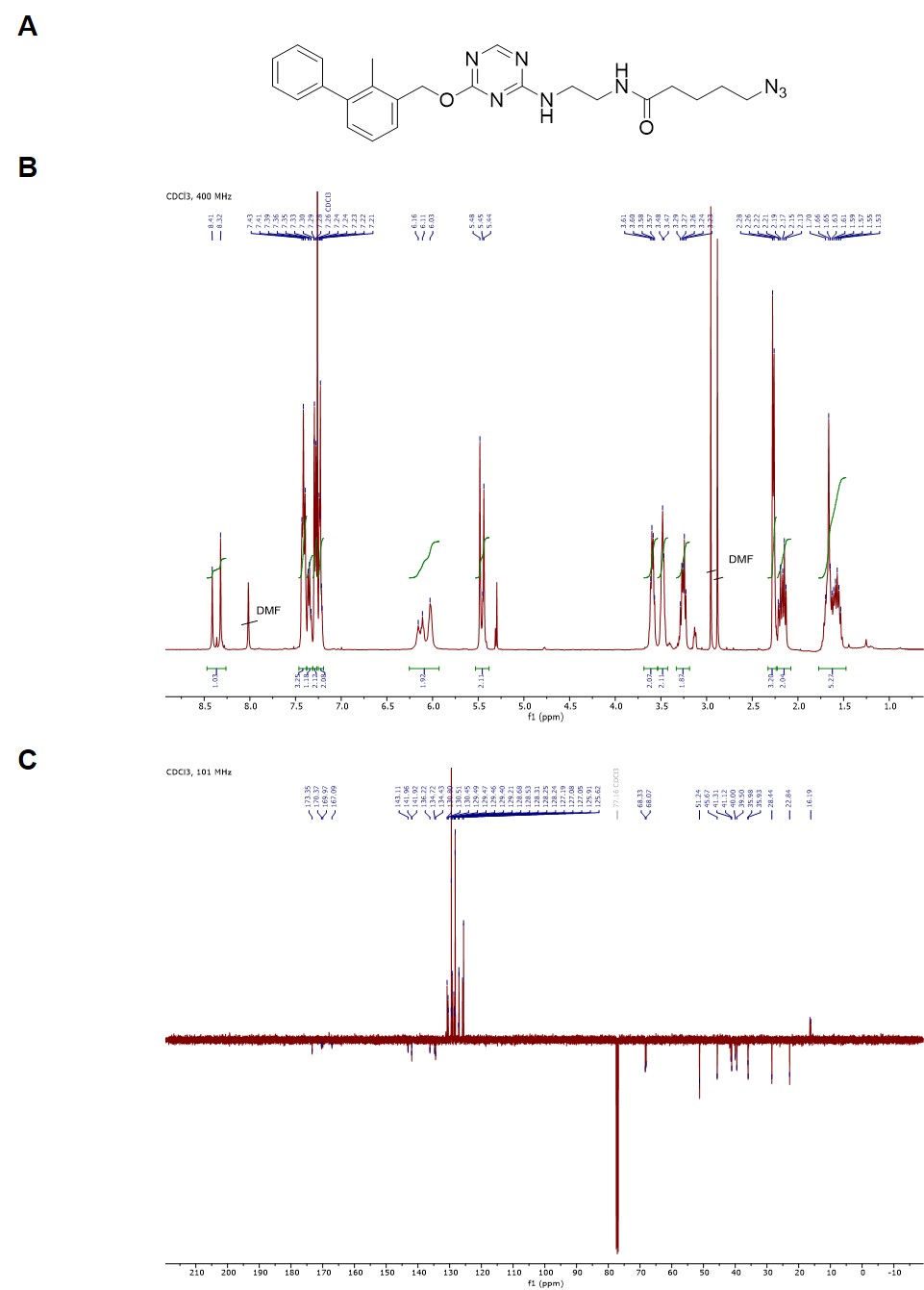


**Supplementary Figure S32. Spectroscopic characterization of 5-azido-N-(2-((4-((2-methyl-[1,1'-biphenyl]-3-yl)methoxy)-1,3,5-triazin-2-yl)amino)ethyl) pentanamide 11**. **(A)** Chemical structure. **(B)** ^1^H NMR (400 MHz, CDCl_3_). (C) ^13^C NMR (101 MHz, CDCl_3_).


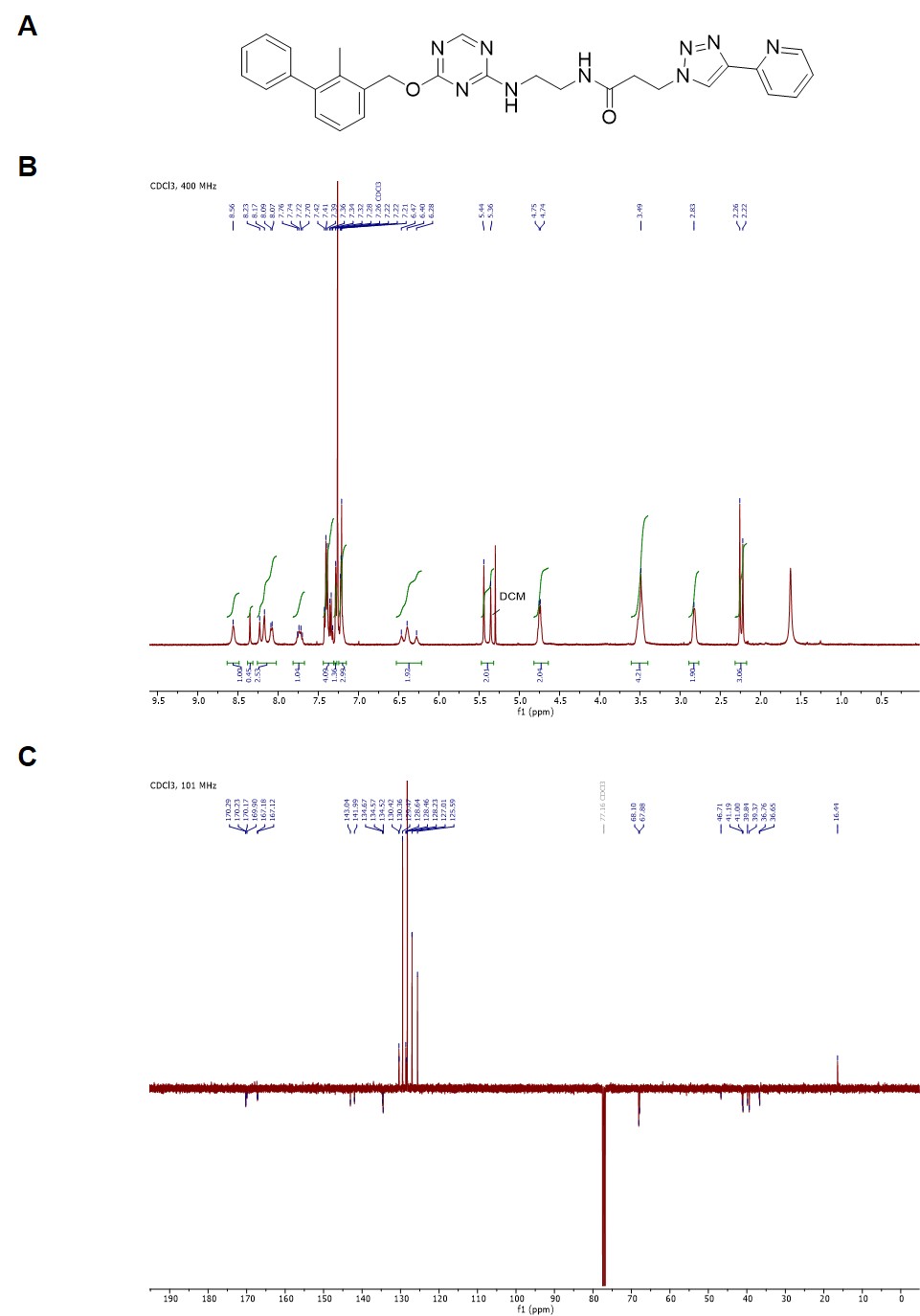


**Supplementary Figure S33. Spectroscopic characterization of N-(2-((4-((2-methyl-[1,1'-biphenyl]-3-yl)methoxy)-1,3,5-triazin-2-yl)amino)ethyl)-3-(4-phenyl-1H-1,2,3-triazol-1-yl) propenamide 12**. **(A)** Chemical structure. **(B)** ^1^H NMR (400 MHz, CDCl_3_). (C) ^13^C NMR (101 MHz, CDCl_3_).


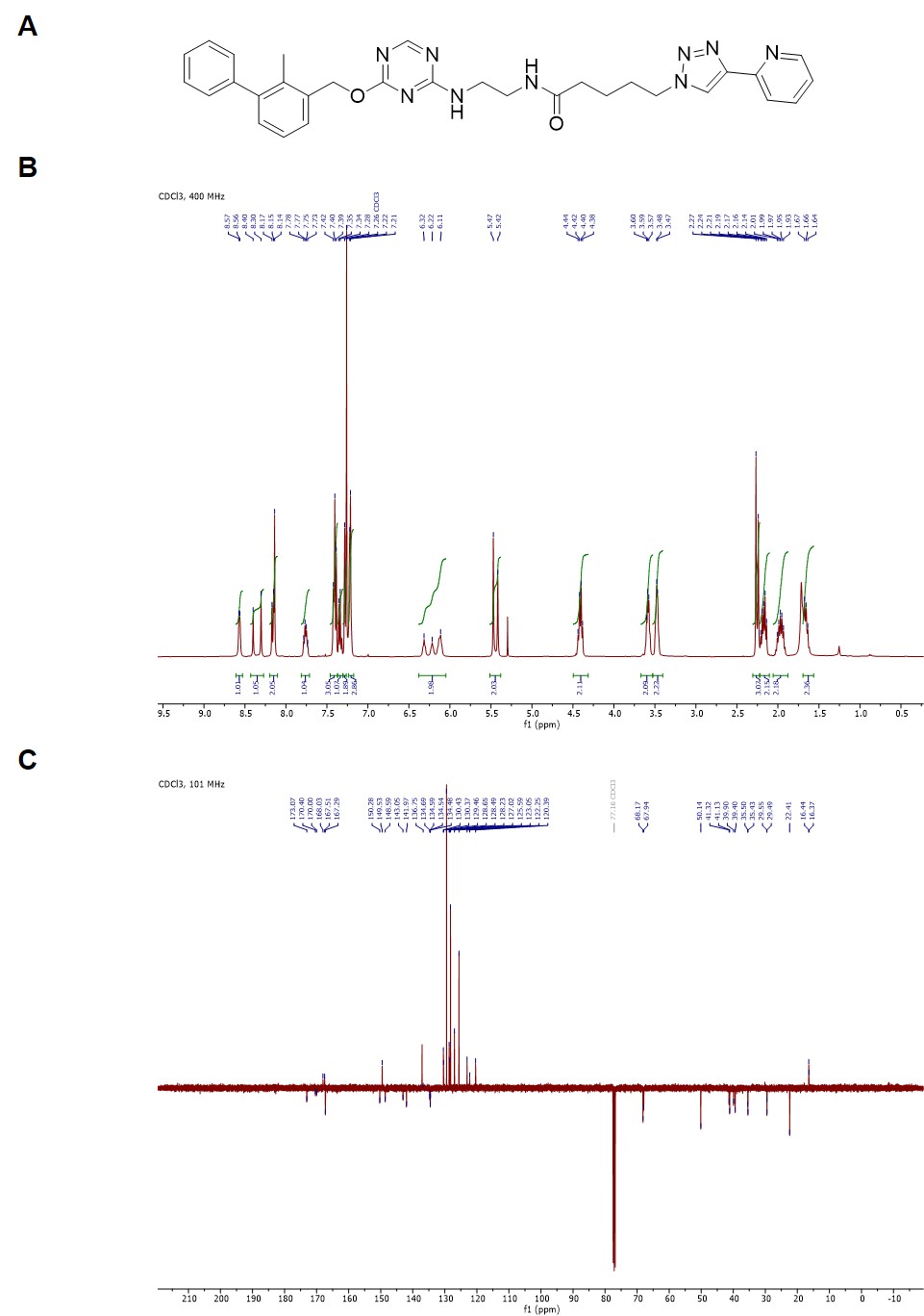


**Supplementary Figure S34. Spectroscopic characterization of N-(2-((4-((2-methyl-[1,1'-biphenyl]-3-yl)methoxy) -1,3,5-triazin-2-yl)amino)ethyl)-5-(4-(pyridin-2-yl)-1H-1,2,3-triazol-1-yl)pentanamide 13**. **(A)** Chemical structure. **(B)** ^1^H NMR (400 MHz, CDCl_3_). (C) ^13^C NMR (101 MHz, CDCl_3_).


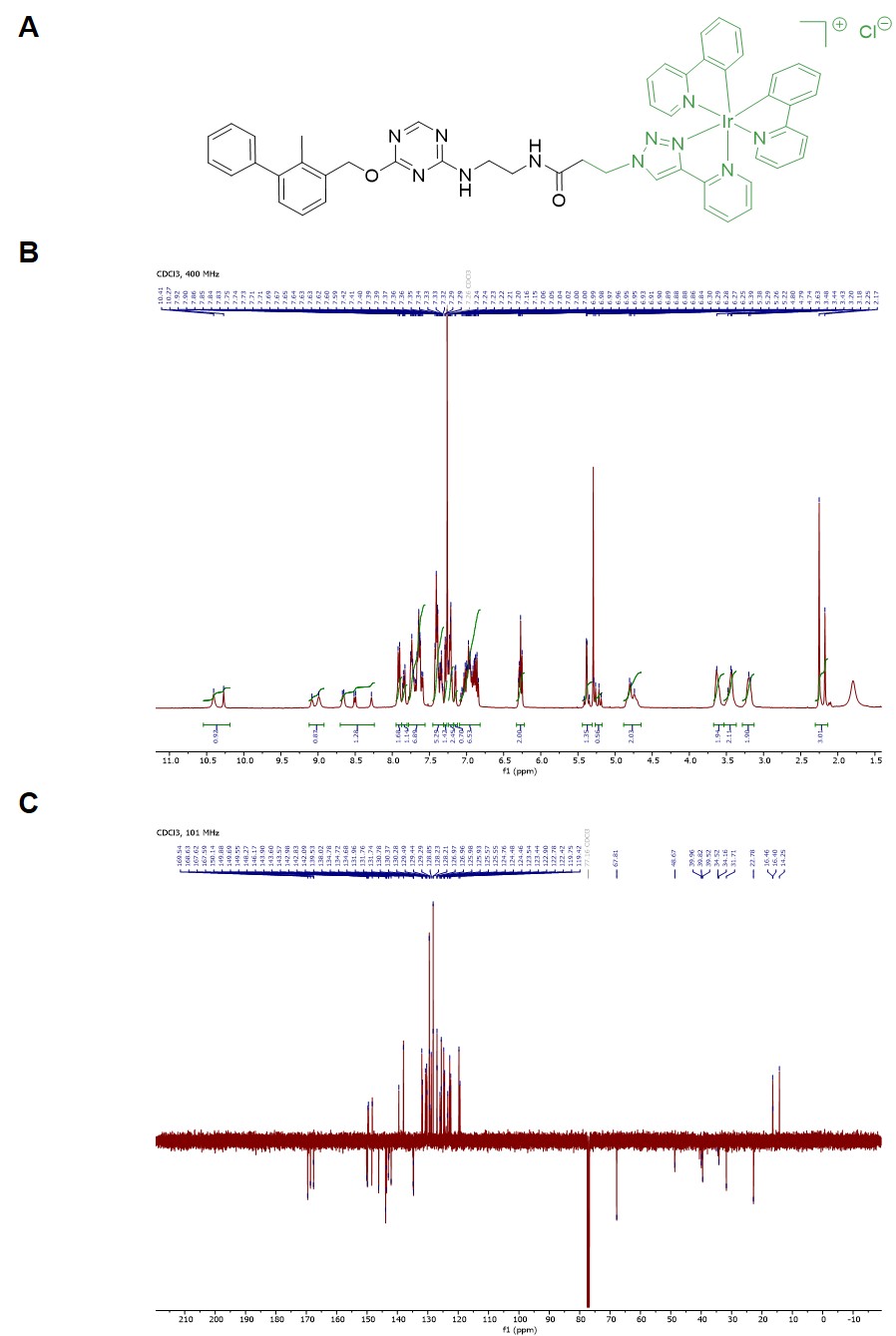


**Supplementary Figure S35. Spectroscopic characterization of disubstituted, “short” Ir(III) complex Co-3**. **(A)** Chemical structure. **(B)** ^1^H NMR (400 MHz, CDCl_3_). (C) ^13^C NMR (101 MHz, CDCl_3_).


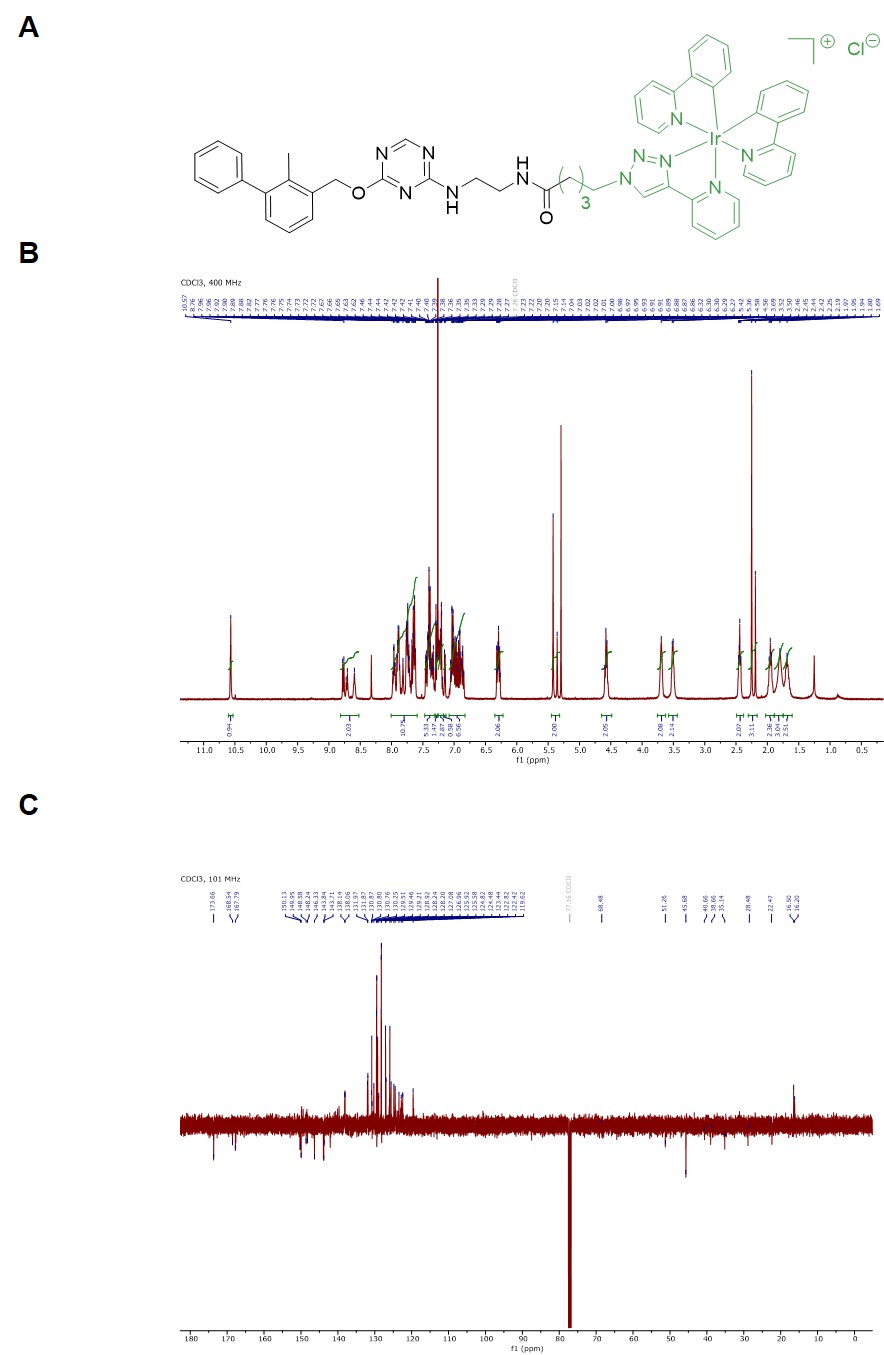
**Supplementary Figure S36. Spectroscopic characterization of disubstituted, “long” Ir(III) complex Co-4**. **(A)** Chemical structure. **(B)** ^1^H NMR (400 MHz, CDCl_3_). (C) ^13^C NMR (101 MHz, CDCl_3_).


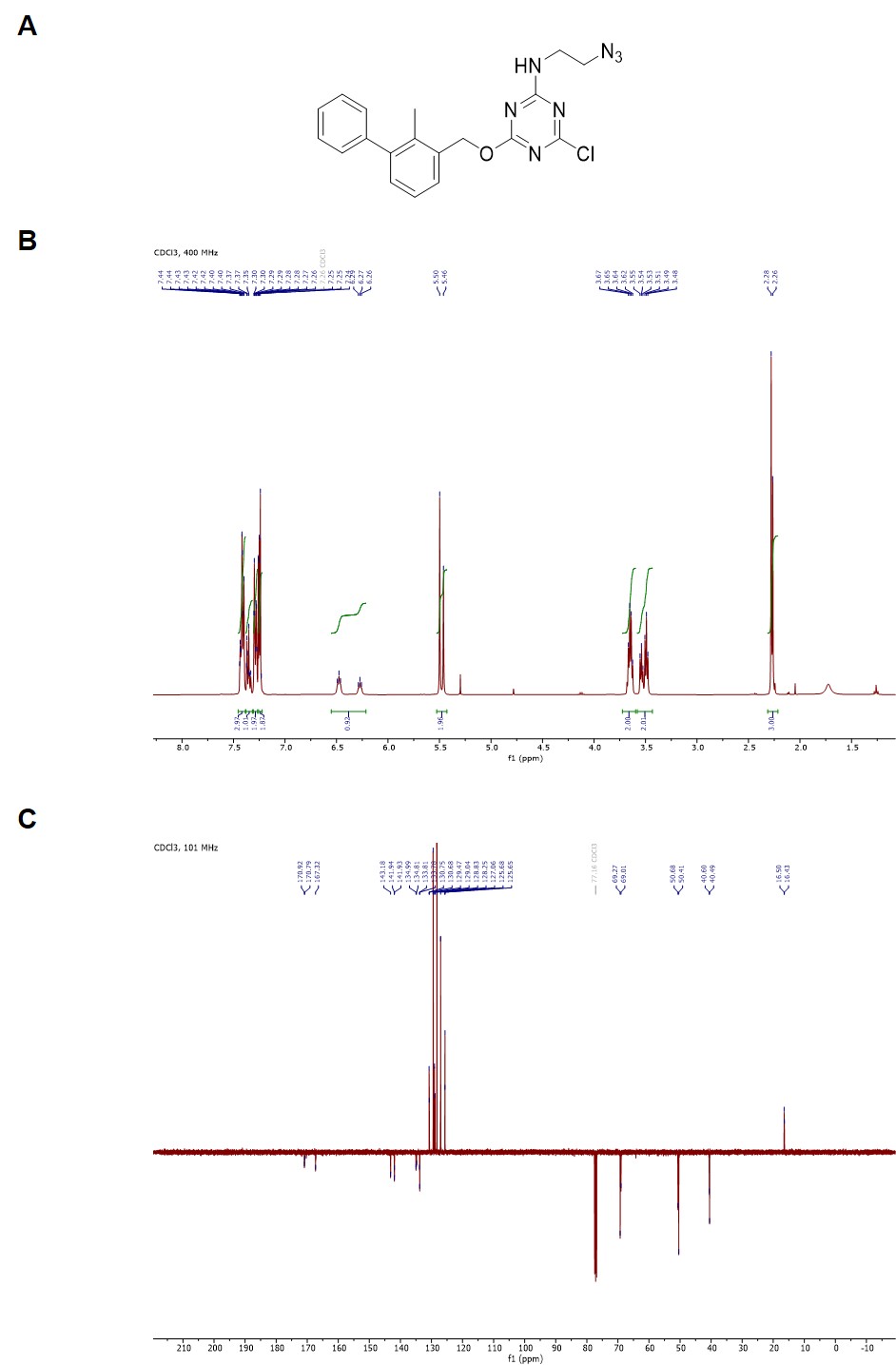


**Supplementary Figure S37. Spectroscopic characterization of N-(2-azidoethyl)-4-chloro-6-((2-methyl-[1,1'-biphenyl]-3-yl)methoxy)-1,3,5-triazin-2-amine 15**. **(A)** Chemical structure. **(B)** ^1^H NMR (400 MHz, CDCl_3_). (C) ^13^C NMR (101 MHz, CDCl_3_).


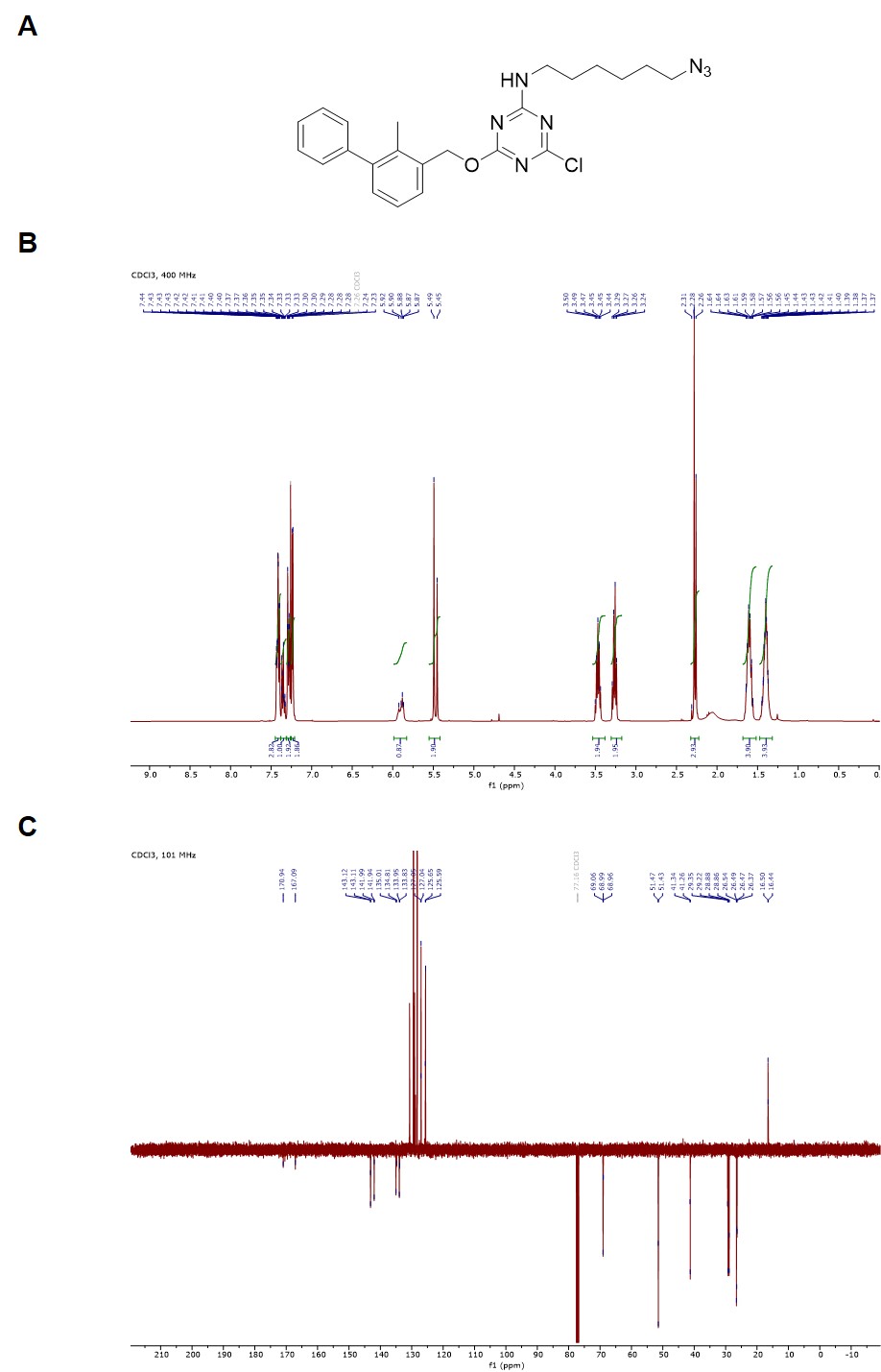


**Supplementary Figure S38. Spectroscopic characterization of N-(6-azidohexyl)-4-chloro-6-((2-methyl-[1,1'-biphenyl]-3-yl)methoxy)-1,3,5-triazin-2-amine 16**. **(A)** Chemical structure. **(B)** ^1^H NMR (400 MHz, CDCl_3_). (C) ^13^C NMR (101 MHz, CDCl_3_).


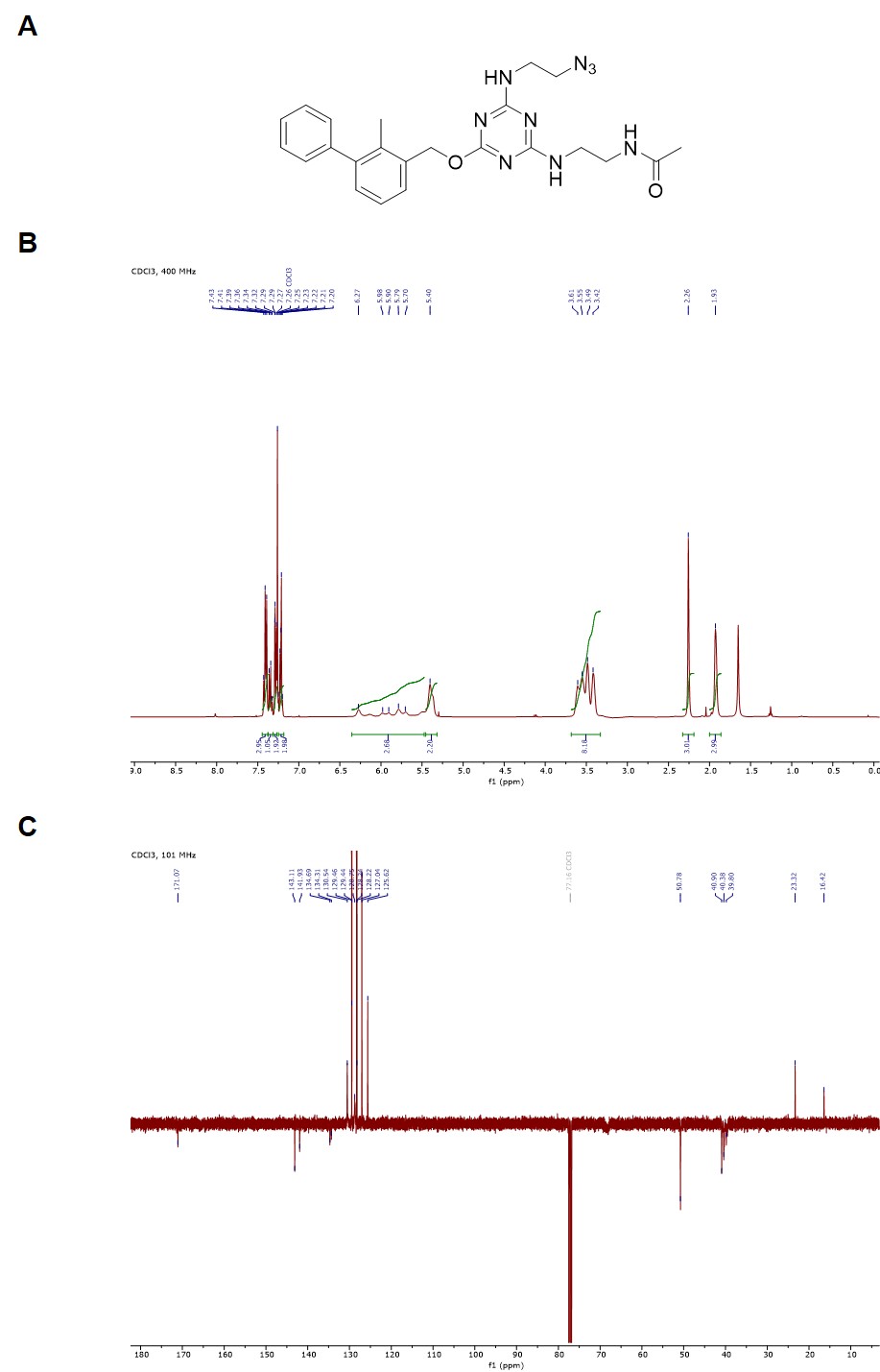


**Supplementary Figure S39. Spectroscopic characterization of N-(2-((4-((2-azidoethyl)amino)-6-((2-methyl-[1,1'-biphenyl]-3-yl)methoxy)-1,3,5-triazin-2-yl)amino)ethyl) acetamide 17**. **(A)** Chemical structure. **(B)** ^1^H NMR (400 MHz, CDCl_3_). (C) ^13^C NMR (101 MHz, CDCl_3_).


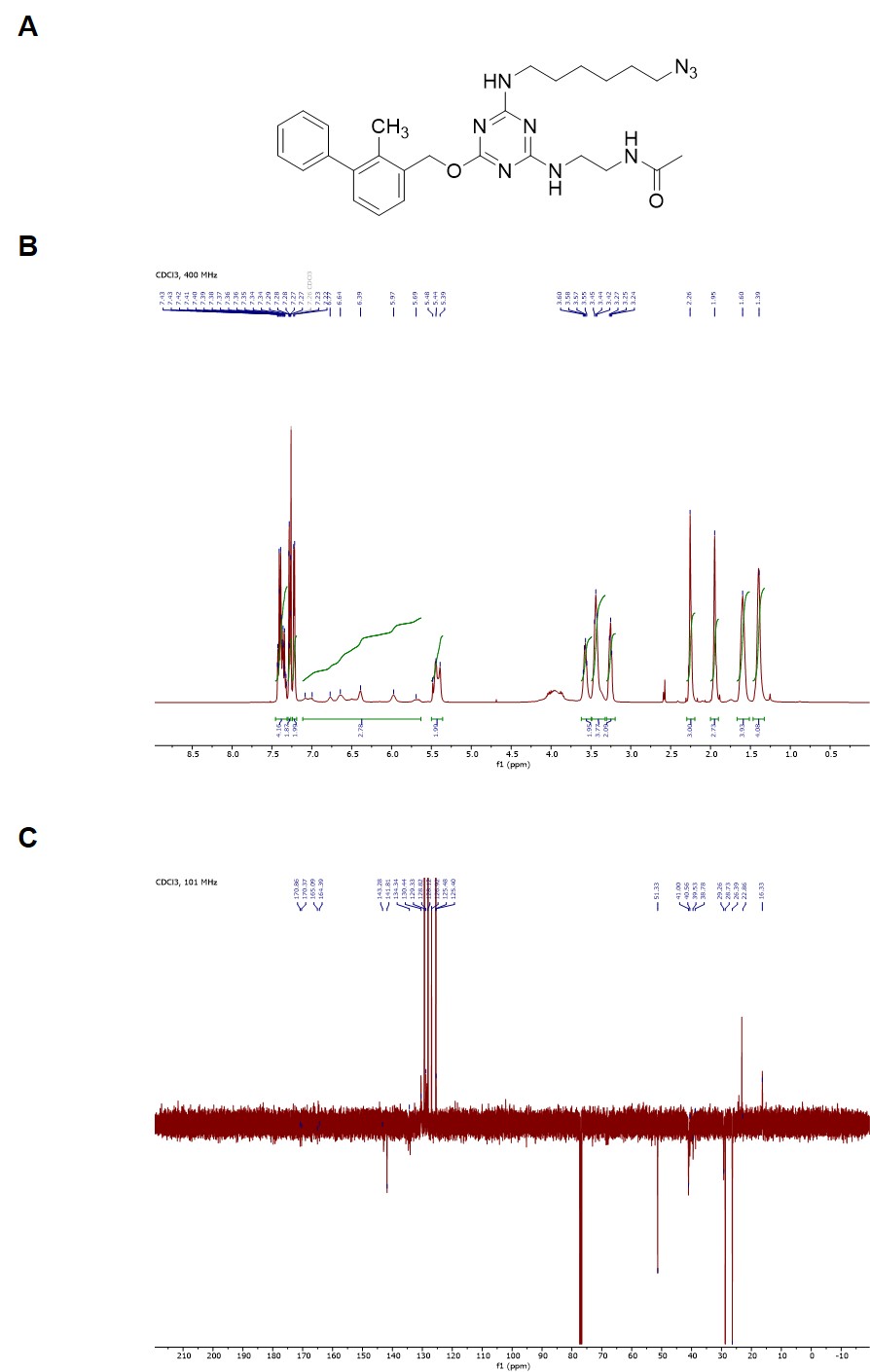


**Supplementary Figure S40. Spectroscopic characterization of N-(2-((4-((6-azidohexyl)amino)-6-((2-methyl-[1,1'-biphenyl]-3-yl)methoxy)-1,3,5-triazin-2-yl)amino)ethyl) acetamide 18**. **(A)** Chemical structure. **(B)** ^1^H NMR (400 MHz, CDCl_3_). (C) ^13^C NMR (101 MHz, CDCl_3_).


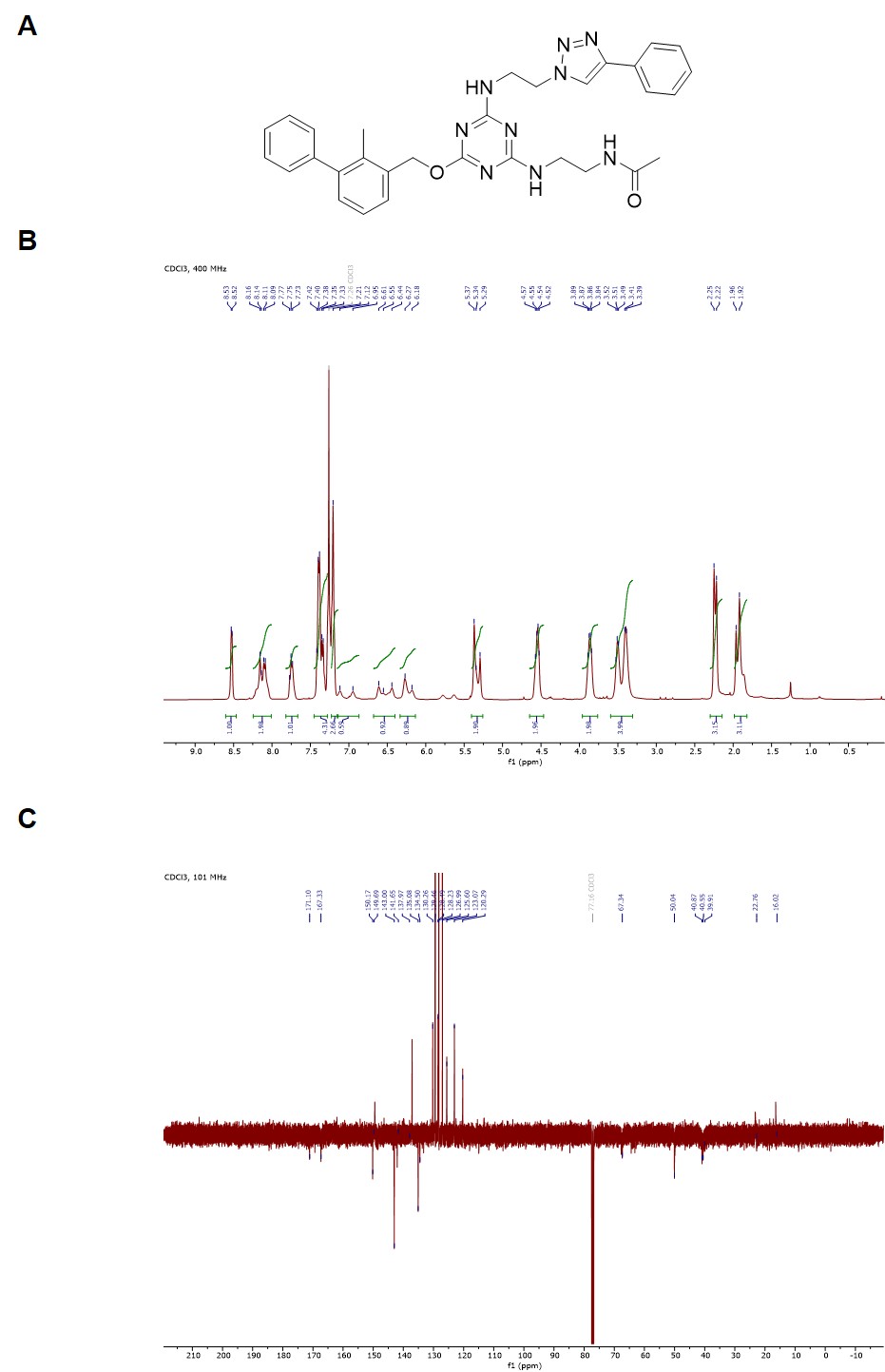


**Supplementary Figure S41. Spectroscopic characterization of N-(2-((4-((2-methyl-[1,1'-biphenyl]-3-yl) methoxy)-6-((2-(4-phenyl-1H-1,2,3-triazol-1-yl)ethyl)amino)-1,3,5-triazin-2-yl)amino)ethyl)acetamide 19**. **(A)** Chemical structure. **(B)** ^1^H NMR (400 MHz, CDCl_3_). (C) ^13^C NMR (101 MHz, CDCl_3_).


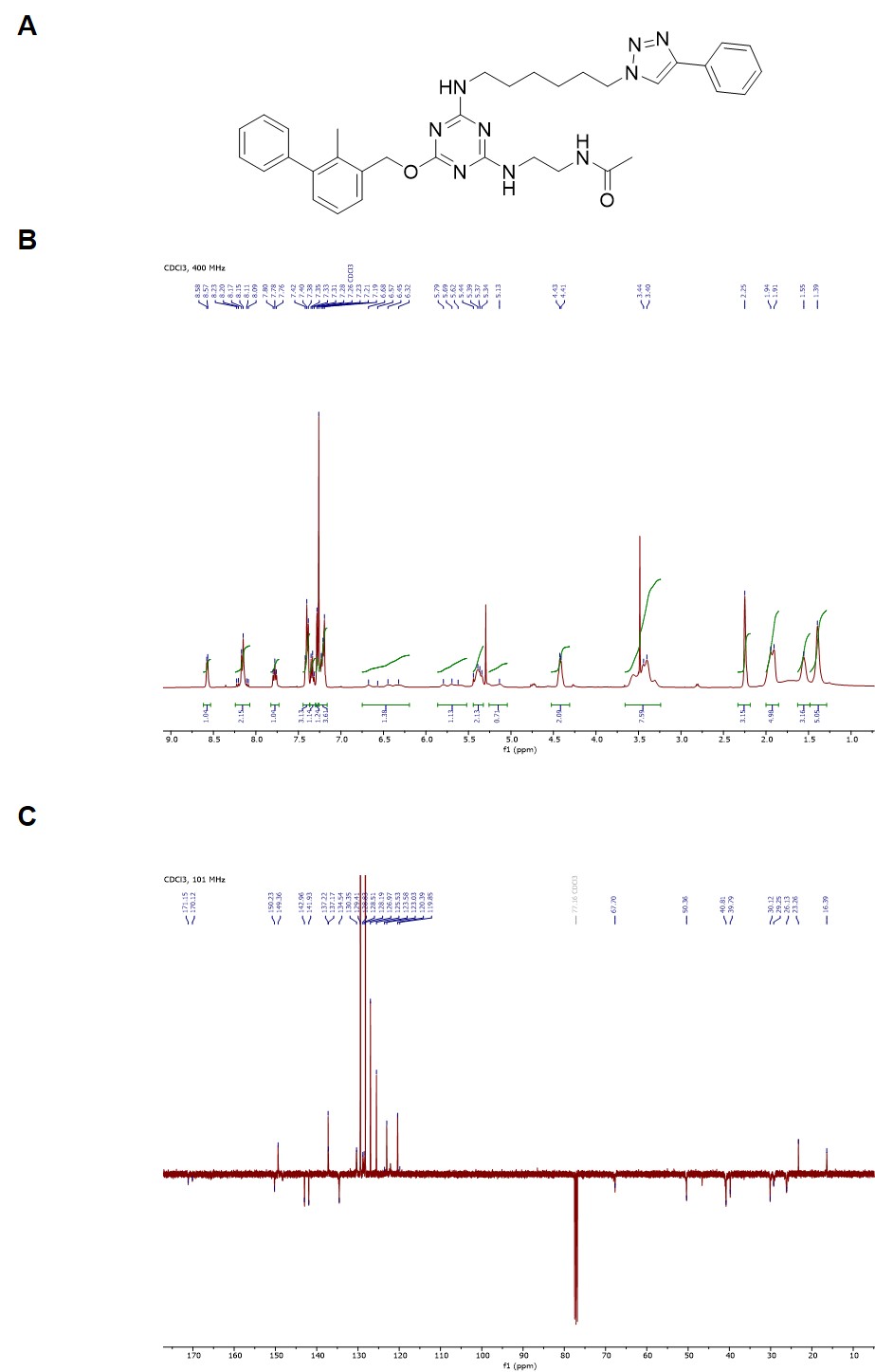


**Supplementary Figure S42. Spectroscopic characterization of N-(2-((4-((2-methyl-[1,1'-biphenyl]-3-yl)methoxy)-6-((6-(4-phenyl-1H-1,2,3-triazol-1-yl)hexyl)amino)-1,3,5-triazin-2-yl)amino)ethyl)acetamide 20**. **(A)** Chemical structure. **(B)** ^1^H NMR (400 MHz, CDCl_3_). (C) ^13^C NMR (101 MHz, CDCl_3_).


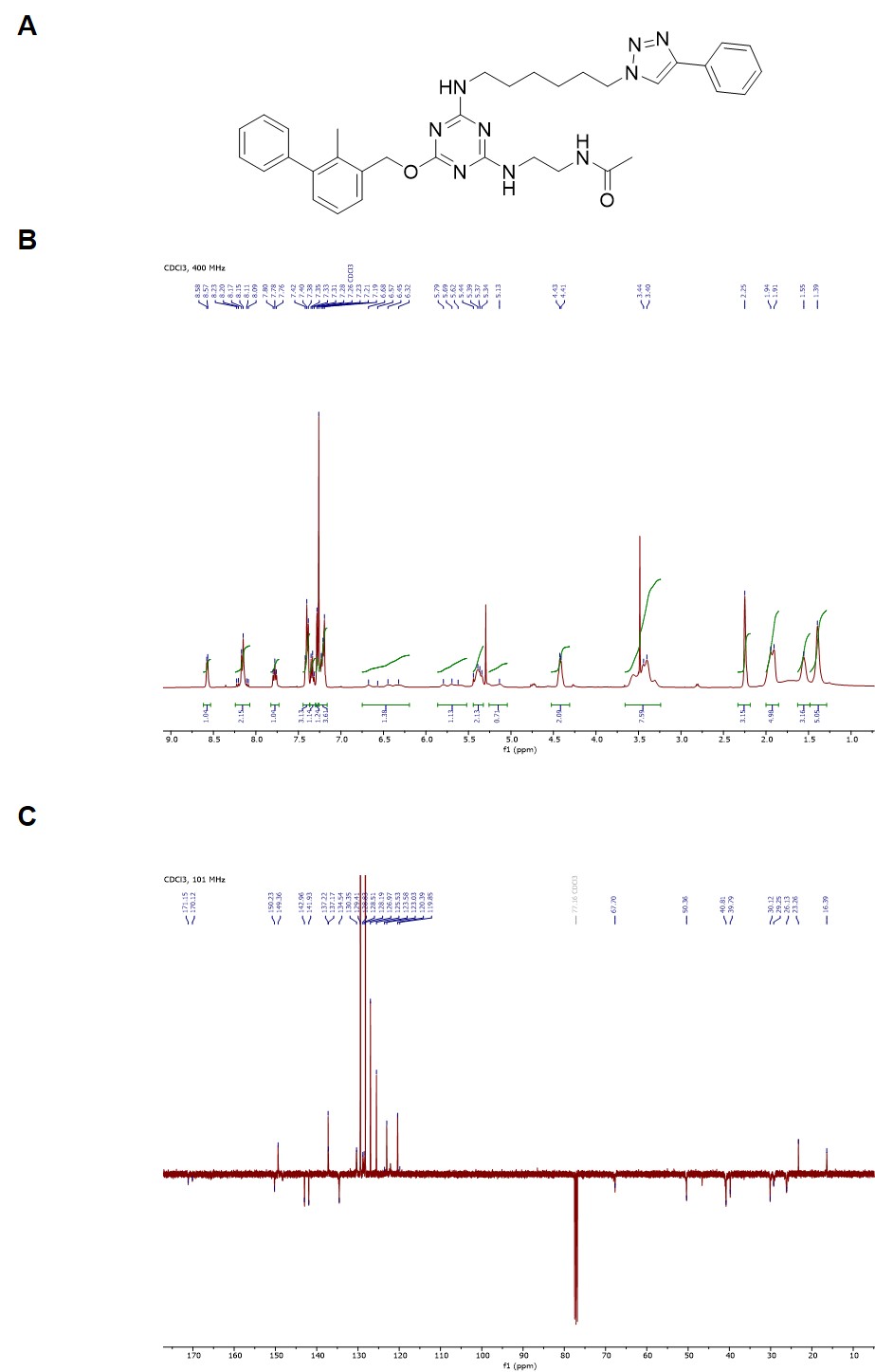


**Supplementary Figure S43. Spectroscopic characterization of trisubstituted, “short” Ir(III) complex conjugate Co-5**. **(A)** Chemical structure. **(B)** ^1^H NMR (400 MHz, CDCl_3_). (C) ^13^C NMR (101 MHz, CDCl_3_).


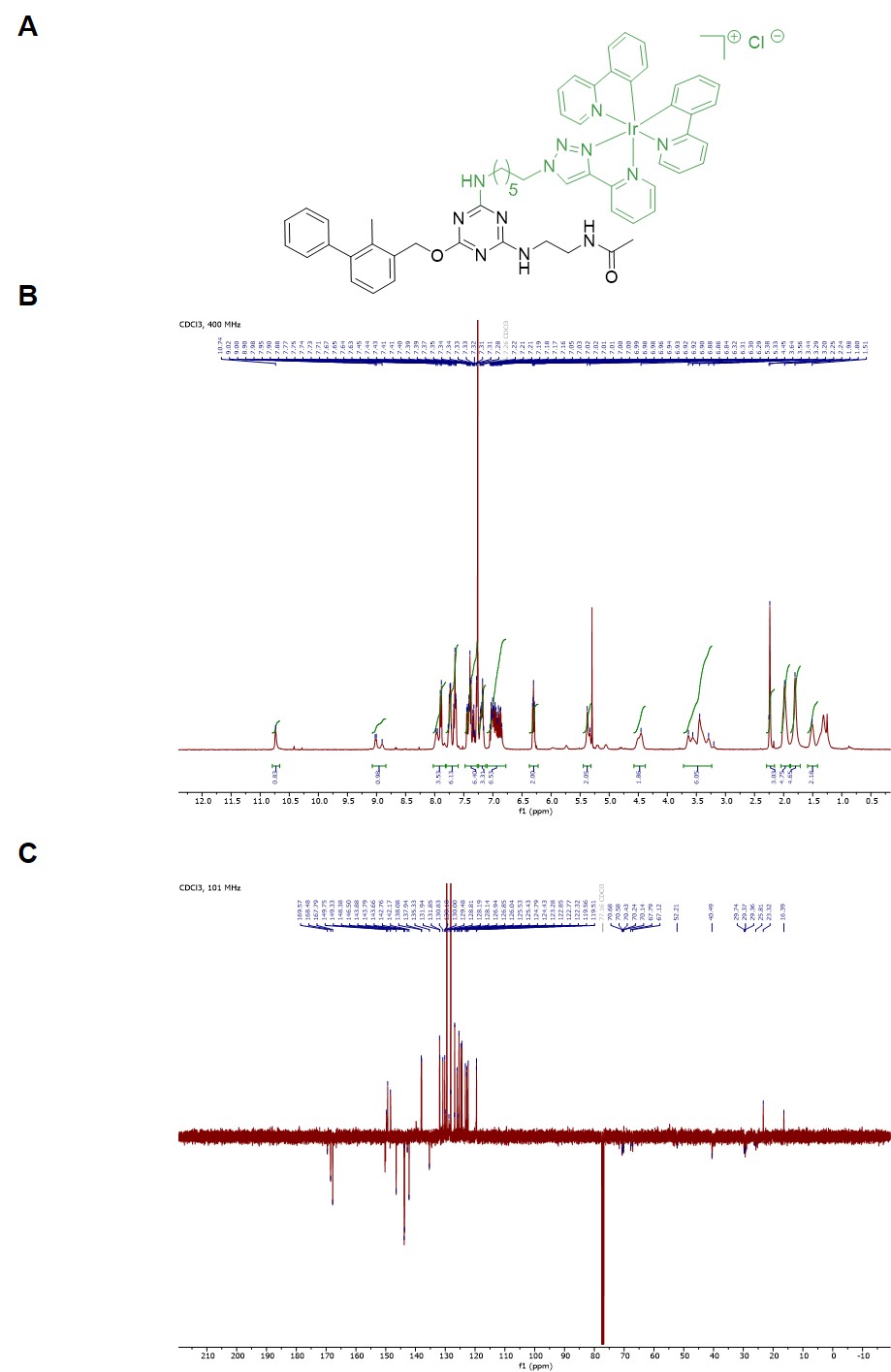
**Supplementary Figure S44. Spectroscopic characterization of Trisubstituted, “long” Ir(III) complex conjugate Co-6**. **(A)** Chemical structure. **(B)** ^1^H NMR (400 MHz, CDCl_3_). (C) ^13^C NMR (101 MHz, CDCl_3_).
